# Computational Study on the Reaction Mechanism of 5‐Enolpyruvylshikimate‐3‐phosphate Synthase from *Nicotiana Tabacum*

**DOI:** 10.1002/open.202400433

**Published:** 2025-01-07

**Authors:** Qingfang Han, Beibei Lin, Ziwei Liu, Mengsha Li, Zhaopeng Luo, Xixian Xie, Lijuan Ma, Hao Su, Xiang Sheng

**Affiliations:** ^1^ College of Biotechnology Tianjin University of Science and Technology Tianjin 300457 China; ^2^ Key Laboratory of Engineering Biology for Low-Carbon Manufacturing Tianjin Institute of Industrial Biotechnology Chinese Academy of Sciences Tianjin 300308 China; ^3^ Haihe Laboratory of Synthetic Biology Tianjin 300308 China; ^4^ National Center of Technology Innovation for Synthetic Biology Tianjin 300308 China; ^5^ Zhengzhou Tobacco Research Institute of CNTC Zhengzhou 450001 China

**Keywords:** 5-Enolpyruvylshikimate-3-phosphate synthase, Reaction mechanism, Quantum chemical calculation

## Abstract

5‐Enolpyruvylshikimate‐3‐phosphate synthase (EPSPS) catalyzes the conversion of 5‐enolpyruvate (PEP) and shikimic acid phosphate (S3P) to 5‐enolpyruvylshikimic acid‐3‐phosphate (EPSP), releasing inorganic phosphate. This reaction is the sixth step of the shikimate pathway, which is a metabolic pathway used by microorganisms and plants for the biosynthesis of aromatic amino acids and folates but not in mammals. In the present study, the detailed reaction mechanism of EPSPS from *Nicotiana tabacum* (*Nt*EPSPS) is revealed by quantum chemical calculations with the cluster approach. The reaction is proposed to involve the formation of a carbocation intermediate, the formation of a tetrahedral intermediate, the C−O bond cleavage and the re‐formation of C=C bond. All four steps are concerted processes involving proton transfer events. The calculations suggest a step‐wise mechanism for the formation of the tetrahedral intermediate by the proton transfer from the hydroxyl group of S3P to Asp331 and the nucleophilic attack of hydroxyl group on the carbocation, which is consistent with the proposal in literature. The energy profile for the entire reaction is presented, showing that C−O bond cleavage of the tetrahedral intermediate, releasing phosphate, is the rate‐limiting step. The interaction between the Glu359 residue and the phosphate group is significant in stabilizing the phosphate.

## Graphical abstract

The reaction mechanism of 5‐enolpyruvylshikimate‐3‐phosphate synthase from *Nicotiana tabacum* (*Nt*EPSPS) is elucidated using quantum chemical calculations. The proposed mechanism involves four sequential steps: carbocation intermediate formation, tetrahedral intermediate formation, rate‐limiting C−O bond cleavage, and C=C bond re‐formation. All these steps are concerted processes involving proton transfer events.

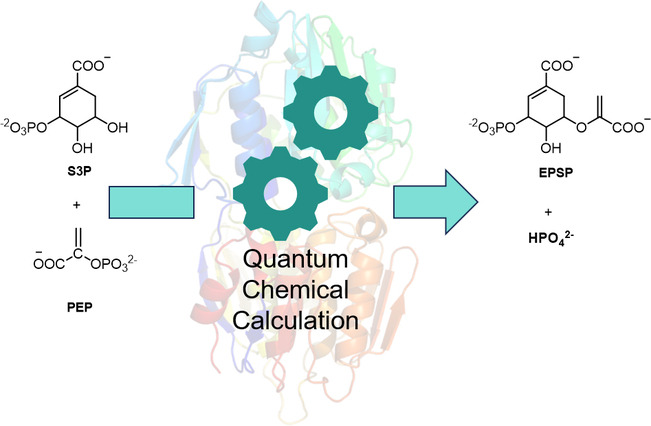

## 1 Introduction

**Scheme 1:**
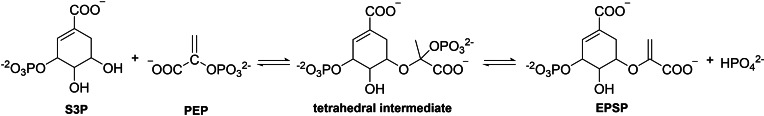
5‐enolpyruvylshikimate‐3‐phosphate synthase (EPSPS)‐catalyzed formation of 5‐enolpyruvateshikimate 3‐phosphate (EPSP) and phosphate (Pi) from phosphoenolpyruvate (PEP) and shikimate‐3‐phosphate (S3P).

The shikimate pathway, including a sequence of seven metabolic steps, is a crucial biochemical route present in microorganisms and plants, enabling the synthesis of aromatic compounds that are essential for various cellular functions and survival.^[1]^ In the sixth step of the shikimate pathway, phosphoenolpyruvate (PEP) participates by condensing with shikimate‐3‐phosphate (S3P), forming 5‐enolpyruvate shikimate 3‐phosphate (EPSP) and releasing inorganic phosphate (Pi). This reaction is catalyzed by 5‐enolpyruvylshikimate‐3‐phosphate synthase (EPSPS), as shown in Scheme 1.

Notably, the shikimate pathway does not exist in mammals, making it an attractive target for herbicides and antimicrobial agents, as its inhibition can selectively affect plants and microorganisms without harming mammals.[^2^, ^3^] In plants, for example *Nicotiana tabacum*, EPSPS is the enzyme targeted by N‐(phosphonomethyl)glycine, the active ingredient in the herbicide glyphosate.[^1^, ^4^, ^5^] Due to its importance as a herbicide target, EPSPS has been studied extensively across different organisms. A number of crystal structures of EPSPS has been resolved, including those from a few prokaryotes and the plant model species *Arabidopsis thaliana*.^[6]^ Most EPSPS proteins are monomeric, but in cyanobacteria, the enzyme often exists in a dimeric form.[^7^, ^8^, ^9^]

The reaction mechanism of EPSPS from *Escherichia coli* (*Ec*EPSPS) has been studied based on the crystal structure of the D313A mutant using the quantum mechanical/molecular mechanical (QM/MM) method.^[10]^ It has been proposed that the reaction follows an addition‐elimination mechanism involving a tetrahedral intermediate (Scheme 1), which has been confirmed experimentally.^[11]^ In the addition process, it is suggested that protonation of phosphoenolpyruvate (PEP) occurs before a nucleophilic attack on PEP. In the subsequent elimination process, the breaking of the C−O bond of the tetrahedral intermediate must happen before the proton is transferred from PEP to Glu341. Thus, both addition and elimination occur in a step‐wise manner. QM/MM calculations on the *Ec*EPSPS reaction revealed that the barriers for the two steps in the addition process are 16.3 kcal/mol and 11.3 kcal/mol at the level of M06‐2X/6‐31G(d)/MM, respectively.^[10]^ However, the elimination process exhibits higher energy barriers, with 19.2 kcal/mol for C−O bond cleavage and 18.6 kcal/mol for proton transfer. In the proposed mechanism, an aspartate residue (Asp313) and a glutamate residue (Glu341) are catalytically important, acting as general acid/base groups, and a lysine (Lys22) plays also a crucial role. These findings are consistent with previously reported mutation experiments.^[12]^

Although the mechanism of EPSPS has been studied, the initial step of the mechanism presents an unusual aspect. Namely, the hydroxyl group of S3P is present in its deprotonated form, which is uncommon since the p*K*_a_ of a hydroxyl group is typically above 9. In the present study, the reaction mechanism of the EPSPS‐catalyzed synthesis of 5‐enolpyruvateshikimate 3‐phosphate (EPSP) is re‐visited by using the quantum chemical cluster approach, which is a method has over the years proven to be valuable in enzymatic reaction mechanism.[^13^, ^14^, ^15^, ^16^, ^17^, ^18^] A large cluster model of the active site, consisting of over 400 atoms, was designed on the basis of predicted structure. Based on the calculations, a detailed reaction mechanism is proposed, which involves four steps, essentially consistent with the previously proposed addition‐elimination mechanism.^[10]^

## Computational Methods

### Model Setup and MD Simulations

The structure of EPSPS from *Nicotiana tabacum* (*Nt*EPSPS) was predicted by AlphaFold2.^[19]^ The ternary complex of *Nt*EPSPS with S3P and PEP was manually constructed. The positions of the two substrates were determined by aligning and overlapping the predicted structure of *Nt*EPSPS with the crystal structure of EPSPS from *Mycobacterium tuberculosis* that is bound with the same substrates (PDB ID: 2O0E).^[20]^

The protonation states of the residues and substrates were carefully considered during the construction of the model for molecular dynamics (MD) simulations. The APBS server was employed to determine the protonation states of ionizable residues, taking into account hydrogen bonding networks.^[21]^ Asp283, Asp331 and Asp402 were set in their deprotonated forms, while Lys23, Arg28, Asp50, Arg105, Arg131, Lys358, Glu359, Arg362, His403, Arg404 and Lys429 were protonated. For the phosphate group of the substrate S3P, it was modeled in its deprotonated form due to the stable hydrogen bond network with surrounding amino acid residues (Arg105, Ser179, Ser206, and Lys358), consistent with previous computational studies.^[10]^ Regarding the PEP substrate, whose phosphate group is released after the catalytic cycle, the p*K*a was estimated to be 8.3 (see Supporting Information), indicating that it prefers to be in its protonated state under experimental conditions.

All the missing hydrogen atoms in the system were added through the LEaP program. The built systems were then solvated in a TIP3P water[^22^, ^23^] box, and Na^+^ ions were added to neutralize the system. The AMBER force fields FF19SB and GAFF2 were used for protein and substrates, respectively. The resulting systems were subjected to a series minimizations and equilibrium. Afterwards, a MD simulation of 50 ns was carried out by the GPU version of AMBER 20 software package.^[24]^

### Quantum Chemical Cluster Approach

All quantum chemical calculations presented were performed using the Gaussian 16 program^[25]^ with the B3LYP‐D3(BJ) hybrid density functional method.[^26^, ^27^, ^28^, ^29^] Geometries were optimized with the def2‐SVP basis set. Single‐point energies were calculated at the same level of theory with the SMD solvation model^[30]^ (ϵ=4) to account for the effects of the enzyme surrounding environment. To obtain more accurate electronic energies, single‐point calculations were performed with the larger basis set def2‐TZVP[^31^, ^32^] based on the optimized structures. Frequency calculations were conducted at the same level of geometry optimization to obtain zero‐point energies (ZPEs). The energies presented in this article are thus the large basis set energies, including the dispersion effect, corrected for the solvation effect and ZPE. The optimized transition states were confirmed by the presence of an imaginary frequency, which corresponds to the motion along the reaction coordinate connecting the preceding and following intermediates.

## 2 Results and Discussion

### 2.1 Active Site Model

**Figure 1:**
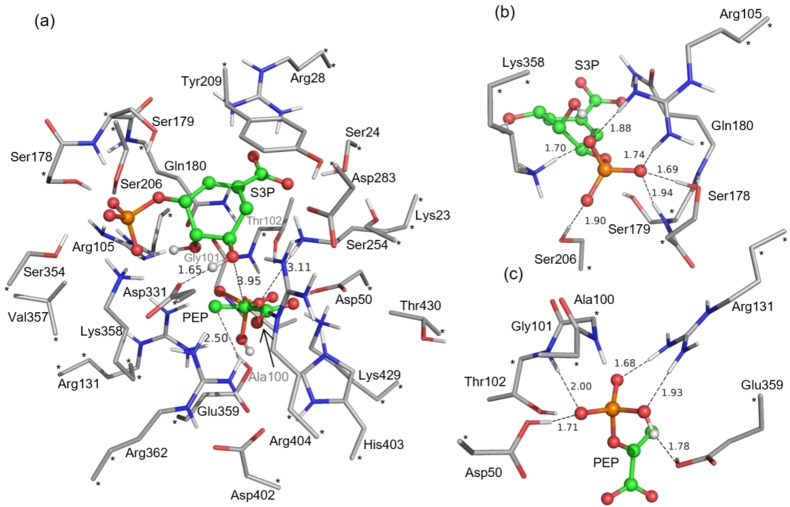
Optimized structure of the enzyme‐substrates complex of the active site model of *Nt*EPSPS. The PEP and S3P substrates are highlighted and all nonpolar hydrogens are omitted. Atoms fixed during the geometry optimization are marked with asterisks, and selected distances are given in angstrom. (a) The full cluster model, (b) the residues forming hydrogen bonds with S3P, and (c) the residues forming hydrogen bonds with PEP.

The most representative snapshot from MD simulations was used to build the quantum chemical (QC) cluster model to investigate the interaction between the substrates and the active site residues, as well as the reaction mechanism. By analyzing the hydrogen bond network and other interactions, the final active site model contains 425 atoms, consisting of residues Lys23, Ser24, Arg28, Asp50, Ala100, Gly101, Thr102, Arg105, Arg131, Ser178, Ser179, Gln180, Ser206, Tyr209, Ser254, Asp283, Asp331, Ser354, Val357, Lys358, Glu359, Arg362, Asp402, His403, Arg404, Lys429, Thr430 and the two substrates (PEP and S3P), as shown in Figure 1a. The overall charge of the model is +1. Most of the amino acid residues in the active site model were truncated, with the truncated carbon atoms and some associated hydrogen atoms kept fixed during the geometry optimizations to avoid unrealistic movements. These fixed atoms are marked with asterisks in the Figure 1.

In the optimized enzyme‐substrates complex (referred to E : S), the phosphate group of S3P is anchored by five hydrogen bonds with the side chains of Lys358, Arg105 and Ser206, and the backbone amide group of Ser179. The distances of these hydrogen bonds are 1.70 Å, 1.88 Å, 1.74 Å, 1.69 Å, 1.94 Å and 1.90 Å, respectively, as shown in Figure 1b. Similarly, the phosphate group of PEP forms five hydrogen bonds with the side chains of Glu359, Arg131, Asp50 and Thr102, with the bond distances of 1.78 Å, 1.93 Å and 1.68 Å, 1.71 Å and 2.00 Å, respectively, as shown in Figure 1c. A notable difference between the binding pockets of the phosphate groups of the two substrates is that more positively charged residues are present in the one of S3P.

### 2.2 Reaction Mechanism

**Scheme 2:**
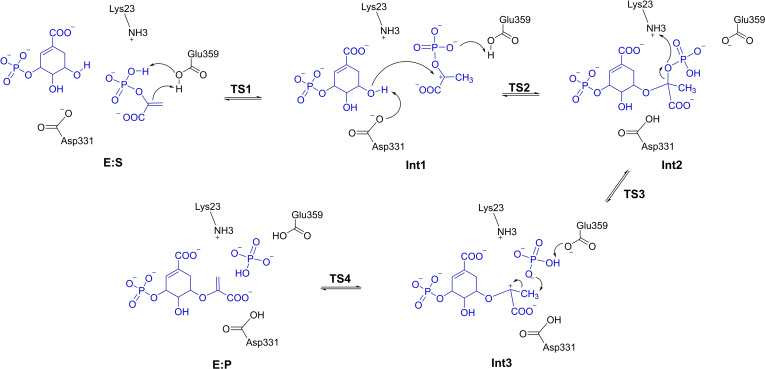
Reaction mechanism of *Nt*EPSPS proposed on the basis of the current calculations. Note that the proton transferring from Lys23 to the phosphate group at TS3 returns to Lys23 during the geometry optimization of Int3.

**Figure 2:**
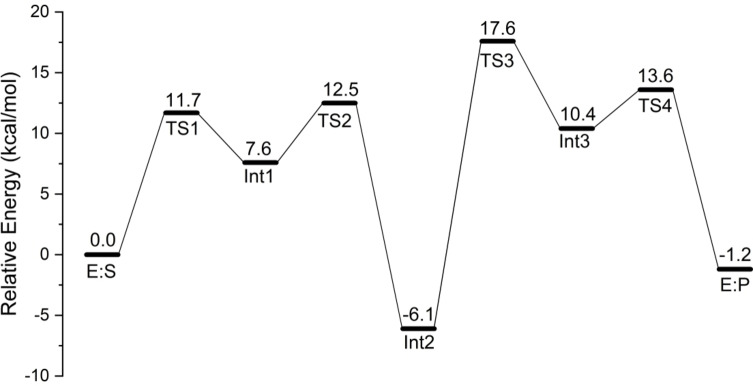
Calculated energy profile of *Nt*EPSPS‐catalyzed reaction.

**Figure 3:**
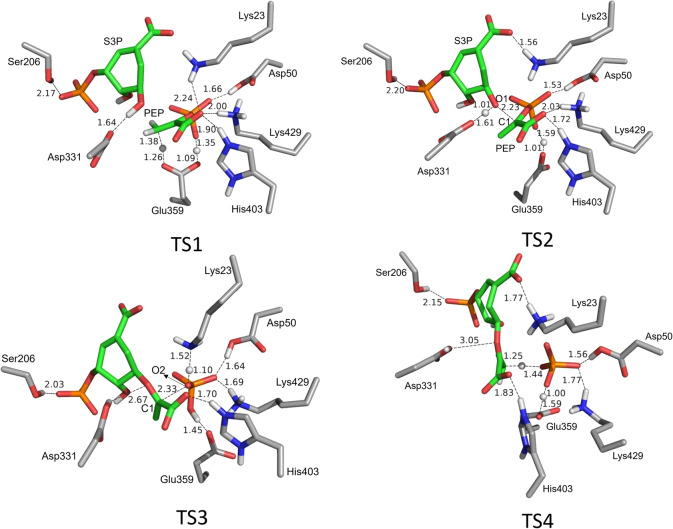
Optimized structures of the transition states in the suggested mechanism for the *Nt*EPSPS‐catalyzed reaction. Note that for clarity only a part of the active site model is shown here and nonpolar hydrogens are omitted. For full models see Supporting Information. Distances are given in angstrom.

Based on the obtained structure of the enzyme‐substrates complex (E : S, Figure 1a), the mechanism of *Nt*EPSPS‐catalyzed addition‐elimination reaction was studied in detail. The detailed mechanism is shown in Scheme 2, and the calculated energy profile is shown in Figure 2. The optimized structures of the transition states are shown in Figure 3, and structures of the intermediates are given in the Supporting Information.

Starting from E : S, the first step of the reaction is the proton transfer from Glu359 to the C=C double bond of PEP, forming the carbocation intermediate (Int1), which is 7.6 kcal/mol higher than E : S. Meanwhile, proton of the phosphate group of PEP transfers to Glu359, avoiding the reaction of the formed carbocation and the negatively‐charged carboxylate group of Glu359. The barrier for this step is calculated to be 11.7 kcal/mol.

Next, a nucleophilic attack occurs between the carbocation generated in the previous step and the hydroxyl oxygen of S3P, resulting in the formation of a stable tetrahedral intermediate (Int2) with a calculated energy of 6.1 kcal/mol lower than that of E : S (Figure 2). The barrier of this step is 4.9 kcal/mol relative to Int1. This stability suggests that Int2 serves as a significant intermediate in the pathway, consistent with prior experimental confirmation of the presence of this intermediate in the reaction.^[11]^ Concurrently, two proton transfer events take place. One is that Asp331 acquires a proton from the hydroxyl group of the substrate S3P, and the other one is that Glu359 donates a proton to the phosphate group oxygen anion on PEP. These dual proton transfers suggest a finely tuned interplay between catalytic residues in managing the reaction pathway. At the transition state of this step (TS2), the distance between C1 and O1 is 2.23 Å (as shown in Figure 3 for TS2).

Continuing from Int2, the reaction proceeds by a C−O bond cleavage between the oxygen of phosphate group (marked as O2 in Figure 3) and the carbon of the substrate PEP (marked as C1 in Figure 3). According to the calculations, the C−O bond cleavage occurs concertedly with a proton transfer from Lys23 to the oxygen (O2) of the phosphate group, leading to the formation of a free phosphate group and a new carbocation intermediate (Int3). As shown in Figure 2, this is the rate‐determining step of the overall reaction, with an barrier of 23.7 kcal/mol relative to Int2. At TS3, the breaking C−O bond distance is 2.33 Å, while the distance between the transferring proton and the oxygen of the phosphate group is 1.10 Å. Notably, the proton transferring from Lys23 to the phosphate O2 at TS3 returns to Lys23 in the optimized structure of Int3.

In the final step, a proton transfer takes place from the ‐CH_3_ group of the carbocation intermediate to the free phosphate group to form a C=C bond, concerted by another proton transfer from the oxygen of phosphate group to Glu359, resulting in E : P via TS4. The barrier for this step is only 3.2 kcal/mol relative to Int3. The obtained enzyme‐product complex E : P is calculated to be 1.2 kcal/mol lower in energy than E : S.

The *Nt*EPSPS‐catalyzed reaction pathway, as revealed by the present quantum chemical study, consists of four elementary steps, all of which are concerted processes involving the simultaneous formation or cleavage of two or three bonds. In the rate‐limiting step, the C−O bond cleavage takes place concertedly with a proton transfer from Lys23 to the oxygen (O2) of the phosphate group (Int2 to Int3 via TS2, as shown in Scheme 2). The calculated overall reaction barrier is 23.7 kcal/mol. Due to the lack of kinetic data for *Nt*EPSPS, a direct comparison with experimental values is not possible. However, for *Ec*EPSPS, the measured *k_cat_* is 14 s^−1^, which corresponds to an estimated barrier of approximately 16 kcal/mol. This suggests that the barrier calculated in this study may be somewhat overestimated.

## 3 Conclusions

We have employed the quantum chemical cluster approach to investigate the detailed mechanisms of the addition‐elimination reaction between PEP and S3P catalyzed by EPSPS from *Nicotiana tabacum* (*Nt*EPSPS). A detailed mechanism is proposed, in which the reaction starts with the proton transfer from Glu359 to the PEP double bond concertedly by a proton transfer from the PEP phosphate group to Glu359, forming the carbocation intermediate. Next, cocorrently with two proton transfer events, the nucleophilic attack of the hydroxyl oxygen of S3P at the carbocation occurs, resulting in the formation of a stable tetrahedral intermediate. Subsequently, the C−O bond between the PEP carbon and the phosphate oxygen breaks, resulting in the formation of a new carbocation intermediate and a free phosphate group. Finally, a proton transfer takes place from the −CH_3_ group to the free phosphate group, which concurrently gives a proton to Glu359, generating the EPSP product and the inorganic phosphate. The calculations showed that the step of C−O bond cleavage to release the inorganic phosphate group is rate‐limiting step. The current calculations enhanced the mechanistic understanding of EPSPS‐catalyzed reaction. We believe that the obtained insights provided by the current study is helpful in designing new EPSPS inhibitors.

## Conflict of Interests

The authors declare no conflict of interest.

## Supporting information

As a service to our authors and readers, this journal provides supporting information supplied by the authors. Such materials are peer reviewed and may be re‐organized for online delivery, but are not copy‐edited or typeset. Technical support issues arising from supporting information (other than missing files) should be addressed to the authors.

Supporting Information

## Data Availability

The data that support the findings of this study are available in the supplementary material of this article.
