# Supplementary material for: Computational Study on the Reaction Mechanism of 5‐Enolpyruvylshikimate‐3‐phosphate Synthase from Nicotiana Tabacum
Source: ChemistryOpen. 2025 Jan 7;14(7):e202400433. doi: 10.1002/open.202400433 (PMC12256929; doi:10.1002/open.202400433)
Supplement: Supplementary file 1 — Supporting Information [file OPEN-14-e202400433-s001.pdf]

ChemistryOpen

Supporting Information

Computational Study on the Reaction Mechanism of 5-Enolpyruvylshikimate-3-phosphate Synthase from *Nicotiana Tabacum*

Qingfang Han, Beibei Lin, Ziwei Liu, Mengsha Li, Zhaopeng Luo, Xixian Xie, Lijuan Ma, Hao Su,* and Xiang Sheng*

Table of Contents

1. The pK_a estimation of the PEP substrate S2

2. Optimized structures of intermediates and transition states S3

3. Cartesian coordinates S7

1. The pK_a estimation of the PEP substrate

The p*K*a value of acetic acid in aqueous solution was taken from previous study (measured to be 4.76) and was used as a reference. All the calculations were carried out at the B3LYP/def2-SVP level of theory. “H” represents that the phosphate group of PEP is protonated, “D” represents that the phosphate is deprotonated.

Deprotonation energy of the PEP substrate：

$\Delta E_1 = E(E{:}H)_{\varepsilon=4} - E(E{:}D)_{\varepsilon=4}$ = 286.51 kcal/mol

Deprotonation energy of the acetic acid in aqueous solution:

$\Delta E_2 = E(CH_3COOH)_{\varepsilon=78} - E(CH_3COO^-)_{\varepsilon=78}$ = 281.7 kcal/mol

The pK_a of enzyme-bound substrate:

$\Delta pK_a = (\Delta E_1 - \Delta E_2)/2.303RT = 3.54$,

3.54 + 4.76 = 8.3

2. Optimized structures of intermediates and transition states

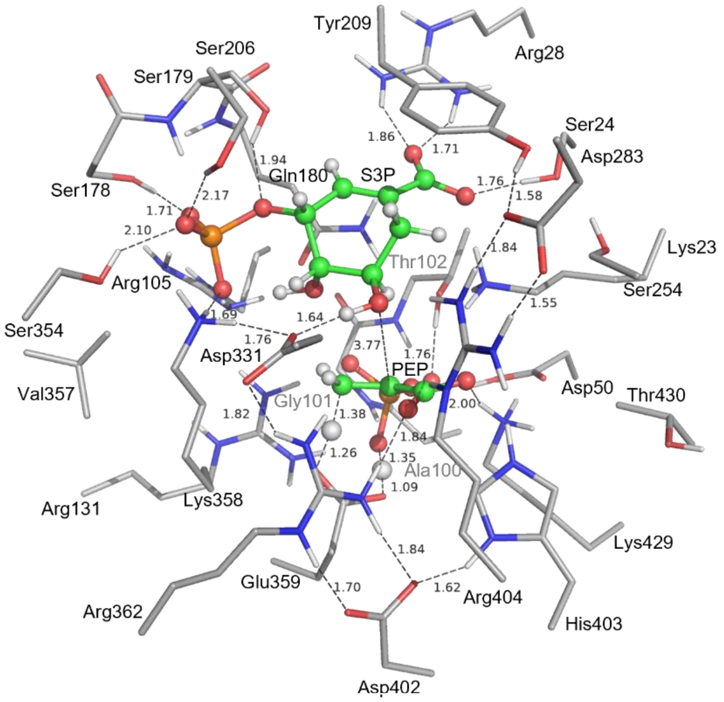

TS1(11.7)

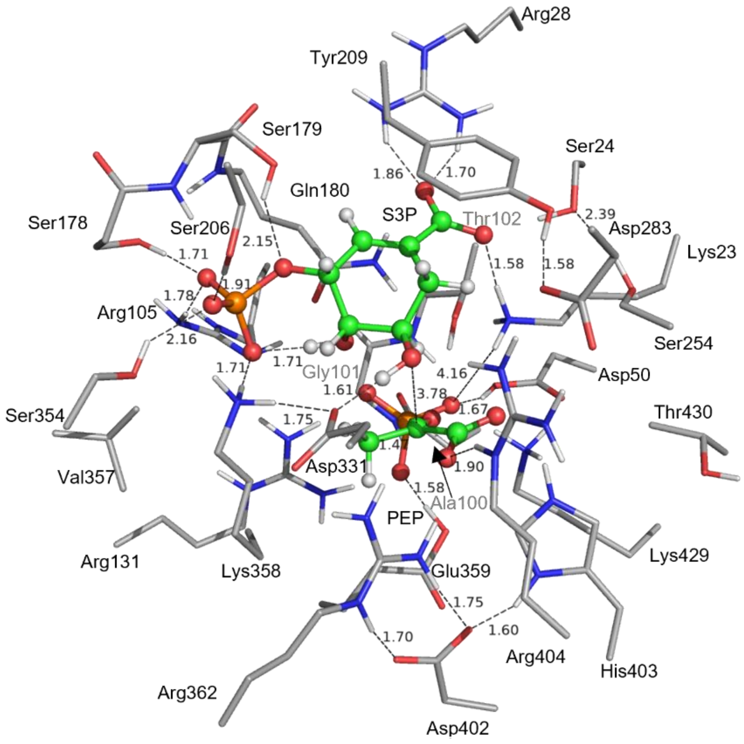

Int1(7.6)

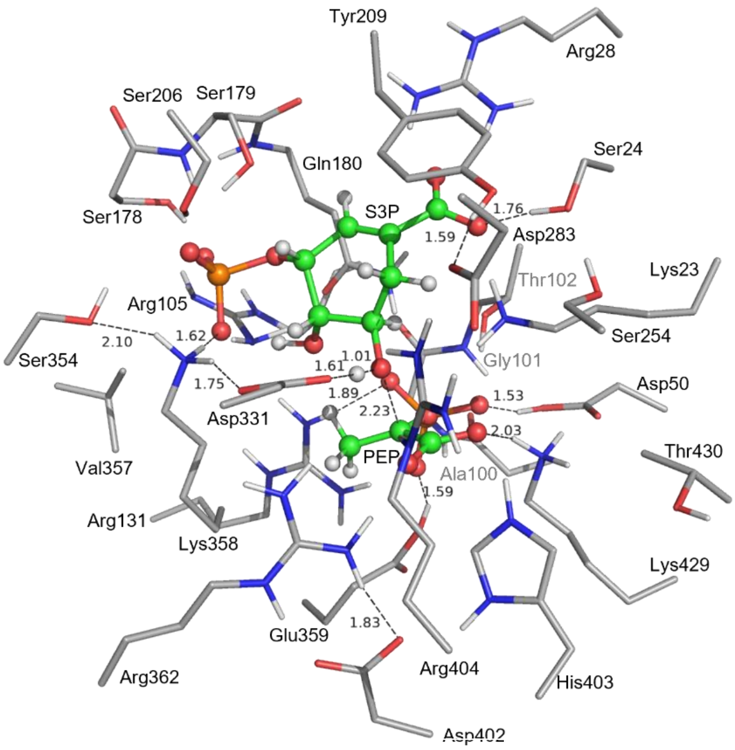

TS2(12.5)

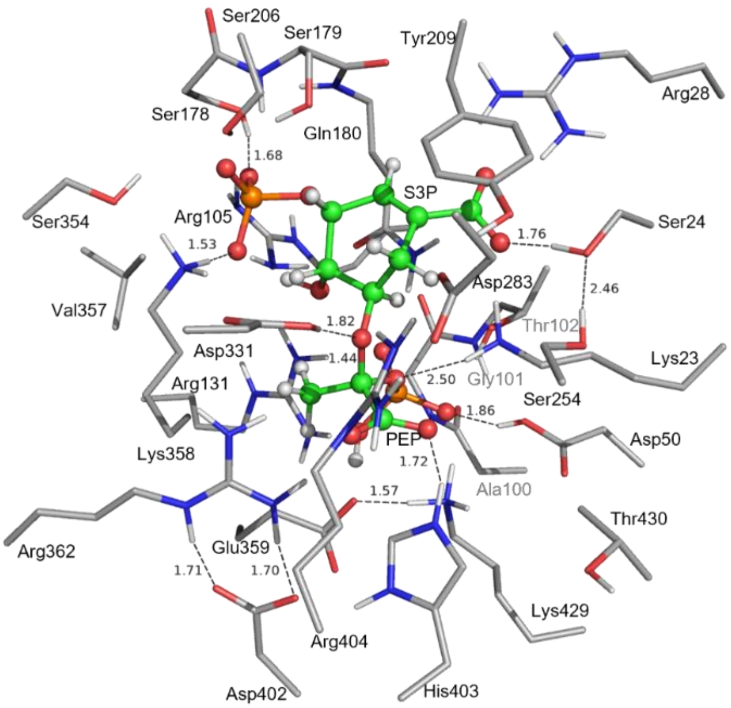

Int2(-6.1)

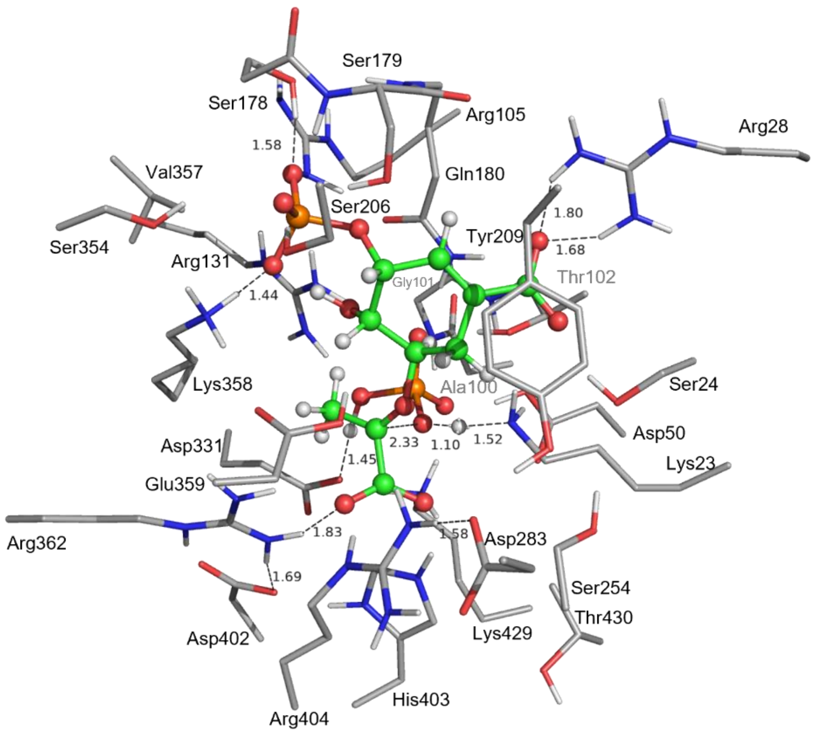

TS3(17.6)

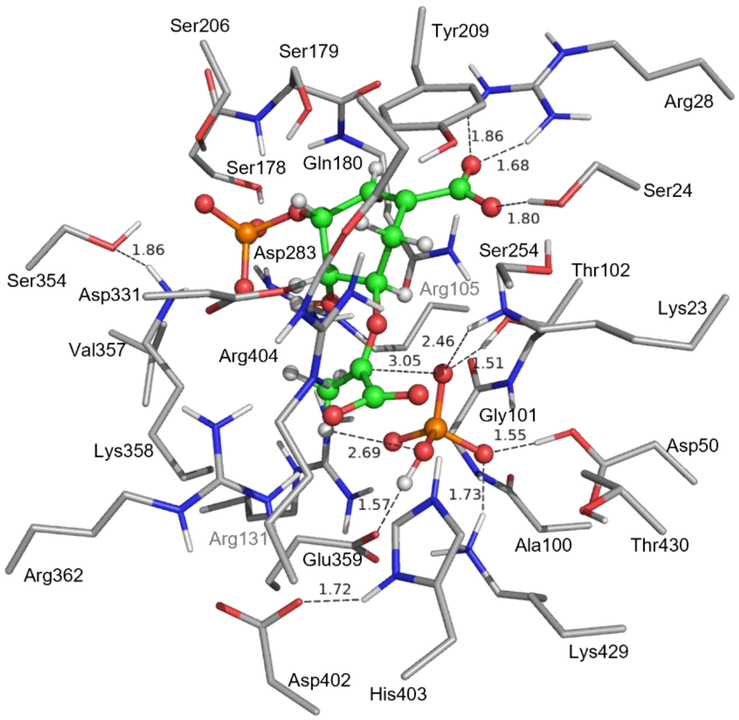

Int3(10.4)

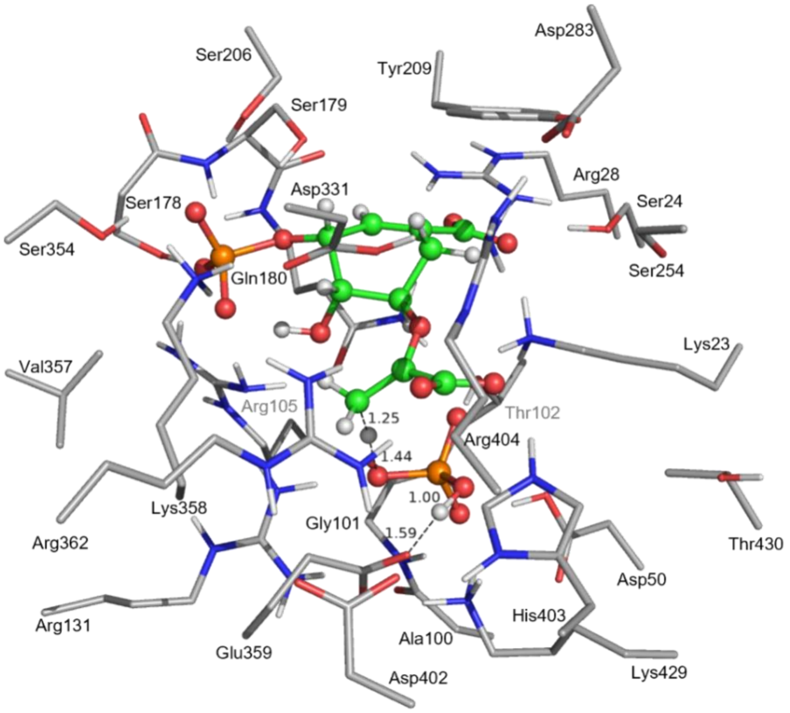
Asp283
Ser206
Tyr209
Ser179
Arg28
Ser24
Ser178
Asp331
Ser354
Gln180
Ser254
Lys23
Val357
Arg105
1.25
Thr102
Arg404
1.44
1.00
Thr430
Lys358
Gly101
1.59
Arg362
Asp50
His403
Arg131
Ala100
Glu359
Asp402
Lys429

TS4(13.6)

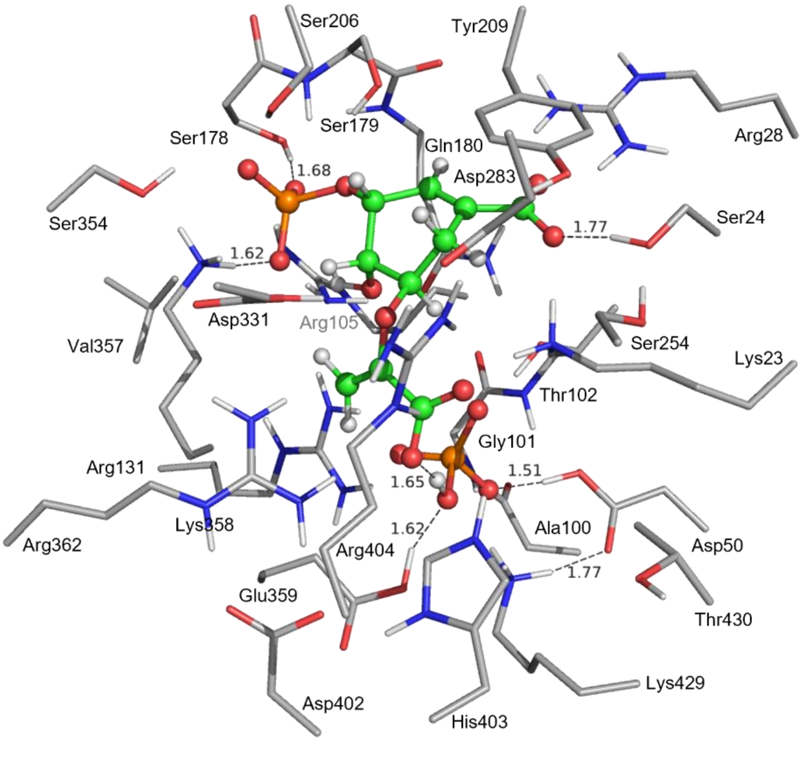
Ser206
Tyr209
Arg28
Ser179
Ser178
Gln180
1.68
Asp283
Ser354
1.77
Ser24
1.62
Asp331
Arg105
Val357
Ser254
Lys23
Thr102
Gly101
Arg131
1.51
1.65
Lys358
Ala100
Arg362
1.62
Asp50
Arg404
1.77
Glu359
Thr430
Lys429
Asp402
His403

EP(-1.2)

3. Cartesian coordinates

E:S(0.0)

C	47.75494300	43.77607200	38.46498500
C	47.88666900	42.50881900	37.61510600
C	48.79500300	41.39841800	38.17928500
C	48.10373000	40.34284900	39.05722700
C	48.97429800	39.09604900	39.16220200
N	48.39967700	38.05513400	40.06790300
H	47.08874600	44.50865300	37.98274300
H	46.88545400	42.08569500	37.41206800
H	48.28217100	42.80769400	36.62889500
H	49.25478200	40.87261400	37.32955400
H	49.64271300	41.82688800	38.73866500
H	47.92064700	40.73616500	40.06696100
H	47.13090200	40.06422700	38.61319200
H	49.09515400	38.62776600	38.17451100
H	49.98245100	39.35202100	39.52387900
H	47.37081600	37.93271300	39.96279800
H	48.83625300	37.14646100	39.86070000
H	48.54805400	38.30604500	41.10378300
C	47.94699600	42.62099400	42.12200400
C	49.29391600	42.10956200	42.63484400
O	49.85035100	41.11974400	41.79043800
H	47.19310100	41.81628100	42.11864900
H	50.00625700	42.95098300	42.68223000
H	49.19033900	41.72248600	43.66405400
H	49.54340000	40.25655500	42.13558500
C	42.49300700	45.08401100	45.30399600
C	43.72498700	44.78699700	46.18800500
C	44.04190000	43.27841100	46.17969300
C	45.28590000	42.86466200	46.98017100
N	45.40992600	41.42119400	47.20622600
C	45.94625300	40.52204700	46.35770100
N	46.41308900	40.88705700	45.16286300
N	46.01677700	39.22592400	46.69077200
H	41.69295700	44.35309700	45.50797000
H	44.59663000	45.36759400	45.83929700
H	43.53267400	45.10697500	47.22650200
H	43.17511600	42.73459700	46.59336300
H	44.14211700	42.92688900	45.13684000
H	46.21029000	43.22862000	46.50091200
H	45.26380700	43.33454400	47.97506500

H	45.02368400	41.05254400	48.06722800
H	46.31622100	41.83315300	44.82510800
H	46.82730900	40.12836100	44.57296800
H	45.87170000	38.88638700	47.63660900
H	46.52143100	38.61598600	46.03295900
C	42.94391200	40.46507100	35.22494100
C	44.10973300	40.31258200	36.22453700
C	44.74692700	38.95605200	36.42357700
O	45.96545800	38.79580300	36.40560800
O	43.90416300	37.97871500	36.70414900
H	42.20627300	39.65768600	35.34079800
H	44.92599800	41.00901200	35.98882000
H	43.74405700	40.58367500	37.23193400
C	41.40499900	35.44400600	35.83098400
C	41.00872100	35.06540700	37.25063700
O	39.90465800	35.29057600	37.71067200
C	40.70151600	36.71776600	35.37104000
H	42.50099700	35.56290100	35.77370900
H	41.02348800	37.57665300	35.97966900
H	40.92533900	36.93672100	34.31608500
H	39.61424500	36.61318500	35.49049300
N	41.98599500	34.43960100	37.98295100
C	41.77773200	34.18678300	39.39025400
C	41.92517600	35.40903000	40.30717500
O	41.30987500	35.47771000	41.36031300
H	42.91861900	34.39047600	37.59478800
H	40.75767100	33.81190700	39.54015800
H	42.49930800	33.43481100	39.73539500
N	42.79868400	36.35657000	39.89814600
C	43.11199500	37.48600100	40.74396500
C	44.38089900	38.24140100	40.30673600
C	44.72856400	39.36056200	41.27642400
O	45.53769300	37.40218800	40.18057700
H	43.32506300	36.20531400	39.04478400
H	43.18414900	37.15224100	41.78903400
H	44.17191100	38.68001200	39.31142000
H	45.38894600	36.83762400	39.39424300
H	43.87296300	40.03849000	41.41358700
H	45.00924400	38.94864000	42.25673800
H	45.57901100	39.94660200	40.89763700
C	38.77101000	34.70903200	43.65699800
C	40.18797100	34.06399900	43.77697700
C	40.41687600	32.91468500	42.79113100
C	41.88116000	32.50753700	42.61786900

N	42.44415000	31.89680500	43.83054600
C	43.72875900	31.57832500	44.02735800
N	44.65895400	31.85504700	43.07083700
N	44.12420400	30.93058900	45.11972700
H	37.99257800	33.92646900	43.63082100
H	40.94872000	34.83530900	43.59333900
H	40.33858800	33.70492800	44.81270000
H	39.80555800	32.03409000	43.06627000
H	40.07386200	33.23866200	41.79821500
H	41.96964000	31.78279400	41.78858300
H	42.46713100	33.39987300	42.36456100
H	41.81409300	31.75516900	44.61227800
H	44.56651600	32.84268700	42.76122900
H	45.63538400	31.67276300	43.38487000
H	43.50124100	30.82466100	45.91799100
H	45.10488700	31.13478000	45.41519500
C	41.02900500	25.29698000	40.27296300
C	41.20597100	26.91104500	40.03499400
C	42.26301700	27.18499600	38.96130400
C	42.46879800	28.64226700	38.54061600
N	43.18658700	29.44149900	39.53716000
C	43.79309700	30.61546000	39.27946600
N	43.76843400	31.11664800	38.03387900
N	44.43352100	31.29090800	40.22678000
H	42.00587100	24.83404800	40.47510100
H	40.23306800	27.33623200	39.74000500
H	41.48510000	27.36829200	41.00025400
H	43.23674800	26.76684200	39.27179500
H	41.97953500	26.63789000	38.04696400
H	43.06510300	28.64424300	37.61441900
H	41.49365500	29.11077000	38.31204400
H	43.16551100	29.13351800	40.50299300
H	43.27452300	30.64267300	37.29240000
H	44.33121200	31.93452300	37.78883300
H	44.41886000	31.03577300	41.21995800
H	44.69721600	32.28141200	40.04339000
C	46.45286000	30.40996400	48.86603900
C	46.69063000	31.67223700	49.71391400
O	46.60487800	31.68109100	50.92870900
C	44.95487800	30.22907600	48.61665300
O	44.42321000	31.31752300	47.86653900
H	46.94208500	30.54233100	47.88963900
H	44.78930100	29.27421300	48.07853600
H	44.40628200	30.17495800	49.57039700
H	45.14529700	31.60969900	47.26297700
N	46.89635000	32.80914500	48.96040000
C	46.86599600	34.16004900	49.50071400
C	45.74844900	35.03866400	48.90350500
O	45.73301700	36.25275200	49.08755600
C	48.16159300	34.94418800	49.27538700
O	48.35864300	35.25985100	47.91101100
H	46.88416600	32.69197900	47.94483500
H	46.69386800	34.05864700	50.58471300
H	48.07653000	35.90380200	49.80073100
H	49.00534000	34.38107100	49.70513400
H	48.35102200	34.46099700	47.36004300
N	44.84921600	34.39079300	48.13050200
C	43.75798100	35.09196900	47.49690700
C	43.66170400	34.83088400	45.99192600
C	44.91676900	35.33280100	45.26832000
C	44.82029900	35.37741800	43.75657400
N	45.43761300	36.40548800	43.14964400
O	44.20837300	34.51787900	43.10821300
H	44.81379200	33.37365200	48.16384300
H	43.93336600	36.16185500	47.68015500
H	43.52569500	33.75589000	45.79877100
H	42.76213200	35.33375900	45.59970400
H	45.18953500	36.33028600	45.64802100
H	45.76527700	34.66905200	45.50926200
H	45.52876500	36.38903900	42.13509000
H	45.99352200	37.08115200	43.67266900
C	51.82916200	33.65807500	49.29998600
C	50.91748400	33.47459400	48.08662000
O	51.30648300	32.35960600	47.31419800
H	52.87796400	33.57305400	48.96893400
H	49.88145300	33.32942200	48.42617300
H	50.92407300	34.40817700	47.48422200
H	50.50347400	32.03453300	46.86154600
C	49.79100700	39.12699700	48.44100500
C	49.55465400	38.37656600	47.11948200
C	50.62925700	38.63970400	46.08823300
C	50.62268300	39.84750700	45.36944400
C	51.67761100	37.74681600	45.82538500
C	51.60544900	40.15475000	44.43542600
C	52.67403400	38.03486900	44.88379600
C	52.65143500	39.25037100	44.17382300
O	53.57125300	39.60596700	43.26573100
H	50.11037800	40.16400400	48.24242600

H	49.44606400	37.29840000	47.30980000
H	48.59507400	38.70363400	46.69120900
H	49.81523600	40.56476000	45.55213900
H	51.71443100	36.79543300	46.36515900
H	51.59214100	41.08975000	43.87556900
H	53.48133900	37.32337900	44.69266600
H	54.17936000	38.85514100	42.97704800
C	54.54499900	40.14800700	38.94599000
C	53.37091700	39.74807100	39.84320900
O	52.19531900	40.52779400	39.58985500
H	55.33979700	39.39054600	39.04568700
H	53.14111800	38.67911200	39.66490700
H	53.65348200	39.82302600	40.90295100
H	51.78409900	40.76163600	40.43713800
C	57.82600900	39.04401400	43.41102000
C	56.92675200	39.34655200	42.18353800
C	56.16560300	38.12527400	41.64682900
O	56.68897300	37.49799000	40.69588700
O	55.04774500	37.80817200	42.17245300
H	58.00294200	37.96013000	43.50733200
H	56.19934000	40.13363200	42.43614600
H	57.54508900	39.70855000	41.35017700
C	54.90494300	31.34002100	42.21903400
C	53.65993900	32.16868200	41.95191200
C	52.37777400	31.35889600	42.05457000
O	52.42528800	30.11486800	41.98201500
O	51.27274600	32.01015600	42.10626900
H	54.84097500	30.36985600	41.70860800
H	53.58371800	33.05044200	42.60712600
H	53.66934100	32.57493300	40.92396500
C	50.15100100	26.19802000	46.21392100
C	50.20517900	27.61100700	46.79226100
O	49.39083900	28.46572200	45.99697100
H	49.13476600	25.78163600	46.30300900
H	49.85466400	27.60109500	47.84153600
H	51.25170100	27.97264100	46.80681900
H	49.23926100	29.33039400	46.42388300
C	45.55198000	26.43195600	42.70694900
C	45.24713700	27.02896200	44.08996200
C	45.20101100	25.94373800	45.16918700
C	46.24091000	28.13471900	44.46520400
H	44.79075900	25.69739300	42.39749600
H	44.24211200	27.49173500	44.03425500
H	46.18585000	25.45667900	45.27120000
H	44.93936900	26.36771300	46.15181700
H	44.46260800	25.16113000	44.93039300
H	47.25650000	27.73946000	44.62553000
H	46.29924500	28.92337300	43.69765600
H	45.95029000	28.62711600	45.40604000
C	46.96503400	28.39859800	39.70136200
C	48.43860300	28.11450500	39.38449100
C	49.25357100	27.92525900	40.66407500
C	49.20556300	29.15821500	41.55414000
C	49.80364300	28.94204200	42.92851700
N	49.77683600	30.23147400	43.64169200
H	46.79223700	29.42066700	40.06978900
H	48.51128400	27.20310800	38.76800600
H	48.88347200	28.92451900	38.78509000
H	48.85908700	27.05296500	41.21649700
H	50.30180100	27.68412900	40.42171100
H	49.73658000	29.98868500	41.06309100
H	48.16384500	29.48981700	41.69172300
H	49.23023800	28.21751200	43.51936900
H	50.84871700	28.61702200	42.86017900
H	50.38577300	30.92686600	43.14642200
H	48.81072900	30.65805600	43.65835600
H	50.03417800	30.14690300	44.63144900
C	45.80683000	28.83366100	36.11185600
C	47.32369600	29.03760700	36.14901700
C	47.81534600	30.34904500	36.73174900
C	47.60924300	31.54675300	35.85101100
O	47.17753700	31.58692900	34.73537500
O	47.94149800	32.73917500	36.47034400
H	45.30661900	29.53800200	35.42998700
H	47.77963200	28.22693900	36.73368500
H	47.73628900	28.92862100	35.13639600
H	47.34157900	30.56534700	37.70684500
H	48.89828000	30.29702500	36.92221300
H	48.25508900	32.58367600	37.38526700
C	52.34799000	24.35400100	37.72099800
C	52.87200200	25.63799500	38.39000200
C	52.05253700	26.84145800	37.88199200
C	52.47576500	28.21909000	38.38766700
N	51.75436900	29.27105700	37.67038400
C	51.48789700	30.48620600	38.14518600
N	51.69982800	30.81158100	39.42103100
N	50.98352400	31.43627300	37.33812500
H	52.27406700	24.48336400	36.62947200

H	52.81019200	25.55927200	39.48868000	H	54.62472300	34.67265900	36.37912000
H	53.93667300	25.78625700	38.14091500	H	52.99797100	34.00916900	36.23445000
H	52.10517100	26.85393000	36.77887200	H	53.43764800	32.51753900	38.22402200
H	50.98615000	26.69075500	38.12515700	H	55.01480800	33.25877400	38.42936700
H	52.28312600	28.30203600	39.46901200	H	52.37489300	34.41096800	39.06070700
H	53.56429500	28.35599300	38.25137100	H	55.73919700	35.33242300	38.54479500
H	51.46030600	29.13203000	36.68379900	H	55.90332500	36.34336200	40.01356100
H	51.99214700	30.16252600	40.15853500	H	52.59201100	35.55573400	41.08626500
H	51.39398400	31.71781000	39.76240200	H	54.01235200	36.63013900	41.28880900
H	50.94846500	31.33356700	36.30681300	C	47.10500900	38.22986600	31.48502500
H	50.82936600	32.35084800	37.76867800	C	47.78852100	36.87077200	31.63093600
C	50.01095600	30.57599500	31.79201700	C	47.97768700	36.38163400	33.07031300
C	49.68828800	29.73652300	33.02137000	C	46.71490100	36.30042100	33.92885200
C	50.33519100	30.17908200	34.33291000	C	46.97917900	35.74028200	35.32311400
O	50.55924100	31.42521600	34.53606200	N	47.69355100	36.71658400	36.20299300
O	50.56170500	29.29539300	35.18411000	H	46.02969200	38.16365300	31.71882800
H	49.56663800	31.58051600	31.86845600	H	47.21683800	36.11604100	31.06276000
H	48.59896800	29.75131200	33.20377800	H	48.78376600	36.91072600	31.15720800
H	49.96441300	28.68242600	32.87319600	H	48.43938600	35.38148200	33.05166400
C	51.93003600	33.85203600	31.53901900	H	48.71494800	37.03870000	33.55950300
C	51.29241500	35.12956200	32.07912800	H	46.22966400	37.28650000	34.01737800
C	51.12045100	35.11521400	33.56285500	H	45.98096700	35.63582700	33.44370100
C	51.24227200	36.12971000	34.48744600	H	46.04107000	35.50677600	35.83648100
N	50.68868400	33.99631000	34.26259100	H	47.58250600	34.82429900	35.27388500
C	50.53913600	34.31606300	35.54336300	H	47.02997900	37.49152200	36.45631100
N	50.85643400	35.60185100	35.70950900	H	48.10731700	36.28822600	37.07049300
H	52.09116500	33.91990300	30.45402400	H	44.37566500	37.16049500	37.02529400
H	51.89283600	36.01383000	31.81683900	C	49.70006500	40.63303700	33.03404600
H	50.30401200	35.28212100	31.61217800	C	50.52597800	40.24329100	34.26097200
H	50.58267600	32.96878500	33.99941800	C	49.72780400	39.32942600	35.18252600
H	51.54914100	37.16720500	34.35689800	O	51.69084000	39.49981700	33.90680000
H	50.22420000	33.64255400	36.32330300	H	48.66362900	40.85723800	33.33597800
H	50.76635000	36.03879700	36.64158000	H	50.81239100	41.14789200	34.83055300
C	54.69397700	32.85208300	34.18403600	H	52.30030400	40.07398000	33.42474700
C	54.56601100	32.58898400	35.74400200	H	48.79457200	39.80119000	35.51923900
C	54.02100100	33.76588600	36.56243600	H	49.47311900	38.41954400	34.61711400
C	53.99209500	33.45567200	38.06914600	H	50.32842500	39.05114900	36.06115100
N	53.38672600	34.46501900	38.92638200	P	47.69716200	32.09481800	45.39454100
C	54.07890600	35.34886400	39.69482000	C	49.07864400	34.06978500	43.14195200
N	55.35834500	35.61153700	39.43851100	C	49.83510500	35.10387800	42.25911200
N	53.46600200	35.94961800	40.71521500	C	48.90953400	34.53157500	44.61951700
H	55.30538600	33.75095800	34.00087800	C	50.09125900	36.43993300	42.96654500
H	53.92169000	31.70712800	35.90495500	C	48.90604600	36.85189000	43.79229100
H	55.56661500	32.31664300	36.12082500	C	48.39580000	35.94009300	44.63804100

O	47.97158300	33.74137800	45.35827500
O	46.37091100	32.04284900	46.18649700
O	48.90495100	31.41468700	46.04283800
O	47.43977600	31.62354500	43.91602700
O	47.81632700	33.84645300	42.55055300
O	51.05776300	34.62083600	41.73557000
C	48.23154300	38.16407600	43.57857800
O	47.27081600	38.49817700	44.33812400
O	48.56582800	38.86159300	42.57118900
H	47.50282300	33.00335900	42.95874700
H	51.15325600	33.64038400	41.88434900
H	50.96173900	36.30976300	43.62747500
H	50.37412400	37.21521400	42.24295100
H	49.12695700	35.28984600	41.42855600
H	49.66396800	33.14030400	43.15678200
H	49.90360300	34.46730400	45.10549200
H	47.57163100	36.16431300	45.31172900
P	45.54748300	34.53532000	38.48062700
C	48.20140200	34.11460300	39.04876900
C	48.08637100	33.03635600	39.83358300
C	49.52875900	34.68407800	38.59101200
O	47.15151100	34.87746000	38.56364100
O	44.97618600	35.85745100	37.96154900
O	44.97890300	33.90553600	39.71242000
O	45.45595300	33.42179600	37.28039200
O	50.52672100	33.93277000	38.63144800
O	49.50963900	35.86896600	38.13226800
H	48.99372300	32.54916500	40.18857700
H	47.12316100	32.68762700	40.19778600
H	42.74302900	45.00696000	44.23385400
H	42.06261500	46.07430200	45.48591900
H	43.29415900	40.48142200	34.18306800
H	42.44909000	41.41250200	35.46233400
H	47.18219100	38.59759600	30.45058400
H	50.03110700	41.51384900	32.47408900
H	49.66537200	39.77889400	32.33994400
H	47.52295700	39.00086300	32.14083000
H	40.57062200	24.82567400	39.39243100
H	40.38218100	25.17568400	41.14817700
H	38.50188500	35.38754400	44.47319900
H	38.70879300	35.26872600	42.71062000
H	51.35259100	24.07403300	38.09951500
H	53.04423200	23.52999300	37.90887300
H	50.85414600	25.50838400	46.69258200
H	50.40556000	26.23012000	45.14135700
H	55.81157700	31.86517300	41.87883500
H	55.02478100	31.15420800	43.29145400
H	54.97980300	41.11895000	39.20528100
H	54.22914400	40.18187800	37.88960400
H	58.81860800	39.49863300	43.32695800
H	57.36270200	39.38047100	44.35004200
H	51.73136800	34.63180100	49.79112600
H	51.65839700	32.86401400	50.04443800
H	48.89447600	39.18829100	49.06670700
H	50.59170500	38.65419800	49.03111400
H	46.89421200	29.49652800	49.27822100
H	42.84610400	34.83678400	48.04684900
H	55.20838400	32.00161700	33.71106400
H	53.74424900	32.98302200	33.65500900
H	49.63339800	30.10836400	30.87670900
H	51.30519800	32.97071000	31.73224800
H	51.09894200	30.69539900	31.66774500
H	52.89625500	33.69835700	32.03069900
H	48.73805400	44.25644700	38.59776100
H	47.36082300	43.57813200	39.46724500
H	48.09320600	42.96906900	41.09414000
H	47.57379400	43.46275700	42.72979900
H	41.15734900	34.61072100	35.16516300
H	42.31602100	38.23452100	40.67217200
H	45.68704900	27.81226100	35.73520100
H	45.35421900	28.90824600	37.11122500
H	46.53286000	25.92641400	42.70160900
H	45.59401400	27.23430500	41.96299000
H	46.67300200	27.69782100	40.48974500
H	46.31119600	28.23678300	38.83350000
H	48.47730600	37.15282500	35.70734400
H	46.29075000	33.33596700	36.76811900

TS1(11.7)

C	47.75493400	43.77612100	38.46497400
C	47.89420800	42.50663900	37.61968500
C	48.79893800	41.39953500	38.19636000
C	48.09809900	40.33895900	39.06091100
C	48.96981600	39.09366100	39.17126100
N	48.39555400	38.05329700	40.07800300
H	47.08579300	44.50417300	37.97999200
H	46.89476200	42.08227300	37.41056900
H	48.29684300	42.80253800	36.63548300

H	49.27698200	40.87788400	37.35425700
H	49.63438500	41.83104300	38.77149600
H	47.90235000	40.72771000	40.07013600
H	47.13091000	40.06020000	38.60447000
H	49.09342900	38.62395900	38.18438400
H	49.97621900	39.35205300	39.53574100
H	47.36513400	37.92992500	39.97713200
H	48.83479000	37.14639500	39.86956000
H	48.55314600	38.29805200	41.11497300
C	47.94699800	42.62099800	42.12199600
C	49.28787300	42.09438900	42.63699400
O	49.83744000	41.10013700	41.79358600
H	47.18350300	41.82507300	42.11798600
H	50.00729200	42.92977900	42.68948200
H	49.17709900	41.70587100	43.66472800
H	49.55161600	40.23666700	42.15619400
C	42.49301400	45.08402600	45.30399400
C	43.72498900	44.78698700	46.18799100
C	44.03797300	43.27504400	46.16408500
C	45.28239600	42.83466700	46.95119100
N	45.38389500	41.38631800	47.16576900
C	45.93469500	40.48361900	46.32883600
N	46.42535500	40.84539500	45.14220400
N	45.99625100	39.18753700	46.66342800
H	41.69318500	44.35281000	45.50738600
H	44.59642700	45.36970300	45.84271900
H	43.53194700	45.09833800	47.22884100
H	43.16881900	42.73290500	46.57510400
H	44.12965200	42.93493700	45.11659500
H	46.20905300	43.18510700	46.46685900
H	45.27700200	43.29746300	47.94985800
H	44.97655600	41.01702400	48.01671900
H	46.32377000	41.78715600	44.79363900
H	46.85204200	40.08523100	44.56340100
H	45.85068900	38.84193000	47.60746400
H	46.50734400	38.57571000	46.01247200
C	42.94395500	40.46500100	35.22500900
C	44.08623400	40.29039300	36.24817500
C	44.71959000	38.92765500	36.41767600
O	45.93699900	38.75999300	36.34365900
O	43.88360200	37.95809400	36.72899800
H	42.19878500	39.66092900	35.31358900
H	44.90580500	40.99289900	36.04404300
H	43.69571500	40.53503500	37.25237400
C	41.40501200	35.44397200	35.83100800
C	41.03600400	35.09680800	37.26627100
O	39.97072500	35.40538500	37.76850000
C	40.70155600	36.71588700	35.36512500
H	42.50070700	35.56316300	35.76356000
H	41.02951200	37.57822100	35.96519700
H	40.91991900	36.92492300	34.30691400
H	39.61460500	36.61753000	35.49212800
N	41.99820300	34.42679600	37.97695000
C	41.80094500	34.17337300	39.38608300
C	41.94312200	35.39788700	40.30287900
O	41.33814000	35.44946200	41.36331400
H	42.88222000	34.21152600	37.53367000
H	40.78765800	33.78572500	39.55468700
H	42.53637300	33.43340700	39.72574600
N	42.79752900	36.36011700	39.89100100
C	43.11206800	37.48593300	40.74383500
C	44.38782400	38.23601000	40.31591700
C	44.73314900	39.35107200	41.29156800
O	45.54068100	37.39262600	40.19572000
H	43.31576300	36.21956400	39.03044100
H	43.17826500	37.14507100	41.78691500
H	44.18624400	38.67921400	39.32096300
H	45.38548100	36.81373700	39.41737700
H	43.87947300	40.03202600	41.42592500
H	45.00610000	38.93424400	42.27211100
H	45.58806400	39.93565700	40.92023400
C	38.77100800	34.70902600	43.65699600
C	40.18798000	34.06400900	43.77696900
C	40.40484400	32.90696800	42.79827900
C	41.86227500	32.47444000	42.63729800
N	42.40159800	31.85362700	43.85626800
C	43.67813800	31.51200900	44.06585500
N	44.62506400	31.80708500	43.13398600
N	44.04822000	30.82819300	45.14703900
H	37.99279400	33.92616900	43.63185600
H	40.95023200	34.83093100	43.58301100
H	40.34230400	33.71128400	44.81428200
H	39.77689100	32.03796200	43.07372300
H	40.07334000	33.23227500	41.80190800
H	41.94558400	31.74772500	41.80917700
H	42.46600700	33.35598900	42.38858600
H	41.75318600	31.69163100	44.61876000
H	44.54642900	32.80533700	42.84810500

H	45.59659400	31.59629900	43.44163700
H	43.40628100	30.71564900	45.92776800
H	45.02678000	31.02846400	45.45624700
C	41.02900200	25.29697300	40.27297300
C	41.20600500	26.91103300	40.03499700
C	42.34603000	27.20366800	39.05212600
C	42.54061500	28.65838200	38.60583000
N	43.19102400	29.51140600	39.60471300
C	43.79477200	30.68566800	39.32691400
N	43.76507700	31.17176300	38.07571600
N	44.43097200	31.37631300	40.26500300
H	42.00556700	24.83571900	40.48145900
H	40.25476100	27.32020400	39.65770600
H	41.39709500	27.38093100	41.01516200
H	43.30245000	26.82700300	39.45607200
H	42.16394900	26.62591800	38.13056800
H	43.18061900	28.65275200	37.70960400
H	41.56486900	29.09163300	38.31627000
H	43.17606900	29.21609400	40.57442400
H	43.18988800	30.73800500	37.36935200
H	44.32545300	31.99765700	37.81546400
H	44.44187600	31.10773300	41.25490000
H	44.69032400	32.36912800	40.07989000
C	46.45288600	30.40996800	48.86601700
C	46.77120000	31.68811200	49.65272200
O	46.80246200	31.74404900	50.86931800
C	44.94021400	30.25837800	48.68622400
O	44.39261000	31.33114700	47.92496500
H	46.89931600	30.50113300	47.86455800
H	44.73271000	29.28963600	48.18919400
H	44.43194600	30.24578000	49.66365600
H	45.08727300	31.59759800	47.28088300
N	46.90765800	32.78688700	48.83658300
C	46.99203100	34.15611800	49.30699000
C	45.89387500	35.06372400	48.70803800
O	45.98703100	36.28850800	48.74271300
C	48.34858200	34.80113600	49.01380000
O	48.53580100	35.08247300	47.64292200
H	46.82981300	32.62377000	47.83021300
H	46.85404500	34.11929400	50.40089000
H	48.40336800	35.76626900	49.53576300
H	49.13802600	34.14104800	49.41056100
H	48.33044900	34.31242900	47.08515800
N	44.86020100	34.40630900	48.13371100
C	43.75798200	35.09199400	47.49692800
C	43.65728000	34.81690400	45.99256800
C	44.91548700	35.30145500	45.26219500
C	44.81493300	35.33711600	43.75062600
N	45.42682300	36.36315300	43.13548400
O	44.20333100	34.47161100	43.10971700
H	44.80265500	33.39369800	48.23255800
H	43.92683200	36.16624200	47.66297900
H	43.51290000	33.74073800	45.81152100
H	42.76057700	35.32300200	45.59747600
H	45.20271500	36.29639900	45.63652600
H	45.75576900	34.62670100	45.49967700
H	45.50942100	36.34456000	42.11975300
H	45.98692300	37.03915200	43.65334600
C	51.82916800	33.65810500	49.30001000
C	51.60933900	33.83930100	47.78774800
O	51.44768900	32.63814300	47.05290000
H	52.81476100	33.22375800	49.52688000
H	50.75083900	34.51254200	47.62655100
H	52.49254400	34.35217900	47.36528100
H	50.51243400	32.37098200	47.02292500
C	49.79100000	39.12701300	48.44099500
C	49.52626800	38.42081200	47.09972000
C	50.60453600	38.67087300	46.06866300
C	50.64597800	39.90115400	45.39023500
C	51.60883300	37.74079700	45.76551600
C	51.63480100	40.19704100	44.45895600
C	52.61176900	38.01830300	44.82766500
C	52.64021600	39.25814000	44.16091500
O	53.57234300	39.60516200	43.26216200
H	50.13470200	40.16108200	48.27100100
H	49.38057800	37.34115200	47.25696300
H	48.57761300	38.79409900	46.68578400
H	49.87369400	40.64768700	45.60485700
H	51.60426400	36.76941500	46.26941800
H	51.65877300	41.15212700	43.93352400
H	53.38723300	37.28004400	44.60775800
H	54.17077400	38.84688900	42.97305900
C	54.54499700	40.14802500	38.94597100
C	53.37415300	39.74625700	39.84654100
O	52.19887200	40.52712100	39.59805200
H	55.34041900	39.39054100	39.04154400
H	53.14392700	38.67740600	39.66686200
H	53.66058200	39.81953000	40.90535600

H	51.78483300	40.75168300	40.44651300
C	57.82602600	39.04404700	43.41106600
C	56.94114000	39.29584000	42.15990100
C	56.15877800	38.06969300	41.65465800
O	56.67214600	37.41371600	40.71705400
O	55.03193800	37.78395700	42.17895900
H	58.00537800	37.96495600	43.54961500
H	56.22376600	40.10515400	42.36829400
H	57.57348300	39.61212300	41.31846300
C	54.90485100	31.33995700	42.21900900
C	53.65517100	32.15771500	41.93133300
C	52.37694000	31.33726900	41.98202600
O	52.43637300	30.09817300	41.84405800
O	51.26697800	31.97494100	42.07179200
H	54.85298000	30.36968000	41.70709600
H	53.55262100	33.03060600	42.59428700
H	53.68660800	32.57245600	40.90695800
C	50.15100700	26.19804800	46.21392800
C	50.19551900	27.61049400	46.79593700
O	49.39996100	28.46702200	45.98517600
H	49.13577300	25.77743300	46.29326800
H	49.82405300	27.59966400	47.83805000
H	51.24270200	27.96937500	46.83217100
H	49.22419100	29.32939500	46.41035000
C	45.55198000	26.43195600	42.70694900
C	45.25993200	27.02378200	44.09458700
C	45.21341200	25.93232100	45.16759500
C	46.26485800	28.11925800	44.46810700
H	44.78486800	25.70201700	42.40049400
H	44.25813600	27.49458100	44.04912700
H	46.19575300	25.43836000	45.26063200
H	44.96046800	26.35186600	46.15445800
H	44.46877500	25.15560200	44.92861800
H	47.28145500	27.71716000	44.60236900
H	46.31324200	28.91900300	43.71216900
H	45.99538800	28.59784700	45.42236500
C	46.96516600	28.39803600	39.70187400
C	48.43400200	28.15023400	39.35365700
C	49.27278900	27.95245500	40.61890300
C	49.15170000	29.13146700	41.57438300
C	49.83725400	28.91329300	42.90849400
N	49.80664900	30.19013700	43.64990000
H	46.78272900	29.42004800	40.06127300
H	48.52130500	27.25510900	38.71448000

H	48.83049400	28.99594300	38.77003000
H	48.94463000	27.02825700	41.12926600
H	50.33285500	27.79784600	40.35859500
H	49.57719000	30.02831300	41.09923000
H	48.09261000	29.35684000	41.77418600
H	49.32491500	28.15941300	43.51850500
H	50.88812600	28.63001000	42.77433300
H	50.40056200	30.89858000	43.15791700
H	48.83367900	30.59996500	43.68025500
H	50.08019000	30.08666500	44.63372100
C	45.80573600	28.83519900	36.10851200
C	46.76069000	29.60292000	35.16057300
C	48.01906700	30.18982600	35.83574600
C	47.63786800	31.28013400	36.78059200
O	47.32001000	32.41628400	36.25095800
O	47.57503300	31.06355000	38.01587200
H	44.81328700	29.30430800	36.15643600
H	47.11463600	28.93312300	34.36251600
H	46.23151700	30.42296800	34.64909400
H	48.66987300	30.64625900	35.07726500
H	48.58855000	29.41944800	36.37094300
H	47.71551100	32.05971400	38.78249400
C	52.34810000	24.35408200	37.72069600
C	52.87204800	25.63797800	38.39004300
C	52.07923200	26.85541100	37.88800500
C	52.56820600	28.22231300	38.36945000
N	51.91002100	29.28476000	37.61598300
C	51.41333600	30.41989400	38.09487100
N	51.39486000	30.70950100	39.39571400
N	50.88632100	31.32665200	37.24789600
H	52.27660200	24.48410500	36.62888100
H	52.80831000	25.55718100	39.48869200
H	53.93999100	25.77444100	38.14644100
H	52.11089900	26.86067900	36.78403500
H	51.01353000	26.74658200	38.15541200
H	52.38364200	28.35024200	39.44640200
H	53.66164600	28.30035500	38.23002400
H	51.86098300	29.20651400	36.57976300
H	51.80213200	30.13422600	40.14339900
H	51.03182000	31.60348000	39.70845300
H	51.00762400	31.25905700	36.22403800
H	50.65925400	32.22612800	37.66990200
C	50.01288000	30.57670200	31.79137600
C	50.40272900	29.55228000	32.84232000

C	50.97347900	30.11703400	34.14352300	C	46.93789300	35.64609700	35.28790000
O	50.81432400	31.36488800	34.40227900	N	47.59007900	36.64094400	36.19505600
O	51.50565500	29.30950500	34.92277500	H	46.02970500	38.16187800	31.71986500
H	49.25060700	31.27737700	32.16636300	H	47.23448600	36.11924200	31.04673000
H	49.53070800	28.94249100	33.14143100	H	48.79467000	36.92274000	31.17573900
H	51.13628500	28.82964300	32.45278500	H	48.46447700	35.38932600	33.04674300
C	51.92988000	33.85246400	31.53934100	H	48.65900100	37.04732200	33.58665300
C	51.30731000	35.13229600	32.09318400	H	46.15715500	37.18481400	33.99242200
C	51.10889400	35.08849000	33.57403000	H	45.98655500	35.53825800	33.38264700
C	51.17372800	36.08960000	34.51892000	H	46.00633900	35.34931300	35.78057400
N	50.69090300	33.94579200	34.24168600	H	47.58438100	34.75820800	35.23962200
C	50.49097200	34.23829500	35.52318800	H	46.91569400	37.43021800	36.39451900
N	50.76636200	35.53099300	35.71952800	H	47.91074400	36.21966200	37.09186300
H	52.08748500	33.92465000	30.45435100	H	44.35764000	37.13308300	37.04760200
H	51.92745400	36.01046500	31.85768500	C	49.69990100	40.63294000	33.03399200
H	50.32861900	35.31383000	31.61600200	C	50.50842700	40.22818400	34.26823900
H	50.67912200	32.91881300	33.95686600	C	49.67972800	39.34029600	35.18960600
H	51.45814000	37.13656900	34.41328000	O	51.65530100	39.45115700	33.92484800
H	50.17963500	33.54267200	36.28772800	H	48.66117700	40.85848000	33.32747500
H	50.66130100	35.95078300	36.66143900	H	50.81641700	41.12674600	34.83592000
C	54.69424100	32.85211200	34.18398100	H	52.28637400	40.00819500	33.45058700
C	54.56575800	32.58890800	35.74392200	H	48.76094700	39.84028900	35.52492100
C	53.99326300	33.75344700	36.56301900	H	49.39339700	38.43660300	34.62927000
C	53.91763200	33.42098800	38.06447700	H	50.27082400	39.04610300	36.06952000
N	53.32004800	34.43400900	38.92560500	P	47.64251500	32.01020800	45.37440300
C	54.01856900	35.30794300	39.70041700	C	49.00233400	33.99188600	43.09562100
N	55.29975100	35.56232200	39.45057600	C	49.73055800	35.03566800	42.19991500
N	53.40694900	35.91031100	40.72258400	C	48.86259900	34.44395700	44.57930000
H	55.30610700	33.75059500	33.99939500	C	50.06715300	36.33761000	42.93593600
H	53.93666600	31.69467400	35.89749300	C	48.90696600	36.77589800	43.78408100
H	55.56907100	32.33409200	36.12541800	C	48.38212400	35.86477300	44.62120600
H	54.60059400	34.66383100	36.41205500	O	47.90294700	33.66529700	45.30535400
H	52.98007100	34.00074600	36.20755800	O	46.29265100	31.97475900	46.13216300
H	53.32711800	32.50059600	38.19109700	O	48.83531800	31.37037500	46.07852900
H	54.92534900	33.18635100	38.44516700	O	47.42564400	31.51736800	43.89915600
H	52.31534800	34.35814500	39.08770800	O	47.72655200	33.75967600	42.54221500
H	55.68872100	35.26581400	38.56602500	O	50.89242300	34.53760100	41.56076200
H	55.85595200	36.28290100	40.03289500	C	48.26034700	38.10531700	43.58396600
H	52.51952700	35.53081700	41.07215300	O	47.29706000	38.44653900	44.33591700
H	53.95409900	36.58699300	41.29788800	O	48.62217000	38.80964000	42.59033900
C	47.10485000	38.22962000	31.48548800	H	47.43939200	32.90594600	42.94771700
C	47.79159100	36.87214200	31.63197000	H	51.06276800	33.58300500	41.79854300
C	47.96123400	36.36926200	33.06918200	H	50.94082500	36.15130400	43.57941300
C	46.68341300	36.22059000	33.89703400	H	50.37118300	37.11768500	42.22627900

H	48.97374500	35.27678200	41.42589800
H	49.59547200	33.06706800	43.09237400
H	49.85461400	34.34060300	45.06156800
H	47.56971700	36.10126200	45.30428800
P	45.45559100	34.50921400	38.43337400
C	48.13116400	34.12624100	39.02590700
C	47.96482700	32.98026600	39.77383600
C	49.50705800	34.64145400	38.58018900
O	47.16636300	34.84479500	38.49660700
O	45.00132200	35.90085300	37.96352600
O	45.00462300	34.01591600	39.77879100
O	45.38196100	33.43090000	37.31074300
O	50.43598400	33.81379000	38.56281200
O	49.53908000	35.84209900	38.18772600
H	48.86107100	32.60593100	40.27274000
H	47.02214400	32.88279500	40.31568200
H	42.74386700	45.00750800	44.23404300
H	42.06260400	46.07429500	45.48592800
H	43.31793600	40.49254200	34.19155600
H	42.44907900	41.41250400	35.46230100
H	47.18161700	38.59643600	30.45069000
H	50.03119200	41.51387200	32.47417500
H	49.66570600	39.78041700	32.33764800
H	47.52317800	39.00100800	32.14051800
H	40.57627100	24.82161500	39.39184400
H	40.38216400	25.17569300	41.14816600
H	38.50188300	35.38753900	44.47320300
H	38.70769800	35.26805600	42.71027500
H	51.35164700	24.07361800	38.09651600
H	53.04411500	23.52994900	37.90911100
H	50.85414500	25.50838200	46.69258100
H	50.41298400	26.23549100	45.14332600
H	55.80969600	31.87175300	41.88406500
H	55.02482700	31.15425800	43.29145700
H	54.97981000	41.11894200	39.20529800
H	54.22573000	40.18303900	37.89068200
H	58.81861000	39.49862900	43.32695200
H	57.34652500	39.41157700	44.33013600
H	51.73137400	34.63179600	49.79113600
H	51.05985600	32.99091200	49.72319900
H	48.89448300	39.18827800	49.06671800
H	50.58069800	38.61653200	49.01429400
H	46.89419300	29.49653100	49.27824800
H	42.84610600	34.83676700	48.04684500
H	55.20760700	32.00024100	33.71274900
H	53.74417900	32.98333500	33.65522900
H	49.63184300	30.10783100	30.87762900
H	51.29776300	32.97915900	31.73498600
H	50.89069700	31.16489200	31.48997800
H	52.89633200	33.69772100	32.03035400
H	48.73552800	44.26152800	38.59805300
H	47.36083600	43.57808900	39.46724500
H	48.09320300	42.96904800	41.09413100
H	47.58282300	43.46576300	42.73111700
H	41.15733300	34.61074600	35.16513800
H	42.31602200	38.23452500	40.67221000
H	45.68746200	27.81135900	35.73756900
H	46.20053900	28.78717200	37.12904100
H	46.52993400	25.92099500	42.69328600
H	45.59410100	27.23436000	41.96307100
H	46.67308000	27.69768100	40.48965000
H	46.29558000	28.22623600	38.84903300
H	48.41659200	37.06021800	35.75820300
H	46.50902800	32.92789600	36.77252700

Int1(7.6)

C	47.75502100	43.77604200	38.46501200
C	47.92114700	42.50198400	37.63036400
C	48.82862500	41.40739600	38.22917700
C	48.12032600	40.31999500	39.05529500
C	48.99698100	39.07598000	39.14952800
N	48.42756100	38.01845100	40.03914900
H	47.07587300	44.48842800	37.97092900
H	46.92902200	42.06760300	37.40682400
H	48.33594900	42.79639300	36.65097600
H	49.35563100	40.90757600	37.40272300
H	49.62953000	41.84881500	38.84398600
H	47.90345800	40.68357800	40.06988200
H	47.15749300	40.04902400	38.58110300
H	49.13009400	38.62315000	38.15550700
H	50.00072800	39.33539800	39.52119700
H	47.40039500	37.88949000	39.93846900
H	48.87969000	37.11688000	39.81643000
H	48.56547100	38.26872000	41.07546000
C	47.94697800	42.62094600	42.12197700
C	49.29321800	42.10653800	42.63278900
O	49.84740400	41.11867700	41.78513300
H	47.19102300	41.81820700	42.11872400

H	50.00731600	42.94625700	42.68095400	H	40.93723700	33.73671300	39.52490200
H	49.19005700	41.71754500	43.66152300	H	42.68367200	33.37233200	39.64359000
H	49.52069000	40.25622500	42.11372900	N	42.83543700	36.36287700	39.87996500
C	42.49295300	45.08396200	45.30404400	C	43.11209300	37.48618900	40.74436200
C	43.72497900	44.78702700	46.18801700	C	44.41552300	38.22830800	40.38529400
C	44.04255500	43.27867300	46.17989100	C	44.73334000	39.32546000	41.38982700
C	45.28792000	42.86651900	46.97900800	O	45.55241100	37.36437400	40.30852700
N	45.41463300	41.42318600	47.20426200	H	42.94130400	36.47309700	38.88070300
C	45.94727200	40.52505100	46.35236300	H	43.14371600	37.11259300	41.77590500
N	46.40732100	40.89127900	45.15532100	H	44.27062500	38.69598400	39.39155800
N	46.01964900	39.22857600	46.68390900	H	45.37723600	36.68935800	39.61938100
H	41.69285700	44.35312000	45.50822600	H	43.90272600	40.04419500	41.45645700
H	44.59640300	45.36786400	45.83919300	H	44.91038900	38.89860300	42.38710700
H	43.53266600	45.10710800	47.22647100	H	45.64098600	39.86911500	41.08853100
H	43.17671000	42.73436800	46.59490200	C	38.77100100	34.70900600	43.65703400
H	44.14189800	42.92685200	45.13706500	C	40.18798200	34.06400800	43.77694400
H	46.21113900	43.23259700	46.49900800	C	40.41038200	32.87054600	42.84327700
H	45.26596800	43.33580600	47.97415100	C	41.86972500	32.43064800	42.69638900
H	45.03501100	41.05409900	48.06799800	N	42.42503200	31.83369500	43.91982500
H	46.31480800	41.83936500	44.82197700	C	43.71116700	31.51406100	44.10911100
H	46.82281900	40.13413700	44.56398200	N	44.63653700	31.81127300	43.15975200
H	45.87726000	38.88761900	47.62967800	N	44.11075400	30.84053600	45.18699400
H	46.52377600	38.61996000	46.02486800	H	37.99239500	33.92623900	43.63663100
C	42.94482100	40.46550300	35.22576100	H	40.95177000	34.82196200	43.56156400
C	43.07578300	39.64622000	36.51687500	H	40.34719100	33.74301800	44.82373900
C	44.08266400	38.51945300	36.50803300	H	39.78029700	32.01316100	43.14903900
O	45.19320300	38.59470300	35.99977200	H	40.08148800	33.15130100	41.83153500
O	43.66645800	37.44837100	37.17740300	H	41.94724800	31.68780800	41.88114500
H	42.35830100	39.92988000	34.46498800	H	42.46787500	33.30794200	42.42428400
H	43.43213900	40.30302600	37.33307000	H	41.78612600	31.64528300	44.68424100
H	42.10795600	39.24794500	36.85076900	H	44.53280400	32.79831500	42.84596600
C	41.40493900	35.44397700	35.83097800	H	45.61940600	31.64410500	43.46210000
C	41.10696000	35.09263900	37.27812200	H	43.49908000	30.75280100	45.99607600
O	40.06165000	35.38515400	37.82916200	H	45.09268000	31.04419800	45.46657200
C	40.63238500	36.68244800	35.38332900	C	41.02843700	25.29707500	40.27279700
H	42.49084600	35.60248600	35.71902900	C	41.20603100	26.91086600	40.03479200
H	40.91682100	37.56168600	35.97975100	C	42.26668400	27.17015400	38.96226300
H	40.82060900	36.90411400	34.32202600	C	42.51462400	28.62563600	38.56697200
H	39.55403400	36.52898300	35.52710000	N	43.22871900	29.38598700	39.59400100
N	42.11776200	34.45772900	37.95560700	C	43.86052800	30.55407300	39.37238700
C	41.94764200	34.13203700	39.35266900	N	43.87903000	31.08038500	38.13869200
C	42.12668700	35.29957200	40.32871300	N	44.47101600	31.20337100	40.35847800
O	41.69296400	35.21576700	41.46425600	H	42.00574800	24.83482100	40.47393700
H	42.96153500	34.18432000	37.46789500	H	40.23550700	27.34002400	39.73817100

H	41.48731900	27.36751800	40.99976200
H	43.22843100	26.72139700	39.26709900
H	41.96775600	26.64308900	38.04119900
H	43.12994200	28.62459000	37.65333100
H	41.55765900	29.12367200	38.32459900
H	43.17857200	29.05834800	40.55213100
H	43.49935800	30.55534700	37.36420100
H	44.53810600	31.84921500	37.92443000
H	44.40714100	30.93553000	41.34517500
H	44.77325800	32.18740500	40.22652800
C	46.45287800	30.40997000	48.86604000
C	46.69886100	31.67365400	49.71117200
O	46.61965900	31.68291100	50.92636700
C	44.95317100	30.23868500	48.62779800
O	44.42278100	31.33616200	47.88926700
H	46.93584200	30.54037600	47.88600200
H	44.77641400	29.28866100	48.08448400
H	44.41102900	30.18272400	49.58497100
H	45.13827800	31.62807700	47.27801200
N	46.89967700	32.81013100	48.95585100
C	46.87177900	34.16168800	49.49603100
C	45.75478100	35.04269100	48.89936000
O	45.74667300	36.25818500	49.07458600
C	48.16880300	34.94268200	49.27020200
O	48.36635100	35.25841600	47.90598800
H	46.88651000	32.69202300	47.94013800
H	46.70067500	34.06033800	50.58022500
H	48.08679100	35.90235800	49.79592200
H	49.01117000	34.37694700	49.69937800
H	48.35207000	34.46107500	47.35293700
N	44.84738800	34.39329700	48.13810400
C	43.75801000	35.09193500	47.49695100
C	43.66869600	34.81997500	45.99674500
C	44.93500100	35.30824100	45.28739400
C	44.84313200	35.34603700	43.77756200
N	45.42847700	36.39449500	43.17399700
O	44.25629000	34.46942200	43.13215900
H	44.80813900	33.37674100	48.18041700
H	43.93241100	36.16279100	47.67505300
H	43.52511300	33.74513100	45.80830700
H	42.77728300	35.32817400	45.59271200
H	45.20981800	36.30545200	45.66630300
H	45.77536800	34.63928700	45.53888200
H	45.52700800	36.38975100	42.15974400

H	45.97381900	37.07461600	43.70072400
C	51.82910200	33.65806900	49.29998900
C	50.92911400	33.47868800	48.07732900
O	51.31335100	32.35440300	47.31597100
H	52.88100800	33.56672200	48.98072800
H	49.88798700	33.34788600	48.40566100
H	50.95342400	34.40869800	47.46953700
H	50.51222700	32.03675300	46.85498500
C	49.79097600	39.12702100	48.44101300
C	49.55415500	38.35996900	47.12857900
C	50.62208400	38.61856000	46.08971700
C	50.60801000	39.82216800	45.36402500
C	51.67141700	37.72698000	45.82669700
C	51.58668000	40.12823500	44.42571800
C	52.66436800	38.01411200	44.88051600
C	52.63631100	39.22739800	44.16692600
O	53.55314000	39.58554900	43.25740300
H	50.10277600	40.16385500	48.23012000
H	49.45292800	37.28341900	47.33082300
H	48.59050200	38.67622500	46.70111100
H	49.79830000	40.53713900	45.54541500
H	51.71353100	36.77857400	46.37126100
H	51.56884000	41.06008600	43.86107300
H	53.47427400	37.30498100	44.69146800
H	54.17213700	38.84200800	42.97395400
C	54.54497200	40.14800300	38.94595500
C	53.37527800	39.74319000	39.84616300
O	52.19567500	40.51865100	39.59817400
H	55.34184300	39.39237900	39.04139300
H	53.14865900	38.67364000	39.66729200
H	53.66055200	39.81824700	40.90494500
H	51.78873700	40.75250900	40.44733900
C	57.82600100	39.04389400	43.41090500
C	56.90688700	39.39725800	42.21381300
C	56.17349400	38.18016300	41.64096100
O	56.70799700	37.59142900	40.67265000
O	55.06931500	37.81978200	42.16887500
H	58.00040400	37.95676200	43.46291300
H	56.16567500	40.15302400	42.51577500
H	57.50556400	39.81299200	41.39132700
C	54.90509000	31.34008900	42.21896600
C	53.65760500	32.16709900	41.95200800
C	52.37437600	31.35542500	42.03351200
O	52.41762500	30.11486700	41.93996500

O	51.26863700	32.01023900	42.09874800
H	54.84406300	30.37066500	41.70663500
H	53.57542900	33.03988500	42.61845900
H	53.67479400	32.58757100	40.93018900
C	50.15086600	26.19799000	46.21410700
C	50.21984900	27.61463500	46.78172100
O	49.40629400	28.47200100	45.98713800
H	49.13299300	25.78856100	46.31545500
H	49.87673200	27.61504300	47.83331100
H	51.26899400	27.96858000	46.78616800
H	49.25038400	29.33236100	46.42043700
C	45.55198000	26.43195600	42.70694900
C	45.26459100	27.02786300	44.09401100
C	45.21415900	25.93928800	45.16970800
C	46.27513000	28.11932800	44.46530300
H	44.78180800	25.70436000	42.40315500
H	44.26509500	27.50350500	44.04877200
H	46.19403000	25.44054000	45.26264700
H	44.96446800	26.36245200	46.15581200
H	44.46526400	25.16598000	44.93341400
H	47.28845400	27.71131600	44.60564500
H	46.32985700	28.91524700	43.70480000
H	46.00492200	28.60361000	45.41627000
C	46.96495300	28.39879100	39.70126300
C	48.43534900	28.13462400	39.36004100
C	49.27052600	27.94528800	40.62618300
C	49.23668600	29.17888200	41.51656200
C	49.80062600	28.94562500	42.90233300
N	49.76737200	30.22673200	43.63081100
H	46.78721600	29.41897800	40.07251300
H	48.51086700	27.23048100	38.73364100
H	48.85675400	28.95696600	38.75726500
H	48.88103000	27.07386400	41.18354200
H	50.31461100	27.70202000	40.37037500
H	49.79957900	29.99435100	41.03727000
H	48.20081900	29.53623400	41.63434300
H	49.20813000	28.21898000	43.47153900
H	50.84405300	28.61176500	42.85454000
H	50.38580300	30.92426600	43.15377400
H	48.80474500	30.66017600	43.63953400
H	50.01076700	30.12891600	44.62332700
C	45.80755200	28.83320900	36.11239400
C	47.30429100	29.14915200	36.10456700
C	47.66794400	30.55135000	36.57485600

C	47.61749500	31.62689700	35.50809800
O	47.67936400	31.44014900	34.32226500
O	47.60822900	32.89371400	36.00799900
H	45.24181500	29.51425500	35.45402500
H	47.82907400	28.42112800	36.73744000
H	47.71355200	29.00519700	35.09506200
H	47.06358500	30.86349300	37.44285100
H	48.72014700	30.57150100	36.90920400
H	48.58367100	31.90789500	39.07636600
C	52.34797600	24.35396200	37.72108400
C	52.87193600	25.63799400	38.39002300
C	52.05741300	26.83799300	37.87751200
C	52.48521900	28.21298600	38.38061200
N	51.78330700	29.25617300	37.63862500
C	51.54137800	30.48467700	38.08050200
N	51.77198300	30.84665900	39.34600700
N	51.04076500	31.41447200	37.24668900
H	52.27420000	24.48396600	36.62968300
H	52.80750400	25.56035900	39.48876400
H	53.93764000	25.78378400	38.14360300
H	52.11238900	26.84905900	36.77474900
H	50.98968400	26.69150300	38.11734800
H	52.27700900	28.30760700	39.45864300
H	53.57716400	28.33923200	38.25988800
H	51.47748900	29.09810300	36.65234100
H	52.04258100	30.20395300	40.09561500
H	51.46180200	31.76358600	39.65321300
H	51.03388400	31.28947400	36.21195600
H	50.97392000	32.34945300	37.64108300
C	50.01101900	30.57602400	31.79200300
C	49.92504800	29.60266000	32.95677800
C	50.49986900	30.06504000	34.29145300
O	50.77013900	31.30441000	34.48080600
O	50.65303300	29.18997700	35.16812900
H	49.41709900	31.48126700	31.99030900
H	48.86999600	29.35636400	33.15942800
H	50.41822000	28.64661700	32.72068600
C	51.92997500	33.85245700	31.53919000
C	51.20970800	35.05891800	32.14296900
C	50.98685200	34.99794600	33.62251700
C	51.03910900	36.01191600	34.55361900
N	50.55256400	33.86460800	34.29940000
C	50.33317600	34.17637800	35.57270500
N	50.60898800	35.47303500	35.75851900

H	52.10092800	34.00472100	30.46421800
H	51.75736000	35.99098400	31.93496000
H	50.22476400	35.17291700	31.65686900
H	50.53863500	32.82470900	34.02920000
H	51.33314000	37.05434500	34.43475000
H	49.95480500	33.49429200	36.32053400
H	50.55970500	35.90613200	36.69480900
C	54.69357800	32.85089800	34.18372900
C	54.56661800	32.58932200	35.74410700
C	54.02950900	33.76075700	36.57241000
C	54.10532000	33.45790900	38.07803800
N	53.50341800	34.44097500	38.96353200
C	54.18019700	35.35257200	39.70816200
N	55.44462200	35.65646600	39.42530200
N	53.56567700	35.94075900	40.73497100
H	55.30948400	33.74644500	33.99896000
H	53.92333200	31.70710000	35.90505600
H	55.56916900	32.31605800	36.11578700
H	54.59429500	34.68351500	36.34933400
H	52.98049800	33.97000700	36.30206600
H	53.60838300	32.49361400	38.27010600
H	55.15599300	33.31229900	38.37644800
H	52.50021300	34.34933100	39.12612700
H	55.82023000	35.37807600	38.52927100
H	55.97006600	36.41073200	39.98653100
H	52.70231500	35.53664500	41.11264500
H	54.09803300	36.64153200	41.30120000
C	47.10500200	38.23004000	31.48470000
C	47.78020300	36.87402300	31.68554400
C	47.69576500	36.32787000	33.11393700
C	46.26878800	36.17609700	33.64152100
C	46.20134200	35.55222300	35.02389300
N	46.86837400	36.40946900	36.06004600
H	46.02035600	38.18321700	31.66800500
H	47.33767700	36.13605700	30.99331600
H	48.84361900	36.95338100	31.40500400
H	48.19924200	35.34786300	33.16651300
H	48.26744100	37.00005400	33.77763200
H	45.75342900	37.15010700	33.65770100
H	45.69080100	35.52770700	32.96183900
H	45.16378700	35.43563200	35.36483800
H	46.68207300	34.56631800	35.06214000
H	46.58383400	36.06893300	37.00982200
H	47.88912000	36.41984500	35.97911000

H	44.37160100	36.77168500	37.38252800
C	49.70002800	40.63286100	33.03395200
C	50.54903900	40.15889100	34.21590300
C	49.71042900	39.32947900	35.18244400
O	51.61520200	39.30501900	33.80076100
H	48.68606300	40.88940000	33.38380700
H	50.95600800	41.02742800	34.76818500
H	52.25185400	39.81977200	33.28785400
H	48.87414500	39.91790800	35.58367500
H	49.29584400	38.45691700	34.65295900
H	50.32959100	38.97812200	36.02133800
P	47.69837500	32.10827000	45.38027400
C	49.09171300	34.07284900	43.14048900
C	49.89380600	35.07923100	42.26601800
C	48.91729600	34.54247900	44.61198100
C	50.07425000	36.45765800	42.91498100
C	48.88856700	36.85419900	43.74814400
C	48.40020500	35.95035000	44.61440800
O	47.97664800	33.75707700	45.35243200
O	46.37706700	32.04594700	46.17973600
O	48.91109400	31.42564700	46.01722000
O	47.43936900	31.65747900	43.89543800
O	47.83317100	33.89473900	42.52475900
O	51.15339800	34.60924400	41.85371700
C	48.21392500	38.16960200	43.54762400
O	47.27362500	38.50845900	44.32837200
O	48.53401800	38.86502200	42.53447300
H	47.47732600	33.07019000	42.93672100
H	51.21900200	33.61413600	41.94740500
H	50.95778100	36.39170600	43.56912700
H	50.31963800	37.21588700	42.16085900
H	49.23098600	35.20797900	41.37805200
H	49.64622800	33.12478300	43.16243500
H	49.90953400	34.48290300	45.10230600
H	47.58704400	36.18094600	45.29927900
P	45.63623000	34.26034500	38.73825900
C	48.43140800	33.95774000	39.31637400
C	48.37532300	32.61162000	39.90513500
C	49.78136700	34.59075400	38.87380900
O	47.43976300	34.61191800	38.91900200
O	45.34953800	35.66593200	38.17018200
O	45.13386600	33.88476100	40.09435100
O	45.67003600	33.16080000	37.67391400
O	50.71943500	33.77054900	38.82180500

O	49.74212700	35.78039000	38.49692700
H	49.18676500	32.48035900	40.63452700
H	47.39905000	32.40261000	40.35111500
H	42.74297400	45.00683400	44.23389700
H	42.06266500	46.07433200	45.48587300
H	43.93713100	40.69414400	34.80943700
H	42.44832200	41.41229600	35.46155400
H	47.23872500	38.57993900	30.44936800
H	50.03113000	41.51390900	32.47419900
H	49.59554600	39.79915800	32.32219000
H	47.52283800	39.00073800	32.14105100
H	40.56957800	24.82571600	39.39260800
H	40.38260600	25.17562500	41.14848000
H	38.50188600	35.38755900	44.47318600
H	38.69571500	35.26622600	42.70939600
H	51.35233000	24.07413000	38.09909100
H	53.04428200	23.53001700	37.90878800
H	50.85425200	25.50839500	46.69244500
H	50.39627300	26.22029000	45.13917200
H	55.81006100	31.86827800	41.87959800
H	55.02464300	31.15415200	43.29146000
H	54.97980100	41.11894300	39.20530900
H	54.22515300	40.18359300	37.89090100
H	58.81858600	39.49868700	43.32698800
H	57.38202500	39.34609600	44.37071700
H	51.73140600	34.63181000	49.79111600
H	51.64532200	32.86478800	50.04221300
H	48.89448300	39.18827800	49.06672000
H	50.59590500	38.66566900	49.03433100
H	46.89419300	29.49651800	49.27821900
H	42.84608100	34.83681100	48.04682400
H	55.20301100	31.99663700	33.71227500
H	53.74419500	32.98447200	33.65548800
H	49.63336500	30.10832400	30.87674400
H	51.34130200	32.93416900	31.66082000
H	51.05301400	30.87498900	31.60114500
H	52.89635100	33.69753900	32.03027400
H	48.72637900	44.27941400	38.59906900
H	47.36075200	43.57808000	39.46720400
H	48.09321500	42.96919500	41.09418300
H	47.57584000	43.46311400	42.73052600
H	41.15736900	34.61074900	35.16512100
H	42.31591800	38.23438400	40.67190100
H	45.68652900	27.81243400	35.73489600
H	45.38526000	28.89128500	37.12616800
H	46.52822500	25.91785800	42.69169200
H	45.59407300	27.23434200	41.96304600
H	46.67288500	27.69770100	40.48959600
H	46.30458000	28.24028200	38.83765800
H	46.47161800	37.37768500	36.00409900
H	46.93880900	32.95355000	36.75200700

TS2(12.5)

C	47.75503500	43.77567400	38.46494700
C	47.93588500	42.49410800	37.64364700
C	48.83246800	41.40664100	38.27170100
C	48.10652000	40.32921700	39.09443000
C	48.98789700	39.09437300	39.24886700
N	48.39593800	38.07118800	40.16303300
H	47.06991500	44.47702700	37.96355800
H	46.94861100	42.05601100	37.40751300
H	48.36787900	42.77945000	36.66880800
H	49.37832800	40.89802700	37.46326400
H	49.62105000	41.85552800	38.89697500
H	47.85293000	40.71146400	40.09347300
H	47.16177800	40.04071800	38.59553300
H	49.14684000	38.60535200	38.27632500
H	49.98143500	39.37162700	39.63173500
H	47.35555800	37.99690000	40.09925600
H	48.79100100	37.14475000	39.95493400
H	48.57559900	38.30322100	41.20046600
C	47.94679800	42.62121400	42.12195800
C	49.27685400	42.08217300	42.65237000
O	49.81213600	41.05811100	41.83758000
H	47.17352600	41.83457600	42.12010800
H	50.00970100	42.90715900	42.69082400
H	49.15466300	41.71940200	43.68847700
H	49.54307000	40.20753200	42.24255300
C	42.49283900	45.08381600	45.30413200
C	43.72503400	44.78719600	46.18801300
C	44.03154800	43.27438000	46.16905300
C	45.25132700	42.82197200	46.98702200
N	45.33284700	41.37240900	47.19507500
C	45.90226700	40.46980300	46.36830100
N	46.44869200	40.83758900	45.20928600
N	45.92395000	39.16882300	46.68846400
H	41.69288800	44.35314400	45.50894700
H	44.59811600	45.36618900	45.84098700

H	43.53237400	45.10255100	47.22783800
H	43.14899800	42.73726000	46.55792200
H	44.14685900	42.93441400	45.12390800
H	46.19378100	43.16567300	46.52952100
H	45.22299400	43.28111600	47.98719200
H	44.86559300	40.99783800	48.01207500
H	46.37343700	41.78374200	44.86612200
H	46.91026200	40.07486600	44.65466300
H	45.75606400	38.81730400	47.62565200
H	46.43949200	38.54883900	46.04679100
C	42.94386700	40.46493700	35.22416200
C	43.06385400	39.66868100	36.53493500
C	44.11768200	38.58482100	36.62376100
O	45.29196600	38.74595000	36.29780400
O	43.67587100	37.46935100	37.17085700
H	42.36001000	39.92254900	34.46680700
H	43.35101600	40.36188400	37.34734700
H	42.09827300	39.23553400	36.82821200
C	41.40490400	35.44404200	35.83088900
C	41.15147500	35.05958300	37.27709400
O	40.09675000	35.28630000	37.84463400
C	40.58695200	36.66733200	35.42378100
H	42.48200200	35.63209400	35.69184300
H	40.87117700	37.54695900	36.02041600
H	40.73566500	36.90625800	34.35981700
H	39.51778400	36.48586000	35.60041700
N	42.20446200	34.47198300	37.93179700
C	42.06419600	34.10942900	39.32102300
C	42.31635700	35.22589800	40.33707600
O	42.06694700	35.02912800	41.51621800
H	43.06130500	34.24036600	37.43888300
H	41.04076000	33.75360000	39.50548700
H	42.76493100	33.30570500	39.57240400
N	42.84547600	36.37932700	39.86413400
C	43.11207700	37.48627600	40.74516300
C	44.39887500	38.30689100	40.48081100
C	44.57739200	39.37027300	41.55664700
O	45.59411900	37.54148800	40.44215700
H	42.85289800	36.52934100	38.86345800
H	43.12365800	37.08722900	41.76742300
H	44.27275000	38.81753400	39.50353800
H	45.51255300	36.93716400	39.66468400
H	43.68639000	40.01019000	41.64311800
H	44.76713300	38.89561400	42.53184000

H	45.44342600	40.00671700	41.31991500
C	38.77122600	34.70934700	43.65689900
C	40.18787100	34.06385500	43.77704000
C	40.32870500	32.82006200	42.88622900
C	41.73159700	32.21974400	42.76187700
N	42.20143300	31.57491500	44.00061700
C	43.43696100	31.12249300	44.22479500
N	44.41054700	31.30450000	43.28337100
N	43.75557600	30.44519300	45.33008800
H	37.99462900	33.92468100	43.64363700
H	40.96158500	34.78941700	43.49463400
H	40.37522100	33.78703100	44.83113200
H	39.61006500	32.04071200	43.20618600
H	40.03717000	33.09736200	41.86077800
H	41.72852700	31.45868000	41.96011400
H	42.42686200	33.01924100	42.48283400
H	41.53738900	31.49022100	44.76250700
H	44.45349300	32.32933900	43.06017500
H	45.34223600	31.00987300	43.60661900
H	43.11665600	30.44730500	46.12062200
H	44.74861600	30.60751600	45.60913900
C	41.02943100	25.29698100	40.27322700
C	41.20589900	26.91114400	40.03494300
C	42.40065000	27.21161300	39.11210800
C	42.64348700	28.66635600	38.66827500
N	43.32091600	29.50139600	39.66725000
C	43.92400600	30.68678500	39.41219500
N	44.00852800	31.15851200	38.16474000
N	44.44266000	31.39454400	40.41448100
H	42.00593700	24.83729300	40.48617900
H	40.27396600	27.31204100	39.60472000
H	41.34100000	27.38839600	41.02086800
H	43.33105700	26.82517300	39.56489100
H	42.26379000	26.63434600	38.18250400
H	43.28469900	28.63423000	37.77330400
H	41.68744700	29.13498700	38.36989500
H	43.24321600	29.23450100	40.64233600
H	43.71013300	30.60732000	37.37275000
H	44.54283300	32.02399300	37.96947700
H	44.33136000	31.10089900	41.39337900
H	44.78456800	32.36200600	40.28605000
C	46.45214400	30.40931300	48.86558200
C	46.81408000	31.69059000	49.61607600
O	46.92205600	31.78021000	50.82586200

C	44.92816300	30.27107200	48.75270900
O	44.34195000	31.34566800	48.02924300
H	46.85403900	30.48275700	47.84578100
H	44.69579900	29.30234900	48.26470700
H	44.46908600	30.25353700	49.75414900
H	44.96544600	31.56147800	47.29524900
N	46.89133600	32.75824700	48.75975700
C	47.07341400	34.14362200	49.12958300
C	46.00602600	35.05029700	48.47951600
O	46.18758700	36.25865000	48.33180400
C	48.46997300	34.63226700	48.74899600
O	48.72759800	34.43381300	47.37703700
H	46.82288700	32.54899900	47.76794100
H	46.95372600	34.20742100	50.22453300
H	48.53705100	35.71218100	48.93882900
H	49.20525000	34.11500000	49.38548400
H	48.72125000	33.47823600	47.16602500
N	44.87442100	34.41057900	48.10811300
C	43.75780600	35.09097500	47.49594500
C	43.58611900	34.79152000	46.00790300
C	44.74360500	35.34803000	45.18829300
C	44.70488700	34.93072900	43.73791300
N	45.28772600	35.72366300	42.82988300
O	44.15117900	33.87640100	43.40785800
H	44.78797400	33.41073800	48.29242500
H	43.92571500	36.16855500	47.64132300
H	43.53147200	33.70389300	45.84999100
H	42.62547500	35.20915700	45.66209000
H	44.81948500	36.44285200	45.28308400
H	45.67630600	34.91684300	45.58199800
H	45.34034200	35.37880500	41.86938100
H	45.80607600	36.54981600	43.09869400
C	51.82741000	33.65799000	49.30058700
C	51.79552600	33.80819000	47.77056800
O	51.53631100	32.59831200	47.07571000
H	52.74854800	33.16348200	49.64598800
H	51.05458100	34.57537300	47.49302400
H	52.77935600	34.16828800	47.42230300
H	50.57881400	32.45476800	47.00430200
C	49.79107500	39.12693700	48.44104900
C	49.49944300	38.41687600	47.10560800
C	50.53835600	38.67935400	46.03846900
C	50.64759300	39.96316300	45.47572200
C	51.43636900	37.70359800	45.58559000

C	51.61668900	40.27135400	44.52931500
C	52.41832900	37.99363900	44.62914400
C	52.53107100	39.29171500	44.09773900
O	53.46384700	39.65867000	43.20813600
H	50.13176400	40.16005100	48.26385700
H	49.38255000	37.33295700	47.26289300
H	48.52890000	38.76620300	46.72416200
H	49.95264000	40.74496200	45.80011500
H	51.37434100	36.69134500	45.99609100
H	51.70240100	41.27202700	44.10385800
H	53.12273200	37.22313200	44.30535100
H	54.06972400	38.91063700	42.91401800
C	54.54499500	40.14804500	38.94601800
C	53.36726400	39.75364300	39.83846900
O	52.20400100	40.55468800	39.60223900
H	55.33775200	39.38978200	39.05034100
H	53.12237900	38.68975700	39.64772100
H	53.65459600	39.81356000	40.89624500
H	51.78756400	40.75405600	40.45577300
C	57.82592200	39.04403800	43.41112600
C	56.85572600	39.45566500	42.27544000
C	56.13573800	38.25270700	41.65797800
O	56.70681000	37.67377700	40.70452900
O	55.01025300	37.89128700	42.13797200
H	58.00119800	37.95580500	43.39641000
H	56.10946000	40.17167100	42.65088400
H	57.41396200	39.93545800	41.45895600
C	54.90462900	31.34018400	42.21933200
C	54.10163300	32.60290100	41.88093100
C	52.56809200	32.50504100	41.86963800
O	52.00600200	31.45952100	42.26571400
O	51.95834200	33.51291300	41.37112100
H	54.43671200	30.44660300	41.77757400
H	54.36793000	33.43923800	42.54934400
H	54.37568300	32.95828800	40.87543500
C	50.15108300	26.19822800	46.21365600
C	50.25643000	27.60204000	46.81196000
O	49.56265100	28.54328400	45.98959500
H	49.12952000	25.79793700	46.30365300
H	49.83841700	27.60577900	47.83437800
H	51.32084100	27.89181200	46.89906500
H	49.27794800	29.31583600	46.50735600
C	45.55198000	26.43195600	42.70694900
C	45.33982300	27.01266400	44.11235600

C	45.21283900	25.90239300	45.15932500	N	51.84499800	29.29191300	37.70436300
C	46.45116600	27.99706500	44.48557300	C	51.64801400	30.54065000	38.12635500
H	44.74233600	25.73893900	42.42399700	N	51.87159100	30.91958500	39.39982900
H	44.38657600	27.57574100	44.10214900	N	51.21290500	31.46766900	37.26954900
H	46.14391600	25.31234600	45.21573300	H	52.27628500	24.48403500	36.62859700
H	45.02423600	26.31907700	46.16172400	H	52.81123600	25.55208300	39.48862200
H	44.39099500	25.20751000	44.92154800	H	53.93981000	25.77648700	38.14457300
H	47.42702000	27.49202600	44.57141000	H	52.12831500	26.89150900	36.79411700
H	46.56124600	28.81097200	43.75376300	H	51.00616300	26.73104000	38.13778700
H	46.26145100	28.47354400	45.46013400	H	52.32448800	28.29873600	39.50236200
C	46.96474400	28.39917600	39.70089900	H	53.62659500	28.32785400	38.30173600
C	48.45675800	28.26551700	39.38749100	H	51.60842700	29.15624400	36.69594100
C	49.32183000	28.14990100	40.64571700	H	52.08793800	30.27872900	40.15154700
C	49.28103000	29.36766400	41.56845200	H	51.66673700	31.87316400	39.68978800
C	50.00965300	29.09471100	42.87690900	H	51.11394800	31.29371500	36.25268200
N	50.02824000	30.29465500	43.74033000	H	51.02657000	32.40418000	37.63770700
H	46.70200000	29.41009100	40.05305000	C	50.01067700	30.57623900	31.79184300
H	48.62084500	27.36745900	38.76869100	C	50.14508100	29.55710300	32.91535800
H	48.79974000	29.11640900	38.77215100	C	50.69329800	30.07270500	34.24755800
H	48.99391600	27.26029100	41.21403700	O	50.71683700	31.33552600	34.46398400
H	50.36702200	27.94823700	40.35617400	O	51.03603700	29.21636100	35.08591800
H	49.73642600	30.24035800	41.07177900	H	49.32906700	31.39659400	32.06633100
H	48.24129000	29.64477100	41.80389500	H	49.16356700	29.10952500	33.15224800
H	49.51424700	28.29454200	43.44246100	H	50.78086000	28.70993500	32.61451600
H	51.05300000	28.79382200	42.69606400	C	51.92993100	33.85217000	31.53926900
H	50.70988900	30.98062000	43.34261600	C	51.29123000	35.13622200	32.04026000
H	49.04074700	30.69897300	43.81010000	C	51.02948500	35.08247200	33.50395400
H	50.28538200	30.03101100	44.70068400	C	51.07728600	36.06734500	34.46106600
C	45.80686500	28.83322200	36.11271600	N	50.55839800	33.94035000	34.13831900
C	47.14258000	29.28095700	35.48605800	C	50.30499900	34.22534400	35.41260500
C	47.74150800	30.58095800	35.99377700	N	50.60853900	35.50593300	35.63329600
C	46.83557000	31.76627800	35.86197000	H	52.09938300	33.89019000	30.45330700
O	45.70081500	31.74941600	35.44088500	H	51.92283500	36.01231700	31.82966400
O	47.39578400	32.90061100	36.33806700	H	50.33486000	35.31140600	31.51661400
H	44.97432400	29.45786500	35.76705300	H	50.55451600	32.92246200	33.85854700
H	47.89880700	28.49374000	35.63771800	H	51.38851500	37.10779100	34.37627000
H	46.99931800	29.35622800	34.39499200	H	49.90588700	33.52292400	36.13145100
H	48.01625100	30.50733300	37.06095200	H	50.46177400	35.92330800	36.58073900
H	48.68397100	30.82912200	35.48213700	C	54.69428600	32.85287600	34.18420700
H	47.50314500	32.17168700	38.77377300	C	54.56559000	32.58862200	35.74384900
C	52.34810900	24.35428200	37.72025500	C	54.00235600	33.76916100	36.53503000
C	52.87259100	25.63780100	38.38975700	C	54.16990700	33.55626300	38.04197600
C	52.07578000	26.85992600	37.89649700	N	53.53604100	34.54842100	38.89063100
C	52.53204900	28.22282900	38.42208300	C	54.19221100	35.46661700	39.64065300

N	55.40051900	35.89662700	39.29117100
N	53.59750200	35.92912000	40.74284300
H	55.30233500	33.75490400	34.00571100
H	53.93785400	31.69736100	35.91225400
H	55.57308800	32.34224000	36.12120700
H	54.50581200	34.70670100	36.23823200
H	52.93223000	33.90839600	36.31122500
H	53.75667000	32.57015300	38.31097400
H	55.24141400	33.51233500	38.29540600
H	52.56058300	34.38458000	39.12879700
H	55.71857500	35.72904700	38.34604700
H	55.94630300	36.59028900	39.91009400
H	52.90167700	35.30861400	41.16534500
H	54.08929200	36.65932400	41.30862000
C	47.10441400	38.22935200	31.48620500
C	47.73593800	36.85854700	31.72749800
C	47.86342500	36.44904500	33.19805200
C	46.56516300	36.37614000	34.00608000
C	46.79304800	35.88042100	35.43042600
N	47.38366300	36.93150600	36.32279500
H	46.01815200	38.21079400	31.67259500
H	47.16424600	36.08767500	31.18111200
H	48.74882500	36.84581000	31.29132900
H	48.35027900	35.46208100	33.25119800
H	48.56236300	37.14831000	33.68557400
H	46.04636400	37.34875900	34.03106600
H	45.87171500	35.66691100	33.52457400
H	45.86311000	35.56746100	35.91717400
H	47.47444000	35.02154300	35.43961900
H	47.76768700	36.51876100	37.19747200
H	48.16478700	37.41302600	35.86449900
H	46.63897000	37.64694000	36.52378600
C	49.69971500	40.63318300	33.03419000
C	50.55975800	40.22910100	34.23533800
C	49.76169300	39.39751300	35.23273200
O	51.66162000	39.40950400	33.84913000
H	48.67527600	40.87175500	33.36449900
H	50.92657900	41.13235300	34.75915600
H	52.27229000	39.93200700	33.31296900
H	48.88959000	39.95293500	35.60399300
H	49.41095700	38.48615700	34.72266500
H	50.39319800	39.10880000	36.08584500
P	47.41233000	31.91383600	45.32949100
C	48.62828600	33.82427500	42.87801200

C	48.96228100	35.01370100	41.98257800
C	48.38140200	34.25268700	44.38056400
C	49.69526200	36.11365500	42.74774100
C	48.70570500	36.63219900	43.75519000
C	48.09269500	35.72232900	44.53184400
O	47.29977700	33.52390300	44.93357200
O	46.05936800	31.64962100	46.02050100
O	48.66143200	31.73486900	46.20213300
O	47.49407300	31.15508900	43.95789500
O	47.52996500	33.16328000	42.30357400
O	49.65195700	34.60636100	40.81762200
C	48.22911000	38.05055000	43.66331000
O	47.34599300	38.45688500	44.46593400
O	48.65844700	38.75529700	42.69555200
H	47.39048600	32.33702500	42.84016900
H	50.47806500	34.06371300	41.04421700
H	50.59064600	35.69989400	43.24054300
H	50.04595400	36.90437800	42.07641200
H	47.99751300	35.41942700	41.63347100
H	49.51833400	33.16569100	42.87845900
H	49.30590300	34.02192300	44.94167100
H	47.36639200	36.01532900	45.28694400
P	45.61243000	34.60584600	38.85019200
C	48.41752800	34.04236200	39.04204800
C	48.28015300	32.62214800	39.40637700
C	49.67715400	34.69260100	38.43611600
O	47.41254400	34.81052600	38.95173400
O	45.41147200	36.04285200	38.33185300
O	45.17895100	34.20713900	40.22915000
O	45.40402700	33.56962400	37.74210000
O	50.69514500	33.98774200	38.33346000
O	49.51493100	35.87312900	38.01401400
H	49.22612300	32.08662700	39.29616200
H	47.93325200	32.59378400	40.46087900
H	42.74264800	45.00625500	44.23404200
H	42.06272600	46.07437000	45.48582200
H	43.93872100	40.69468400	34.81348300
H	42.44933900	41.41247800	35.46294300
H	47.24643700	38.55481100	30.44470700
H	50.03137000	41.51373500	32.47407200
H	49.63403700	39.78270600	32.33847000
H	47.52357700	39.00132400	32.13977200
H	40.58118100	24.81805600	39.39188100
H	40.38189900	25.17567700	41.14796800

H	38.50175400	35.38740800	44.47326800
H	38.68617200	35.26254500	42.70782900
H	51.35150100	24.07495200	38.09617900
H	53.04376100	23.52979000	37.90974200
H	50.85407000	25.50841300	46.69273300
H	50.40861800	26.23178700	45.14191400
H	55.92101000	31.42657700	41.80369400
H	55.02544500	31.15379900	43.29130900
H	54.97983100	41.11894500	39.20524600
H	54.23457100	40.18237200	37.88793500
H	58.81861000	39.49860600	43.32685300
H	57.41762700	39.29008200	44.40257400
H	51.73276600	34.63213400	49.79073700
H	50.97876600	33.03859100	49.63370400
H	48.89443200	39.18834400	49.06664200
H	50.58494700	38.61680000	49.00838000
H	46.89481000	29.49690200	49.27840700
H	42.84649600	34.83757900	48.04786300
H	55.21139800	32.00625700	33.70666100
H	53.74423800	32.98246300	33.65489300
H	49.63358800	30.10816400	30.87673300
H	51.29836600	32.97893900	31.74185500
H	50.98941100	31.01445400	31.54716200
H	52.89631200	33.69834400	32.03057900
H	48.72118400	44.28915800	38.59844200
H	47.36073200	43.57881900	39.46737000
H	48.09328600	42.96852300	41.09397900
H	47.58830100	43.47106900	42.72775900
H	41.15738100	34.61071000	35.16516500
H	42.31590200	38.23431100	40.67129100
H	45.68708900	27.81241600	35.73476700
H	45.83672300	28.82696000	37.20845200
H	46.50380500	25.87627300	42.64875500
H	45.59395600	27.23426800	41.96293600
H	46.67325100	27.69779300	40.48981200
H	46.34327500	28.18070400	38.82413400
H	46.60473000	33.39279500	36.72011500
H	44.41858300	36.84902100	37.49724200

Int2(-6.1)

C	47.75496400	43.77598800	38.46495700
C	47.87924600	42.49524400	37.62985100
C	48.76925600	41.37404800	38.21150100
C	48.05040300	40.28320400	39.02700400
C	48.90917900	39.02265800	39.09618200
N	48.31462700	37.94526700	39.94788900
H	47.08960700	44.50526000	37.97702600
H	46.87291700	42.08699700	37.42265600
H	48.28501600	42.77758700	36.64324300
H	49.27878000	40.87688200	37.37269100
H	49.58401700	41.79410200	38.82348100
H	47.84705000	40.63419600	40.04919800
H	47.08283400	40.03184900	38.55347600
H	49.04144600	38.59351700	38.09296400
H	49.91021700	39.26039900	39.48611000
H	47.27579700	37.86679000	39.86875100
H	48.69484000	37.02730800	39.66560400
H	48.48459900	38.11586500	40.98228300
C	47.94694000	42.62115800	42.12203700
C	49.24398700	41.98219100	42.62275400
O	49.70412600	40.94320900	41.78294300
H	47.11768900	41.89310100	42.12495100
H	50.03014200	42.75605400	42.66762500
H	49.11062000	41.60987900	43.65430900
H	49.37295100	40.09772400	42.15409400
C	42.49305900	45.08408600	45.30397700
C	43.72502800	44.78694400	46.18794600
C	44.03659000	43.27558800	46.15946900
C	45.26658800	42.82442100	46.96227700
N	45.35165700	41.37487900	47.16814800
C	45.91029900	40.47177000	46.33465500
N	46.44872700	40.83846600	45.17142200
N	45.93000100	39.17052600	46.65331400
H	41.69336300	44.35269000	45.50730500
H	44.59632500	45.37076200	45.84446800
H	43.53077000	45.09568100	47.22943900
H	43.15992000	42.73346500	46.55472800
H	44.14275200	42.94082200	45.11171400
H	46.20297000	43.16915100	46.49333900
H	45.25024800	43.28277700	47.96311500
H	44.88685100	40.99929100	47.98605900
H	46.37091400	41.78390200	44.82655900
H	46.89946300	40.07437500	44.61132700
H	45.76960400	38.82116400	47.59230600
H	46.43810000	38.54946900	46.00494500
C	42.94375000	40.46484400	35.22479600
C	43.12651500	39.61033200	36.48733600
C	44.13965800	38.48057200	36.39067200

O	45.10799800	38.45789500	35.66683000
O	43.88214000	37.49390100	37.27605300
H	42.34439200	39.93520500	34.46890200
H	43.50243100	40.23791400	37.31755900
H	42.17385200	39.19373600	36.84680500
C	41.40498500	35.44403300	35.83099200
C	41.19036300	34.96033300	37.25473700
O	40.09367500	34.96322900	37.78614600
C	40.52589400	36.64690400	35.49960200
H	42.47097400	35.67356400	35.66562600
H	40.80058000	37.51768300	36.11486700
H	40.62092300	36.92946900	34.44048400
H	39.47365500	36.41250300	35.71240500
N	42.30711500	34.49792200	37.90434300
C	42.21814000	34.08727700	39.28407500
C	42.38288300	35.19778900	40.31827300
O	42.12795500	34.97781000	41.49279100
H	43.22399600	34.60023100	37.48468300
H	41.23224000	33.63579300	39.45460600
H	42.99710900	33.35164800	39.50940700
N	42.85334800	36.38089500	39.85790900
C	43.11192000	37.48591800	40.74399000
C	44.42031500	38.24684400	40.42690500
C	44.77138500	39.22813900	41.53546900
O	45.54290700	37.39067500	40.24433000
H	42.90674000	36.53521600	38.85802400
H	43.13399400	37.08429900	41.76508600
H	44.25687400	38.81416900	39.49015600
H	45.37729900	36.76596200	39.50594500
H	43.94254100	39.92990200	41.71481200
H	44.98796400	38.69608600	42.47426400
H	45.66691800	39.80845500	41.26732100
C	38.77100200	34.70898300	43.65706900
C	40.18797300	34.06401600	43.77692400
C	40.30984600	32.80913700	42.89968600
C	41.69671200	32.17164900	42.80515100
N	42.12702400	31.54029500	44.06617200
C	43.34724200	31.06851500	44.32543900
N	44.34720000	31.22949100	43.41192700
N	43.62582700	30.38922100	45.44227000
H	37.99532400	33.92349700	43.64687500
H	40.96328700	34.78135100	43.47678500
H	40.38334400	33.79968300	44.83260900
H	39.56683800	32.05206500	43.21796000
H	40.03987000	33.08338000	41.86748300
H	41.68486000	31.39393700	42.01940800
H	42.41723400	32.94641400	42.52021300
H	41.43663300	31.46038300	44.80491700
H	44.40740200	32.25245500	43.17477600
H	45.26251500	30.92649100	43.76528300
H	42.97410100	30.41095500	46.22106900
H	44.61717500	30.51218900	45.72862600
C	41.02948600	25.29697900	40.27318600
C	41.20607700	26.91113100	40.03525400
C	42.40817600	27.22299900	39.13232800
C	42.64275700	28.68917200	38.73203500
N	43.28064900	29.50659000	39.76954100
C	43.96613800	30.65258100	39.54641300
N	44.13895700	31.11245400	38.30006100
N	44.47803600	31.34006400	40.56019400
H	42.00598600	24.83754300	40.48684000
H	40.27767100	27.30999900	39.59553500
H	41.32614300	27.38640600	41.02451800
H	43.33508200	26.83255300	39.58816200
H	42.28220900	26.66649800	38.18850700
H	43.31057200	28.68602200	37.85758100
H	41.68926700	29.15140400	38.41586600
H	43.09005100	29.27655600	40.73855900
H	43.80735700	30.58876300	37.50350900
H	44.84206200	31.85152900	38.15027800
H	44.33817900	31.03897800	41.53182800
H	44.84764800	32.30432300	40.43652700
C	46.45333700	30.41015200	48.86575200
C	46.85016100	31.68417700	49.60890000
O	47.02742000	31.76469800	50.81109500
C	44.92471500	30.28464000	48.79921900
O	44.32148900	31.36176000	48.09471800
H	46.83117100	30.47504600	47.83857500
H	44.67091700	29.31667500	48.31992300
H	44.49694200	30.26941900	49.81444000
H	44.91826900	31.55868400	47.33361300
N	46.87626900	32.75988000	48.76031900
C	47.07094900	34.14362700	49.12791800
C	46.01861300	35.04821800	48.45272500
O	46.21693900	36.24851200	48.26807200
C	48.48258600	34.61005500	48.77703300
O	48.78880700	34.35794500	47.42440900
H	46.75564600	32.55858600	47.77319300

H	46.93157800	34.21662400	50.22018100
H	48.55281500	35.69568000	48.92995900
H	49.18970300	34.11075400	49.45879000
H	48.71853300	33.40055900	47.23688600
N	44.87518700	34.41336900	48.10847000
C	43.75801900	35.09180400	47.49707600
C	43.56792900	34.78393500	46.01191300
C	44.71058900	35.32900900	45.16510700
C	44.65983100	34.86855900	43.72717800
N	45.22671900	35.63140400	42.78523000
O	44.11006600	33.79886900	43.44193700
H	44.77568100	33.42099400	48.32503200
H	43.92428100	36.17054800	47.63588300
H	43.50852400	33.69520700	45.86364300
H	42.60181200	35.19939100	45.67896100
H	44.78261000	36.42618200	45.23067600
H	45.64716800	34.91074000	45.56103800
H	45.28670700	35.23937800	41.83952100
H	45.75927300	36.45608200	43.02827200
C	51.82921500	33.65806200	49.29992800
C	51.83577800	33.82450300	47.77132800
O	51.56107800	32.63116500	47.05180300
H	52.73631800	33.15234800	49.66537700
H	51.11900500	34.61229000	47.48837100
H	52.83555300	34.16017000	47.44603000
H	50.60366800	32.47287400	47.04443500
C	49.79100700	39.12700100	48.44100700
C	49.50146100	38.39771400	47.11380500
C	50.52994900	38.66279600	46.03684700
C	50.62683100	39.94663600	45.47133300
C	51.43028800	37.69241100	45.57764800
C	51.58995700	40.26118500	44.52140300
C	52.40723900	37.98959100	44.61708400
C	52.51195100	39.28943700	44.08917500
O	53.44350400	39.66892000	43.20301700
H	50.12223800	40.16073000	48.25062600
H	49.40123100	37.31410200	47.28402900
H	48.52330000	38.72757600	46.73402600
H	49.92783900	40.72381500	45.79762000
H	51.37703300	36.67991700	45.98891400
H	51.66737900	41.26254200	44.09618000
H	53.11600500	37.22390600	44.29060100
H	54.05500200	38.93262700	42.90232800
C	54.54496700	40.14802800	38.94600000
C	53.35056700	39.76733400	39.82185900
O	52.20182700	40.57846200	39.56272900
H	55.33281000	39.38704000	39.06542800
H	53.10304900	38.70396700	39.63171200
H	53.62532400	39.83286400	40.88317400
H	51.72404900	40.70825100	40.39734700
C	57.82599200	39.04392900	43.41099200
C	56.85912600	39.49167000	42.28782600
C	56.15690800	38.29939300	41.64252200
O	56.75004700	37.71977200	40.70420400
O	55.02030800	37.93661700	42.09753400
H	57.99635000	37.95588900	43.36921600
H	56.10683100	40.19093800	42.68123500
H	57.41796000	39.99791500	41.48795000
C	54.90516600	31.33997000	42.21891600
C	54.13412600	32.62585500	41.90038300
C	52.62041300	32.56757400	41.94116200
O	51.94752000	31.58314200	42.20245100
O	52.08081100	33.73178800	41.57481100
H	54.41413800	30.46339600	41.76972500
H	54.44504100	33.46804500	42.54117100
H	54.36316100	32.96748300	40.87757600
C	50.15092200	26.19795200	46.21402700
C	50.19843400	27.57632300	46.87618400
O	49.50870100	28.53853000	46.07227300
H	49.13803500	25.76857100	46.24906600
H	49.74257900	27.52593800	47.88054100
H	51.25047100	27.88933600	47.01490800
H	49.20165500	29.29348700	46.60570600
C	45.55198000	26.43195600	42.70694900
C	45.30566600	27.00738700	44.10843400
C	45.16562700	25.89558300	45.15189300
C	46.40156600	27.99988400	44.50360200
H	44.75848500	25.72661900	42.40934100
H	44.34859500	27.56333400	44.07930200
H	46.09860600	25.31026000	45.22454200
H	44.95627700	26.31009500	46.15107900
H	44.35220500	25.19658300	44.89825600
H	47.37700900	27.49969700	44.62020600
H	46.52727800	28.80561600	43.76570500
H	46.18469300	28.48588500	45.46765500
C	46.96512300	28.39862000	39.70150500
C	48.46219800	28.24052600	39.41202000
C	49.30311000	28.09033600	40.68322100

C	49.27263500	29.30523100	41.61041300
C	49.97357500	29.01933700	42.92973500
N	49.92938600	30.18245800	43.84272000
H	46.71567100	29.41236400	40.05409100
H	48.61925300	27.34628300	38.78639400
H	48.83382400	29.09021700	38.81673400
H	48.94581700	27.20590400	41.24178100
H	50.34877300	27.86638500	40.40905200
H	49.74268500	30.17164600	41.11790600
H	48.23398500	29.60014100	41.82799700
H	49.48056800	28.19114900	43.45651800
H	51.02711100	28.73924700	42.77268600
H	50.59283700	30.90955000	43.54403600
H	48.91950300	30.59174900	43.88824500
H	50.13222800	29.86781600	44.80577300
C	45.80665100	28.83370300	36.11178100
C	47.30432300	29.15166500	36.00271200
C	47.77271100	30.47392200	36.59607100
C	47.61719400	31.73076700	35.74551700
O	47.30850700	31.72159900	34.56613800
O	47.87441700	32.83658200	36.42752700
H	45.19510200	29.51792200	35.50070400
H	47.86773300	28.34588500	36.49818200
H	47.60477500	29.10759300	34.94697900
H	47.29328500	30.66156400	37.57094600
H	48.85215700	30.41527000	36.80692000
H	48.48382900	32.16470200	38.68474600
C	52.34811700	24.35404000	37.72084800
C	52.87196900	25.63800900	38.38999400
C	52.06737900	26.84695000	37.86635300
C	52.49356200	28.22634800	38.36730400
N	51.78275500	29.27712500	37.64113400
C	51.70357500	30.55378200	38.01428500
N	52.04416700	30.95499200	39.26870500
N	51.31456700	31.49407100	37.16224900
H	52.27643600	24.48415300	36.62951300
H	52.79687100	25.56343200	39.48866200
H	53.94056600	25.77767300	38.15251400
H	52.13261000	26.85602100	36.76447800
H	50.99670100	26.70630600	38.09599000
H	52.28734500	28.30809400	39.44867200
H	53.58539600	28.35461200	38.24402100
H	51.43017100	29.11153300	36.66689900
H	52.04433300	30.30648400	40.04550000
H	51.79816200	31.91497900	39.49425300
H	51.15423500	31.32631200	36.13987000
H	51.15116400	32.42863000	37.55156100
C	50.01110800	30.57591400	31.79191300
C	49.72625000	29.69743300	33.00698000
C	50.42319700	30.09539500	34.30554400
O	50.72875000	31.32445600	34.48854500
O	50.62362200	29.19997200	35.15573000
H	49.53525500	31.56297800	31.89523200
H	48.64571300	29.72523100	33.22927100
H	49.98181900	28.64453500	32.81607200
C	51.92995700	33.85189800	31.53912600
C	51.25660700	35.11369100	32.06049700
C	51.00849200	35.06552500	33.52939600
C	51.05154400	36.06213100	34.47677800
N	50.57593200	33.92019400	34.18433400
C	50.34961400	34.21010400	35.46275700
N	50.62189000	35.49981500	35.66644100
H	52.10278200	33.91840400	30.45502900
H	51.85646900	36.01032600	31.84268600
H	50.29121900	35.25827300	31.54458200
H	50.54681400	32.90197700	33.90388400
H	51.34059700	37.10719900	34.37515400
H	49.94523900	33.51623000	36.18923500
H	50.47411700	35.89627300	36.62580200
C	54.69402500	32.85205500	34.18401800
C	54.56593100	32.58900800	35.74403500
C	54.09000200	33.79683800	36.54618000
C	54.27026100	33.55316800	38.04557600
N	53.65864100	34.53863000	38.91395100
C	54.31042100	35.49865100	39.60676400
N	55.57653900	35.80939500	39.34235500
N	53.66610400	36.12711800	40.59443600
H	55.30834000	33.74901000	34.00236400
H	53.88588800	31.73866500	35.91570700
H	55.56100400	32.27909400	36.10839800
H	54.64368100	34.70468600	36.24779700
H	53.02620700	34.00107800	36.34361700
H	53.83854100	32.57063400	38.29664300
H	55.34110300	33.47876700	38.29326200
H	52.63656200	34.50153200	38.96638300
H	55.97029400	35.53346900	38.45298100
H	56.07764200	36.56258000	39.92558600
H	52.86674100	35.65090400	40.99737300

H	54.17647500	36.84455900	41.17562100
C	47.10494900	38.23002700	31.48523000
C	47.79484100	36.87498100	31.62518500
C	47.70897200	36.22761600	33.01245500
C	46.28561600	36.09938000	33.56978000
C	46.17126000	35.05462200	34.67221600
N	47.12605700	35.27619700	35.80702100
H	46.02781200	38.16189500	31.70487100
H	47.35945300	36.17602100	30.88885400
H	48.85862900	36.97697000	31.35157700
H	48.17105300	35.22622900	32.96069600
H	48.33155500	36.81212900	33.71316500
H	45.92308200	37.07144200	33.93636100
H	45.59969700	35.78413900	32.76524700
H	45.16010900	35.03780100	35.10339200
H	46.39191600	34.04694400	34.28467000
H	46.64858800	35.58158700	36.69172800
H	47.56832600	34.31974300	36.01966300
H	47.86729500	35.94354800	35.58256300
C	49.70007200	40.63309500	33.03394500
C	50.52930000	40.17258500	34.23280000
C	49.69034400	39.29822700	35.15745300
O	51.64104600	39.36883900	33.83609000
H	48.67738800	40.87961600	33.36483700
H	50.88678600	41.04752800	34.80973600
H	52.25155700	39.91054200	33.31945400
H	48.83011200	39.85456500	35.55336600
H	49.30312300	38.42945200	34.60260300
H	50.29792300	38.93976200	36.00162800
P	47.36987900	31.83965500	45.40407700
C	48.43928100	33.74660800	42.93734200
C	48.78064600	34.91410100	42.02364100
C	48.29542700	34.19532200	44.44653800
C	49.56927000	35.99556300	42.74469700
C	48.61688500	36.55497000	43.76422800
C	48.01940500	35.67265100	44.58520100
O	47.25399000	33.46171000	45.06586600
O	46.00235100	31.55626100	46.05416900
O	48.61169400	31.63103700	46.28051000
O	47.47905800	31.10139400	44.02072100
O	47.29383600	33.13513400	42.42233400
O	49.52703900	34.47314600	40.88927300
C	48.15440400	37.97639300	43.60699100
O	47.32567400	38.44648500	44.43366900
O	48.54254500	38.60292900	42.57440200
H	47.20646400	32.28177100	42.91495100
H	51.09792000	33.72868900	41.42932200
H	50.46561800	35.57087300	43.22776700
H	49.91179000	36.76953500	42.05070200
H	47.83435600	35.33949100	41.66995000
H	49.30113800	33.04893700	42.90251200
H	49.25345300	33.97492500	44.95385400
H	47.31766500	35.99140000	45.35324700
P	46.11633300	34.38350500	39.07413800
C	48.86704300	34.04881700	39.68306000
C	48.71576800	32.53926700	39.68411300
C	49.89355600	34.59765600	38.65480400
O	47.65942900	34.76080700	39.51224600
O	45.71448900	35.58174500	38.17166900
O	45.23756900	34.08532000	40.25990700
O	46.18318000	33.05358400	38.18311400
O	50.93834700	33.90607800	38.50632900
O	49.64841400	35.70282100	38.11824700
H	49.66720200	32.10242000	40.00203400
H	47.94188000	32.25769700	40.40778500
H	42.74396100	45.00781600	44.23407000
H	42.06254700	46.07426300	45.48597600
H	43.92308100	40.69452100	34.77863900
H	42.44934900	41.41261600	35.46241900
H	47.19897700	38.60245100	30.45289800
H	50.03108800	41.51384500	32.47407400
H	49.61500100	39.79468600	32.32485200
H	47.52307400	39.00086300	32.14075600
H	40.58205100	24.81738400	39.39184900
H	40.38168400	25.17566700	41.14780500
H	38.50188200	35.38758000	44.47316800
H	38.68297900	35.26009900	42.70721200
H	51.35160900	24.07486600	38.09663400
H	53.04414200	23.52994700	37.90900800
H	50.85419800	25.50839300	46.69251500
H	50.44823800	26.28187500	45.15538500
H	55.91736800	31.41200400	41.79480900
H	55.02461600	31.15426800	43.29148300
H	54.97982600	41.11894200	39.20527000
H	54.25062600	40.17739500	37.88337200
H	58.81858600	39.49867900	43.32694500
H	57.42444400	39.27163600	44.40941200
H	51.73111700	34.63176100	49.79115800

H	50.96550300	33.04694000	49.60774500
H	48.89447300	39.18829000	49.06670600
H	50.59023200	38.63035100	49.01272300
H	46.89390200	29.49646400	49.27840900
H	42.84604600	34.83692200	48.04681600
H	55.20417000	32.00054900	33.70750100
H	53.74424000	32.98304300	33.65502400
H	49.63325900	30.10840300	30.87674400
H	51.31532600	32.96063200	31.71934900
H	51.09313300	30.72883400	31.65500600
H	52.89628200	33.69853200	32.03070700
H	48.73994900	44.25332200	38.59466800
H	47.36081100	43.57844200	39.46732600
H	48.09321100	42.96859000	41.09398500
H	47.66148700	43.48649600	42.74455800
H	41.15736500	34.61070000	35.16518200
H	42.31604800	38.23454600	40.67214900
H	45.68718700	27.81223600	35.73522400
H	45.45700500	28.87028000	37.15369000
H	46.51276000	25.89016700	42.66589100
H	45.59403500	27.23431800	41.96301100
H	46.67291900	27.69776700	40.48966500
H	46.35541500	28.19522300	38.81200200
H	44.63241900	36.85434800	37.36276900
H	46.95591500	32.95257300	37.46619900

TS3(17.6)

C	47.75483400	43.77441800	38.46452800
C	47.91876300	42.50721000	37.61138500
C	48.78201200	41.35652400	38.19168600
C	47.98989600	40.19444500	38.81911000
C	48.70513400	38.83469100	38.77006300
N	47.85482300	37.74284100	39.29526300
H	47.08090300	44.49256500	37.97109600
H	46.92052300	42.10820900	37.35379700
H	48.35900800	42.82007600	36.64871900
H	49.39950800	40.94633100	37.37755100
H	49.51017500	41.73695900	38.92575200
H	47.74457900	40.42498400	39.86839600
H	47.02900700	40.07800400	38.27984300
H	48.97516300	38.58779800	37.73184500
H	49.64963700	38.87033000	39.33411800
H	46.88849100	37.92098300	39.01010800
H	47.85195600	36.26410300	38.95709800
H	47.76839100	37.83107900	40.31196100
C	47.94591200	42.62166500	42.12211000
C	49.21129500	41.89853700	42.58422800
O	49.55625100	40.80740200	41.75275500
H	47.08040700	41.93846700	42.14040200
H	50.05744300	42.60778300	42.57417800
H	49.08801100	41.55748400	43.62698400
H	49.10874800	40.02438200	42.13302700
C	42.49323400	45.08418800	45.30383100
C	43.72561400	44.78709900	46.18759400
C	43.98990800	43.27534100	46.28907800
C	45.27231000	42.90757100	47.04975600
N	45.39110300	41.49231800	47.40466100
C	45.72894800	40.49539900	46.55927800
N	46.00915200	40.73874100	45.28160600
N	45.78273100	39.22677200	46.98468900
H	41.69136800	44.35612200	45.51256900
H	44.61403000	45.30635500	45.78768700
H	43.56700000	45.18777800	47.20381500
H	43.13660800	42.79617900	46.79934300
H	44.01740200	42.83400700	45.27727000
H	46.16811600	43.21107600	46.48051200
H	45.31763800	43.46907300	47.99487700
H	45.14154300	41.22367700	48.34805800
H	45.97436300	41.67075800	44.89694800
H	46.26150900	39.89647400	44.70388100
H	45.72984500	38.95732500	47.95951800
H	46.11315000	38.52842400	46.30048400
C	42.94342300	40.46444000	35.22545900
C	43.23735500	39.66049000	36.49104100
C	44.04526000	38.38711300	36.29356000
O	44.66373700	38.08677500	35.29632100
O	44.02763100	37.62814400	37.39817400
H	42.29118500	39.89898200	34.54210900
H	43.81684900	40.26444900	37.21364100
H	42.31408900	39.38619000	37.02688000
C	41.40500000	35.44494500	35.83059100
C	41.29240800	34.90774200	37.24786400
O	40.21752400	34.79530800	37.81504700
C	40.45987500	36.61765400	35.58451500
H	42.44869800	35.72346300	35.60628300
H	40.74592000	37.48791400	36.19699600
H	40.47350700	36.92423900	34.52800300
H	39.43368500	36.33919100	35.86241400

N	42.46753100	34.52682300	37.84166700
C	42.45106900	34.06998500	39.20836600
C	42.54198500	35.15597100	40.27781500
O	42.33385600	34.87328900	41.45111900
H	43.37120500	34.74051100	37.42265400
H	41.51432300	33.52613900	39.38863700
H	43.29373900	33.39291900	39.39117900
N	42.89724400	36.38635900	39.84219700
C	43.11223000	37.48528900	40.74407800
C	44.43968900	38.22267200	40.55440500
C	44.61952400	39.33251700	41.58070500
O	45.53343100	37.33355000	40.67567200
H	42.98375300	36.55605500	38.84666300
H	43.06974200	37.06997500	41.76131400
H	44.44276600	38.66928700	39.54158600
H	45.27238000	36.43498300	40.38660400
H	43.84471400	40.10709500	41.46820700
H	44.57967400	38.92898300	42.60449300
H	45.60824900	39.79797300	41.45523900
C	38.77050200	34.70820800	43.65759300
C	40.18774500	34.06452400	43.77592000
C	40.32166900	32.93176400	42.75253200
C	41.60449500	32.11880100	42.85485400
N	41.62414200	31.35855300	44.11189700
C	42.70908300	30.97986500	44.80309300
N	43.93054400	31.28613800	44.35040500
N	42.57270800	30.25672500	45.93011800
H	37.99897200	33.91970200	43.64364000
H	40.97707600	34.80731500	43.58804900
H	40.33690800	33.67622300	44.79933100
H	39.45217100	32.24984200	42.83425300
H	40.28757400	33.36252300	41.74187700
H	41.67241200	31.41773700	42.00330600
H	42.46517800	32.79687700	42.80246000
H	40.72203500	31.17483100	44.53655700
H	44.04844200	32.27388800	43.97831100
H	44.75282500	31.08199700	44.94972000
H	41.63472600	30.05151600	46.25495300
H	43.27651400	30.47304700	46.67415100
C	41.02981700	25.29689500	40.27335300
C	41.20561800	26.91152100	40.03475500
C	42.31315800	27.62039600	40.84835300
C	42.75097200	28.93042800	40.16435000
N	43.68244200	29.74317100	40.96056500
C	44.37031800	30.81188900	40.49186600
N	44.43698800	31.06759300	39.18121900
N	45.01388300	31.61613400	41.33869600
H	42.00063600	24.80892300	40.43880600
H	41.43131800	27.05010800	38.96297300
H	40.23300500	27.39814800	40.20775000
H	41.96315400	27.83635400	41.87380000
H	43.19431000	26.96722800	40.95181300
H	43.24379800	28.66721000	39.21382700
H	41.85857400	29.53331500	39.91251600
H	43.60870000	29.68467700	41.97303900
H	43.98508900	30.46616100	38.50746600
H	45.06666000	31.81260900	38.83934700
H	44.85874400	31.51548700	42.33859000
H	45.32665800	32.54890600	41.04782000
C	46.45396200	30.41089600	48.86474800
C	46.56088900	31.70765900	49.66906000
O	46.19210800	31.82710900	50.82339700
C	44.98714700	30.14260900	48.53801400
O	44.45645400	31.16582400	47.70088000
H	46.97887000	30.54165300	47.90864300
H	44.89801400	29.15586000	48.04124600
H	44.38812600	30.11540700	49.46193900
H	45.17124800	31.38211500	47.02743400
N	46.95967400	32.74998700	48.87213300
C	47.03030600	34.14280900	49.26187200
C	45.95740000	35.04035400	48.59623300
O	46.09108900	36.26205300	48.56826600
C	48.41226000	34.71615500	48.94933200
O	48.70845100	34.63075200	47.57025500
H	47.40337300	32.48838100	47.99163500
H	46.84689900	34.18877900	50.34877200
H	48.41894900	35.78198000	49.21129800
H	49.16382500	34.19421500	49.56403500
H	48.77885300	33.69533100	47.29911500
N	44.88004400	34.39462700	48.09552300
C	43.75828400	35.09211000	47.49713400
C	43.55177500	34.73197600	46.02386900
C	44.76258800	35.14855900	45.20953300
C	44.76234300	34.74668400	43.76164400
N	45.23077300	35.63467500	42.89477800
O	44.39013100	33.62440200	43.36760900
H	44.85079900	33.37732300	48.14898900
H	43.94668700	36.16724000	47.61675100

H	43.39377800	33.64462100	45.93425500
H	42.63202700	35.21750200	45.65695600
H	44.93544300	36.23106200	45.29228800
H	45.64284900	34.64532500	45.63438600
H	45.31960000	35.33953100	41.92314200
H	45.53831500	36.55610000	43.18520800
C	51.82895200	33.65803600	49.30006500
C	51.77729500	33.82879900	47.77165000
O	51.51434300	32.63540200	47.04782200
H	52.75533600	33.16599700	49.63399700
H	51.02991700	34.60047900	47.52427000
H	52.75403800	34.20142900	47.41711100
H	50.55932100	32.44414300	47.04806000
C	49.79103400	39.12697900	48.44100500
C	49.48877700	38.34212800	47.14521900
C	50.50967700	38.56679500	46.05199800
C	50.56115700	39.81222700	45.40176600
C	51.45555000	37.60595300	45.67127300
C	51.52683800	40.10454700	44.44926700
C	52.43533600	37.87982300	44.70455900
C	52.49776800	39.14839800	44.09919500
O	53.43027100	39.51899400	43.21010800
H	50.09969100	40.15859900	48.20760200
H	49.39311100	37.26638400	47.36137500
H	48.50129100	38.65641300	46.76794200
H	49.82050800	40.57575600	45.65953700
H	51.43798400	36.62201500	46.14987600
H	51.56659900	41.07530700	43.95525000
H	53.17870000	37.12312300	44.43835600
H	54.07071700	38.80325700	42.93674700
C	54.54491000	40.14800800	38.94602600
C	53.36020700	39.76300500	39.83165200
O	52.21248300	40.55641200	39.54614400
H	55.33930400	39.39317700	39.05338100
H	53.13460200	38.68836000	39.66673800
H	53.63971900	39.85914700	40.89147600
H	51.63652400	40.55464800	40.32736400
C	57.82594900	39.04377800	43.41099000
C	56.84884300	39.56054000	42.32883300
C	56.20691600	38.38315900	41.61582900
O	56.79683600	37.91743300	40.61385100
O	55.13406200	37.89545000	42.10659800
H	57.98348400	37.95733700	43.31940000
H	56.06741500	40.19139100	42.77651600

H	57.38705100	40.15021000	41.57400500
C	54.90504000	31.33989400	42.21885200
C	54.21490700	32.67203300	41.94337900
C	52.73668000	32.69325700	42.21377900
O	52.00623700	31.71669400	42.26323400
O	52.25213000	33.93585300	42.33088600
H	54.34257400	30.50228700	41.78047100
H	54.67738100	33.50973200	42.48665800
H	54.29975400	32.94850400	40.87681000
C	50.15100700	26.19808500	46.21391500
C	50.17506200	27.54336000	46.93938300
O	49.57294500	28.55740700	46.12883400
H	49.14392300	25.75536700	46.19885500
H	49.64073700	27.46199700	47.90215000
H	51.21964200	27.82359900	47.17207700
H	49.30134500	29.31851400	46.67078100
C	45.55198000	26.43195600	42.70694900
C	45.24009100	27.07700400	44.06813600
C	45.21556100	26.04662400	45.19950400
C	46.21208200	28.22224100	44.38092100
H	44.79310900	25.69198400	42.40896700
H	44.22586100	27.51801300	43.99548700
H	46.20902000	25.58136200	45.32193000
H	44.94772800	26.51410800	46.16078300
H	44.49147900	25.23986800	45.00162300
H	47.23492200	27.84976500	44.56020000
H	46.26684400	28.96153300	43.56590600
H	45.92044100	28.77706500	45.28542500
C	46.96524800	28.39866300	39.70156100
C	48.46100200	28.18043600	39.43112600
C	49.27951900	27.97740700	40.71054300
C	49.40749200	29.20747900	41.61464000
C	50.04926700	28.82641500	42.94329400
N	50.25563000	29.97645800	43.84971700
H	46.75904100	29.41579200	40.06885000
H	48.58371800	27.28132100	38.80504800
H	48.88568400	29.01014300	38.84500700
H	48.82542700	27.15496000	41.29288400
H	50.29162600	27.62616500	40.44358200
H	50.01032400	29.98253500	41.10970700
H	48.42169300	29.65699200	41.81590300
H	49.40306400	28.11933100	43.48134900
H	51.01831700	28.32622300	42.78017700
H	51.00276400	30.59959300	43.51863200

H	49.32101600	30.56780000	43.96032900
H	50.43806800	29.61743400	44.80220100
C	45.80608800	28.83341900	36.11304000
C	47.32257400	29.07086500	36.15164500
C	47.81080300	30.37757300	36.75998700
C	47.69103700	31.62717200	35.89888200
O	47.42551800	31.59596800	34.70607300
O	47.92850400	32.73851700	36.56540900
H	45.29515300	29.53651100	35.43765300
H	47.79136300	28.24463800	36.70801100
H	47.72289600	28.99113900	35.13139300
H	47.32454600	30.58025600	37.72941100
H	48.88792400	30.29500300	36.97766600
H	48.81653600	32.52597000	38.91426200
C	52.34820500	24.35403100	37.72083700
C	52.87186500	25.63804800	38.39003100
C	52.07882500	26.84099900	37.84150400
C	52.45498400	28.21336700	38.39037500
N	51.78163700	29.26780700	37.63873400
C	51.77064900	30.55121000	37.99503700
N	52.16668000	30.94511400	39.23412200
N	51.38794400	31.49332700	37.13941100
H	52.27746300	24.48399300	36.62950900
H	52.77651400	25.57161500	39.48785400
H	53.94468400	25.77186000	38.16982800
H	52.20325900	26.86897200	36.74527800
H	50.99962700	26.68067700	38.01075800
H	52.17337600	28.27045400	39.45756700
H	53.55127800	28.35593400	38.34371100
H	51.41856100	29.10205900	36.66753900
H	52.22285600	30.28096400	39.99412300
H	51.95074300	31.91049200	39.47835200
H	51.18751600	31.32512500	36.12095900
H	51.39017200	32.44750000	37.49786600
C	50.01095600	30.57607000	31.79195200
C	49.70584200	29.71703300	33.01761700
C	50.41611800	30.10974400	34.31013400
O	50.73567700	31.33625700	34.49287100
O	50.61833700	29.21308700	35.15914300
H	49.55265000	31.57299500	31.87981100
H	48.62816200	29.77432300	33.24879500
H	49.93697800	28.65693500	32.83607600
C	51.92989200	33.85217200	31.53932800
C	51.22435500	35.08963400	32.07096800

C	51.00042000	35.04922500	33.54334800
C	51.12660700	36.05558800	34.47074100
N	50.53182300	33.93589200	34.23052200
C	50.36448300	34.25703900	35.51384400
N	50.71186200	35.53550000	35.68116800
H	52.10438100	33.93572700	30.45655600
H	51.79062900	36.00605000	31.84616500
H	50.24979800	35.20278000	31.56557100
H	50.47469200	32.91136300	33.96498400
H	51.45674700	37.08466500	34.33691900
H	49.94091600	33.59462900	36.26455200
H	50.72411400	35.94570200	36.63657300
C	54.69423200	32.85224800	34.18404200
C	54.56562100	32.58897300	35.74411700
C	54.27791400	33.81369000	36.60427300
C	54.63461000	33.50179300	38.06174400
N	54.04870100	34.38407300	39.04398600
C	54.63365100	35.43683100	39.65958700
N	55.86053500	35.83930400	39.35279600
N	53.95080900	36.04808400	40.62874100
H	55.31684400	33.74205700	33.99669400
H	53.79506700	31.82186600	35.92133300
H	55.52429300	32.14832500	36.07040900
H	54.85228100	34.68882900	36.25410900
H	53.21412000	34.09683200	36.54713600
H	54.28275700	32.48161800	38.29354500
H	55.72770900	33.47606700	38.19698800
H	53.02907300	34.31624500	39.09443500
H	56.28311200	35.51352300	38.49454800
H	56.28408500	36.68165700	39.87877700
H	53.13411600	35.58010600	41.00210400
H	54.40942000	36.81258100	41.20991200
C	47.10492700	38.22938400	31.48633700
C	47.87758800	36.91782600	31.56213000
C	47.72720400	36.17682600	32.89104300
C	46.28342100	35.78772300	33.24041200
C	46.23112200	34.63811700	34.23480400
N	46.97160100	34.95477500	35.49196200
H	46.04547700	38.09962200	31.75556900
H	47.54450500	36.24655600	30.75043700
H	48.94815300	37.11167500	31.37752800
H	48.34469200	35.26327700	32.85908700
H	48.15051300	36.81104500	33.69246800
H	45.72744600	36.65104900	33.63814000

H	45.75767300	35.45670100	32.32916300
H	45.19880700	34.39714700	34.52647300
H	46.68265600	33.72001300	33.83015200
H	46.33677400	35.30630500	36.25608600
H	47.43017100	34.05972300	35.87920400
H	47.71165700	35.64306800	35.33660900
C	49.69927000	40.63387900	33.03499800
C	50.62435300	40.10426600	34.13084400
C	49.84940400	39.24987100	35.12408200
O	51.65220200	39.26312900	33.60006000
H	48.72907500	40.92247600	33.47196700
H	51.08189300	40.94818000	34.68228800
H	52.23499000	39.79581700	33.04365600
H	49.05583900	39.83626600	35.60481200
H	49.37985400	38.39883900	34.60532100
H	50.51734900	38.86692100	35.90954200
P	47.61599300	31.93425300	45.44152400
C	48.56601800	33.99956900	42.95424200
C	48.65139100	35.23983800	42.06726900
C	48.38820000	34.37685600	44.49449600
C	49.21391300	36.46622900	42.74773900
C	48.27224500	36.78599100	43.86550500
C	47.93160700	35.79688200	44.70219400
O	47.40829500	33.53791200	45.07267100
O	46.20718700	31.47960000	45.84429300
O	48.67283200	31.85996300	46.56090100
O	48.07671000	31.27304100	44.09808000
O	47.53410200	33.19819200	42.48124300
O	49.53302200	35.07551100	40.88876400
C	47.58033600	38.12346700	43.83924400
O	46.54460500	38.24568500	44.57730900
O	47.97430300	38.99475600	43.04076200
H	47.59613500	32.34323900	42.99197300
H	51.29373000	33.88671700	42.50511400
H	50.23517500	36.27615800	43.12343500
H	49.27930800	37.30114300	42.04172200
H	47.65832500	35.43006800	41.64025400
H	49.53772900	33.45526800	42.88819600
H	49.36380800	34.21680000	44.99313600
H	47.27509400	35.97248800	45.55020900
P	46.14800800	34.66326800	38.79550300
C	49.48350000	34.25504600	39.92507800
C	48.90585000	32.90046900	39.93953700
C	50.51801700	34.63225800	38.87355200
O	47.67877700	35.19394000	38.79027400
O	45.40332800	35.40588700	37.65457200
O	45.50699300	34.68689300	40.16941500
O	46.28495000	33.10305400	38.40080200
O	51.40711100	33.73176500	38.79534200
O	50.43887000	35.71487200	38.30063700
H	49.63243000	32.27206500	40.48538400
H	47.96900600	32.86116500	40.50291300
H	42.73826500	45.00208400	44.23322700
H	42.06219200	46.07404100	45.48634700
H	43.87405200	40.68505600	34.68142500
H	42.44992000	41.41298000	35.46215600
H	47.13153200	38.63689500	30.46301700
H	50.03162800	41.51329800	32.47353500
H	49.50462300	39.82359100	32.31582600
H	47.52324600	39.00139300	32.14006700
H	40.53375100	24.85175500	39.40078600
H	40.38179100	25.17574000	41.14789400
H	38.50212800	35.38786500	44.47301100
H	38.68253000	35.26257700	42.70970100
H	51.35134400	24.07569200	38.09595200
H	53.04414200	23.52993900	37.90898200
H	50.85413500	25.50840000	46.69261600
H	50.48549600	26.32825600	45.17136300
H	55.91046300	31.34268200	41.77388100
H	55.02464600	31.15439300	43.29150000
H	54.97983600	41.11893700	39.20527500
H	54.23605000	40.18189600	37.88762300
H	58.81854800	39.49874700	43.32685900
H	57.44762800	39.23186900	44.42659700
H	51.73140600	34.63180600	49.79112700
H	50.98296800	33.03554900	49.63551500
H	48.89446100	39.18830700	49.06668700
H	50.60409900	38.66233400	49.01968200
H	46.89340300	29.49640400	49.27880800
H	42.84629500	34.83599900	48.04680200
H	55.19448300	31.99342300	33.71038700
H	53.74421500	32.98298000	33.65505900
H	49.63338100	30.10827000	30.87676100
H	51.33046200	32.94732500	31.70563600
H	51.09619700	30.71074000	31.66066100
H	52.89631200	33.69828800	32.03057300
H	48.72774500	44.27476300	38.60259800
H	47.36090400	43.57973300	39.46762200

H	48.09360700	42.96788400	41.09383400
H	47.72787400	43.49561500	42.75937800
H	41.15752200	34.60997100	35.16603700
H	42.31616500	38.23461000	40.67152300
H	45.68756200	27.81252900	35.73430900
H	45.34546500	28.89797000	37.11294800
H	46.53491600	25.93074300	42.71938400
H	45.59413300	27.23437900	41.96310100
H	46.67265100	27.69766400	40.48947400
H	46.35192000	28.22275500	38.80995600
H	44.64135500	36.83356800	37.36816700
H	46.99825800	32.91005700	37.66815700

Int3(10.4)

C	47.75559500	43.77622400	38.46526200
C	47.79257300	42.52202500	37.58637400
C	48.63417700	41.34670800	38.11555700
C	47.92417000	40.39813900	39.09177700
C	48.74440600	39.12888500	39.29092900
N	48.15291900	38.21864200	40.31548100
H	47.13110600	44.56081100	38.01016500
H	46.76283000	42.16821200	37.39394100
H	48.19227300	42.81246400	36.59948900
H	48.96136800	40.74715700	37.25296700
H	49.56566700	41.71431700	38.57981800
H	47.77514900	40.88376600	40.06600700
H	46.93177500	40.12009300	38.69347400
H	48.79985900	38.55303800	38.35597900
H	49.77199200	39.37349600	39.59698500
H	47.09862000	38.15612600	40.31332700
H	48.45297900	37.24975000	40.15170800
H	48.37086600	38.48696200	41.30521500
C	47.94696600	42.62103400	42.12202200
C	49.25696300	42.04785900	42.66192700
O	49.73185900	40.94229400	41.91185700
H	47.15311200	41.85604300	42.12921100
H	50.03280200	42.83310100	42.63901000
H	49.13056600	41.75678500	43.71962400
H	49.44734000	40.14294900	42.39574600
C	42.49300100	45.08398500	45.30399000
C	43.72501100	44.78702500	46.18801000
C	43.99017500	43.27385000	46.28334800
C	45.19328200	42.89111400	47.15761400
N	45.28393400	41.46564000	47.48358100
C	45.82971800	40.51038200	46.70354200
N	46.32030300	40.80290200	45.49966000
N	45.89167200	39.24004000	47.12287500
H	41.69096600	44.35663400	45.51336700
H	44.61440600	45.31161700	45.79796900
H	43.55779400	45.17928100	47.20630100
H	43.09480600	42.78377300	46.70325700
H	44.11291200	42.85011900	45.27025600
H	46.14223300	43.20729400	46.69269500
H	45.13952500	43.42796200	48.11685800
H	44.86270100	41.15812400	48.35160600
H	46.18695500	41.71407200	45.08661600
H	46.71855400	39.99695600	44.95803300
H	45.70927800	38.95220900	48.07733600
H	46.36824600	38.56641600	46.51139000
C	42.94450600	40.46519800	35.22513200
C	43.72094900	39.93613100	36.43257800
C	44.21558100	38.50038900	36.31666600
O	43.88008100	37.73230000	35.43353000
O	45.02631000	38.16232600	37.31291800
H	42.17393500	39.74129700	34.92090100
H	44.58828800	40.57169000	36.67954800
H	43.08839100	39.95417100	37.33882300
C	41.40497100	35.44397400	35.83106900
C	41.32863500	34.89119900	37.24656900
O	40.26781400	34.77415600	37.84182400
C	40.43621300	36.60275100	35.61578000
H	42.43367600	35.76038700	35.59736700
H	40.72814500	37.47104100	36.22754500
H	40.42823400	36.92061000	34.56241400
H	39.41896800	36.30947000	35.91166500
N	42.51352700	34.49269400	37.80372800
C	42.51732300	34.08906500	39.19081400
C	42.34684600	35.24769000	40.17273700
O	41.72079000	35.13419300	41.21727200
H	43.40985200	34.79566900	37.41870100
H	41.70176900	33.38073000	39.38239600
H	43.48445000	33.61783700	39.40962200
N	42.99745200	36.38687500	39.82328800
C	43.11198400	37.48602100	40.74449800
C	44.38861600	38.30665400	40.52513800
C	44.39382000	39.56549100	41.38554500
O	45.57639600	37.59362300	40.84386900
H	43.55733000	36.38240600	38.97355200

H	43.07256700	37.08768200	41.76945000
H	44.42531400	38.59796400	39.46104000
H	45.76521000	36.73215300	40.32483600
H	43.53250400	40.21102800	41.15694700
H	44.36253100	39.30173400	42.45503800
H	45.31425200	40.14153800	41.20489300
C	38.77099200	34.70900000	43.65697200
C	40.18799700	34.06400100	43.77703400
C	40.41020200	32.91857000	42.78739100
C	41.86262200	32.46591600	42.66708500
N	42.35034000	31.78930400	43.88273600
C	43.62184900	31.44470500	44.10522500
N	44.59485000	31.89557100	43.27950700
N	43.96250700	30.60438500	45.09068800
H	37.99419000	33.92483100	43.63414800
H	40.95160600	34.83285400	43.58418800
H	40.34571400	33.70726300	44.81200800
H	39.75671600	32.05789500	43.03054800
H	40.12371800	33.26525800	41.78426300
H	41.96906400	31.76990700	41.81488100
H	42.48439300	33.34531700	42.46452500
H	41.65423900	31.43688200	44.53071700
H	44.51164700	32.92722000	43.04948200
H	45.53417100	31.66155300	43.61008400
H	43.29693200	30.41318100	45.83258500
H	44.94328800	30.72329400	45.41619100
C	41.02907000	25.29701500	40.27307600
C	41.20597000	26.91098700	40.03497300
C	42.39674000	27.26718600	39.12488600
C	42.59463400	28.75336200	38.73800700
N	43.41646500	29.53458700	39.67487100
C	44.09147400	30.67324900	39.37370300
N	44.16085500	31.14615700	38.12945000
N	44.72917200	31.36874100	40.31950400
H	42.00471700	24.83443600	40.48564100
H	40.27059200	27.30504000	39.60561000
H	41.32569500	27.38451500	41.02517700
H	43.33787400	26.88918800	39.56238200
H	42.27366200	26.71857500	38.17630200
H	43.10467500	28.77343400	37.76389200
H	41.61380000	29.24218700	38.59614700
H	43.41843000	29.25488300	40.64882200
H	43.91752600	30.59600800	37.31900000
H	44.74305900	31.98799800	37.98005700
H	44.56034600	31.25409800	41.32000100
H	45.21442300	32.23095600	39.99102800
C	46.45306400	30.41005800	48.86596800
C	46.77921000	31.68149200	49.64612500
O	46.82472600	31.75073400	50.86076000
C	44.94088600	30.27808000	48.66397000
O	44.42328900	31.35091300	47.88444900
H	46.90804700	30.49686900	47.86997600
H	44.72905100	29.30561200	48.17542500
H	44.41347400	30.28369400	49.63104300
H	45.09590000	31.53409600	47.18971700
N	46.91017500	32.78003500	48.82485900
C	46.99030700	34.14656100	49.30261200
C	45.91934700	35.05653500	48.66328300
O	46.05077900	36.27923500	48.63777100
C	48.36774100	34.76314400	49.07443700
O	48.66021500	34.90526500	47.69997600
H	46.83805800	32.61876800	47.82284200
H	46.80298600	34.10469600	50.38929200
H	48.38484000	35.77418000	49.50273700
H	49.11976800	34.14440500	49.59158000
H	48.50795300	34.06859300	47.23213600
N	44.85604300	34.40429500	48.14356000
C	43.75795600	35.09204900	47.49700300
C	43.63094200	34.75075600	46.00722000
C	44.96181400	35.01388300	45.28541400
C	44.83795000	35.33049500	43.80977600
N	45.27769600	36.53982500	43.44488500
O	44.34882300	34.53515400	42.98943800
H	44.78527700	33.39170500	48.25572300
H	43.93812100	36.16857400	47.62327100
H	43.32222800	33.70075200	45.88029200
H	42.82131500	35.36318300	45.57663700
H	45.46722500	35.85186000	45.78497400
H	45.63043600	34.14676600	45.38877300
H	45.32914700	36.80841800	42.45450000
H	45.73401900	37.15777800	44.11366800
C	51.82904200	33.65801000	49.29998600
C	51.70477200	33.84454100	47.77796600
O	51.53184200	32.65147000	47.02873800
H	52.78403200	33.19143400	49.58654200
H	50.88471800	34.55265000	47.57506500
H	52.63167800	34.31209800	47.40109600
H	50.60996500	32.34112900	47.07557400

C	49.79102800	39.12700200	48.44102300
C	49.52978800	38.36534000	47.13401500
C	50.56248600	38.64718100	46.06614900
C	50.63244100	39.92873000	45.49179300
C	51.49024700	37.69542600	45.62398600
C	51.58923600	40.25804900	44.54098300
C	52.45814600	38.00560200	44.65854600
C	52.52642400	39.30053100	44.11451900
O	53.44374400	39.68681700	43.21091700
H	50.10126100	40.16404700	48.23244600
H	49.45530900	37.28380900	47.32262500
H	48.54896900	38.66751400	46.74196000
H	49.91189500	40.69005900	45.80713000
H	51.46004100	36.68715600	46.04770000
H	51.64370000	41.25633200	44.10595400
H	53.18571700	37.25407300	44.34014200
H	54.03627700	38.94529900	42.90669200
C	54.54499500	40.14801600	38.94596200
C	53.35284600	39.77673000	39.82885300
O	52.23156200	40.62205500	39.60272900
H	55.33277400	39.38452900	39.05732100
H	53.07730800	38.72206100	39.60743400
H	53.64361100	39.80317700	40.88770300
H	51.75302400	40.73611300	40.43927100
C	57.82597500	39.04397900	43.41107900
C	56.85420400	39.39498000	42.26099400
C	56.15510800	38.14734000	41.73589000
O	56.82443300	37.40552000	40.96998700
O	54.96218400	37.88613200	42.09063300
H	58.01284500	37.95816600	43.43602000
H	56.10563000	40.12993900	42.58892600
H	57.40810200	39.81963800	41.41090200
C	54.90496900	31.34006700	42.21899900
C	54.23428300	32.67173300	41.89412900
C	52.72640100	32.70739200	42.04703800
O	52.03959200	31.71906900	42.24175100
O	52.13688700	33.88943900	41.88376400
H	54.33523300	30.50353900	41.78873500
H	54.66910400	33.50786200	42.46587300
H	54.41337900	32.92383800	40.83658300
C	50.15102500	26.19806200	46.21392600
C	50.26059600	27.61153300	46.79405700
O	49.62193900	28.54781500	45.92895700
H	49.12837000	25.80129000	46.30533100
H	49.80189900	27.64420300	47.79822000
H	51.32704200	27.88016700	46.92153900
H	49.33670700	29.37194100	46.38637100
C	45.55198000	26.43195600	42.70694900
C	45.41599100	27.03810500	44.11211700
C	45.40314500	25.95229300	45.19155600
C	46.51102900	28.07452700	44.38412100
H	44.71169400	25.76261000	42.46184000
H	44.44489100	27.56809500	44.15359800
H	46.36113900	25.40414800	45.20324700
H	45.25828000	26.38589700	46.19418300
H	44.60010000	25.21699100	45.02158500
H	47.51225600	27.61374000	44.38855200
H	46.51539100	28.88615600	43.64013000
H	46.38750000	28.54817000	45.37038100
C	46.96509200	28.39857700	39.70156000
C	48.44173300	28.17748500	39.35606800
C	49.32491000	28.00867700	40.59506200
C	49.40250800	29.24245500	41.49547000
C	50.14278500	28.93333400	42.78868300
N	50.17431500	30.08396000	43.71620200
H	46.76830100	29.41577300	40.07497500
H	48.53752500	27.26923100	38.73824600
H	48.82570100	29.00236800	38.73385300
H	48.94155500	27.16077500	41.19129100
H	50.34400300	27.71864100	40.28671900
H	49.89868900	30.07088500	40.95974000
H	48.39167700	29.59691700	41.75280300
H	49.63932100	28.11803600	43.32584700
H	51.17810400	28.61076600	42.59319700
H	50.87850800	30.78118100	43.43743200
H	49.20755800	30.56787800	43.76291000
H	50.33162500	29.73240400	44.67995800
C	45.80675800	28.83400700	36.11091600
C	47.27263100	29.25604100	36.03992700
C	47.61750100	30.56540800	36.75438700
C	47.06292500	31.84059500	36.13784700
O	45.84705200	31.89693200	35.81719500
O	47.87084300	32.81724400	36.02279800
H	45.17059100	29.51191900	35.52503000
H	47.90736900	28.46532400	36.47032900
H	47.58388900	29.32923600	34.98514000
H	47.22740500	30.54179700	37.78800700
H	48.70819700	30.67375800	36.81880600

H	48.62066500	32.60456500	39.02066600
C	52.34794600	24.35396600	37.72109000
C	52.87198700	25.63799600	38.38999300
C	52.08002800	26.84850400	37.85926900
C	52.50787600	28.22289100	38.37077800
N	51.82202400	29.27277900	37.62596400
C	51.71620900	30.54643800	37.99427300
N	52.04157200	30.96186500	39.25164700
N	51.31707700	31.46981300	37.12779500
H	52.27619800	24.48430200	36.62977800
H	52.78882400	25.56569800	39.48842700
H	53.94329600	25.77146900	38.16069300
H	52.15911800	26.86217800	36.75851600
H	51.00561100	26.71355500	38.07370100
H	52.27822100	28.30726000	39.44775700
H	53.60382200	28.34019900	38.27193900
H	51.49069800	29.10242700	36.64100600
H	52.08401600	30.30081300	40.01610900
H	51.73643800	31.90233300	39.49320800
H	51.14345100	31.29160300	36.10689000
H	51.17472300	32.40947800	37.49641500
C	50.01101800	30.57589000	31.79200000
C	49.93947300	29.58401500	32.94838400
C	50.54636600	30.03403500	34.27688000
O	50.76494300	31.27757700	34.46549900
O	50.77438700	29.14518800	35.12696700
H	49.41858400	31.48180200	31.99283000
H	48.88942600	29.31747600	33.16351600
H	50.42797200	28.63238500	32.68686600
C	51.92998800	33.85204100	31.53906900
C	51.21170600	35.07848500	32.09582700
C	50.96554400	35.03586100	33.56919700
C	51.04092200	36.04518400	34.50034500
N	50.49889000	33.91634800	34.24780000
C	50.28173400	34.23366500	35.52393200
N	50.60098500	35.51635200	35.70229900
H	52.10428700	33.96195500	30.45891400
H	51.77449000	35.99982600	31.88144800
H	50.23872400	35.19351300	31.58576000
H	50.46313600	32.90211300	33.97011200
H	51.35643200	37.08038000	34.38177100
H	49.83298000	33.55837400	36.24423700
H	50.54778700	35.96164100	36.63458400
C	54.69387500	32.85178400	34.18400600

C	54.56613400	32.58919300	35.74404000
C	54.11645100	33.79371900	36.55962500
C	54.39377800	33.54258100	38.03938400
N	53.77479000	34.51459200	38.92001700
C	54.37972500	35.31159400	39.80706900
N	55.68289200	35.38447900	39.96829000
N	53.56631400	36.01767300	40.67118500
H	55.31131000	33.74615100	34.00082600
H	53.87693100	31.74663800	35.91647300
H	55.56008600	32.26315700	36.09804200
H	54.64239200	34.70937600	36.23993400
H	53.03976800	33.98081700	36.41919500
H	54.01189100	32.54458800	38.31592100
H	55.47941000	33.50919100	38.21345400
H	52.74942500	34.46080600	38.90651800
H	56.26382900	34.82929600	39.35170700
H	56.17314500	36.26412000	40.48414700
H	52.71990400	36.38297900	40.23234000
H	54.07835100	36.74724600	41.24510000
C	47.10493600	38.23018100	31.48473400
C	47.64820900	36.83313200	31.79637200
C	47.15814200	36.18929400	33.10332600
C	45.62389100	36.06117800	33.19661300
C	45.12769200	34.76711200	33.83482600
N	45.70402600	34.48328200	35.16802200
H	46.00763500	38.27270900	31.56612700
H	47.38016900	36.15480100	30.96622100
H	48.75103900	36.87084600	31.81071100
H	47.62732200	35.18928300	33.17316900
H	47.53960900	36.75831500	33.96991500
H	45.18709200	36.91466600	33.73449800
H	45.18957100	36.08155400	32.18269300
H	44.03443500	34.78835700	33.94740400
H	45.38193700	33.90325900	33.19963200
H	45.56209800	33.45500300	35.44570300
H	46.73118800	34.52494900	35.16188600
H	45.18580400	37.14661600	37.36012800
C	49.69965200	40.63345600	33.03440700
C	50.51555600	40.14850800	34.23102600
C	49.66906200	39.22772100	35.10290500
O	51.65060200	39.37834900	33.83188900
H	48.67899700	40.88625100	33.36708800
H	50.84467400	41.01027000	34.84427100
H	52.24081400	39.93934800	33.31227500

H	48.78537500	39.75493300	35.48631100
H	49.31764400	38.36513800	34.51538800
H	50.25228500	38.85964600	35.96045300
P	47.76040200	31.77338100	45.42745700
C	48.61363800	33.98516500	43.03828900
C	48.78920100	35.27013700	42.23215300
C	48.68702800	34.31463400	44.59653700
C	49.58438700	36.32550700	42.96852600
C	48.67025400	36.72693500	44.08240300
C	48.23813000	35.73237600	44.87449400
O	47.86499800	33.44788300	45.35860000
O	46.33197700	31.61965800	45.99189300
O	48.90975300	31.25927100	46.29050700
O	47.82765400	31.25539300	43.94664400
O	47.39950300	33.41241700	42.67131000
O	49.48224100	35.14451000	40.93440000
C	48.04891800	38.08838800	44.00290100
O	47.13184300	38.37848100	44.83025000
O	48.38311700	38.84054100	43.04794600
H	47.43953000	32.48677100	43.04098600
H	52.75705900	34.63302100	41.64526300
H	50.53369400	35.90548900	43.33523800
H	49.82323000	37.17716200	42.32642200
H	47.79070700	35.62301400	41.93469300
H	49.46707600	33.31267800	42.82701200
H	49.74392700	34.18726700	44.90513500
H	47.55220000	35.89139400	45.70216600
P	46.24726900	34.82883800	38.53475000
C	49.38604300	34.32259500	39.98791000
C	48.69334100	33.02999000	40.02501500
C	50.34038900	34.73777300	38.87661100
O	46.64100000	35.62660000	39.79200700
O	45.23694100	35.62069800	37.61170100
O	45.75967000	33.38332200	38.77095000
O	47.66649800	34.74730500	37.70804300
O	51.13856900	33.79217200	38.60498700
O	50.35084900	35.90143600	38.47303100
H	47.70896200	33.14641800	40.49950200
H	49.28715400	32.36436400	40.67603100
H	42.73668800	45.00192600	44.23304300
H	42.06261600	46.07430200	45.48592800
H	43.60622200	40.62248700	34.35954100
H	42.44870400	41.41235100	35.46213400
H	47.36530100	38.52054600	30.45442900
H	50.03140800	41.51352800	32.47376700
H	49.60320000	39.80143600	32.31888600
H	47.52292100	39.00062100	32.14108200
H	40.58026100	24.81630900	39.39286100
H	40.38209500	25.17566000	41.14810900
H	38.50189900	35.38756300	44.47318600
H	38.69876900	35.26571200	42.70934000
H	51.35142800	24.07484100	38.09667300
H	53.04428000	23.53001700	37.90879900
H	50.85412700	25.50835900	46.69257400
H	50.40751100	26.22200500	45.14130000
H	55.91372500	31.32078400	41.78035800
H	55.02474400	31.15418400	43.29145600
H	54.97980000	41.11894900	39.20528800
H	54.24553000	40.18034300	37.88553300
H	58.81860000	39.49863500	43.32688500
H	57.40591600	39.32238400	44.38896500
H	51.73131600	34.63181400	49.79109000
H	51.01530800	33.01483100	49.67427700
H	48.89446500	39.18829200	49.06669300
H	50.59824700	38.66342000	49.02966800
H	46.89411600	29.49650100	49.27826600
H	42.84610000	34.83673800	48.04681800
H	55.20089500	31.99692900	33.71062600
H	53.74427200	32.98315700	33.65500200
H	49.63333400	30.10844000	30.87669700
H	51.33614900	32.94095700	31.68800700
H	51.05060200	30.88095000	31.60102300
H	52.89625900	33.69840200	32.03070300
H	48.76954300	44.18906900	38.59571300
H	47.36019600	43.57728900	39.46682000
H	48.09328700	42.96949100	41.09426700
H	47.61616300	43.48069400	42.72938100
H	41.15738000	34.61073400	35.16513400
H	42.31607100	38.23453000	40.67172100
H	45.68709400	27.81200300	35.73588600
H	45.44706700	28.84518400	37.15099700
H	46.48536800	25.84984300	42.61635500
H	45.59405900	27.23433400	41.96303100
H	46.67293400	27.69772200	40.48963200
H	46.31527300	28.23492700	38.83309500
H	45.38788800	35.09061400	35.97193200
H	47.70006900	33.97245800	37.07815600

TS4(13.6)

C	47.75560000	43.77622200	38.46526200
C	47.79826100	42.52917300	37.57737300
C	48.65713400	41.36012100	38.08912100
C	47.97727000	40.42578700	39.09768000
C	48.79963200	39.15920300	39.30070800
N	48.21960700	38.27269700	40.35034300
H	47.12979400	44.56150100	38.01281700
H	46.77061100	42.16521900	37.39152500
H	48.18578900	42.83180000	36.58924700
H	48.95553500	40.75127700	37.22248400
H	49.60294000	41.73227500	38.51973400
H	47.85213300	40.92671400	40.06706000
H	46.97402000	40.14196900	38.73006700
H	48.83285300	38.56400800	38.37692700
H	49.83314500	39.40567300	39.58395000
H	47.17418000	38.24688200	40.35846100
H	48.46651800	37.29010200	40.16439100
H	48.46642700	38.53583100	41.33401000
C	47.94695900	42.62105100	42.12202900
C	49.26570300	42.07821000	42.67280400
O	49.76853700	40.98063600	41.92930400
H	47.17062100	41.83834900	42.12657700
H	50.02430300	42.88031700	42.65278300
H	49.13839000	41.78691100	43.73012700
H	49.50847300	40.17855300	42.42199400
C	42.49301400	45.08399900	45.30397900
C	43.72501700	44.78701400	46.18800000
C	43.99146500	43.27319000	46.27691100
C	45.19695100	42.88439200	47.14552500
N	45.28508400	41.45742400	47.46589400
C	45.82741800	40.50289100	46.68198200
N	46.31675600	40.79643000	45.47816600
N	45.88579000	39.23069400	47.09682300
H	41.69111200	44.35643100	45.51311300
H	44.61372500	45.31420400	45.79994800
H	43.55664800	45.17527800	47.20759200
H	43.09711700	42.78149000	46.69713500
H	44.11122600	42.85379600	45.26167300
H	46.14492500	43.20006200	46.67825400
H	45.14814900	43.41783400	48.10696200
H	44.86622800	41.14801000	48.33439800
H	46.19393400	41.71138700	45.07038700
H	46.71703600	39.99019100	44.93639700
H	45.70890200	38.94009700	48.05170500
H	46.36431500	38.56084400	46.48218700
C	42.94451100	40.46520400	35.22505600
C	43.64323600	39.89471200	36.45461700
C	44.16071800	38.47417600	36.30651100
O	43.91915300	37.74279000	35.36337500
O	44.88556900	38.09968600	37.35360500
H	42.18272500	39.76246300	34.85579700
H	44.48774100	40.52448900	36.78250700
H	42.95534800	39.86817400	37.31914300
C	41.40497700	35.44399400	35.83106300
C	41.33113100	34.88843700	37.24566000
O	40.27404700	34.78042800	37.84844800
C	40.43650200	36.60320500	35.61793000
H	42.43400700	35.75861200	35.59439400
H	40.72805400	37.47074000	36.23107100
H	40.42765500	36.92235300	34.56506900
H	39.41955800	36.30988500	35.91451400
N	42.51652900	34.47772100	37.79228800
C	42.52944800	34.09042700	39.18294800
C	42.34374900	35.25791300	40.15518800
O	41.69492000	35.15148900	41.18542900
H	43.41376400	34.77328600	37.40382600
H	41.72608500	33.37146500	39.38745700
H	43.50013900	33.62992400	39.41063000
N	43.00750200	36.39207800	39.81522400
C	43.11198200	37.48600100	40.74437000
C	44.39264900	38.31323200	40.55961500
C	44.38870800	39.53917300	41.46626200
O	45.57918100	37.58913600	40.86388000
H	43.57012600	36.39094400	38.96848700
H	43.05976300	37.07729800	41.76468600
H	44.43850000	38.63994600	39.50717700
H	45.83812000	36.87005400	40.20736900
H	43.51761800	40.18172300	41.26865200
H	44.36826700	39.23454500	42.52489100
H	45.30104200	40.13266100	41.30208500
C	38.77099000	34.70899600	43.65697000
C	40.18800300	34.06400600	43.77703400
C	40.41726200	32.92655000	42.77557200
C	41.87564200	32.49805800	42.61614700
N	42.40821600	31.82112400	43.81069000
C	43.68114000	31.45959000	43.99080900
N	44.62697600	31.84347800	43.09130700

N	44.04819500	30.67673800	45.01058300
H	37.99405000	33.92506400	43.63285100
H	40.95105600	34.83636500	43.59519400
H	40.34185500	33.69975800	44.81007000
H	39.78345400	32.05321100	43.02421200
H	40.10510200	33.27542100	41.78081100
H	41.97039000	31.80934300	41.75631500
H	42.47865500	33.38806500	42.40495900
H	41.74678600	31.55822300	44.53314200
H	44.56804600	32.87772500	42.88948800
H	45.57235600	31.58065000	43.38717800
H	43.39469600	30.51366500	45.77129200
H	45.02981300	30.82750800	45.33566200
C	41.02907400	25.29701400	40.27307300
C	41.20596300	26.91098600	40.03497700
C	42.41046900	27.26561300	39.15377100
C	42.52525800	28.72952400	38.67666400
N	43.28194800	29.63492000	39.55411500
C	44.00007400	30.69758000	39.11107600
N	43.95978100	31.03272400	37.82169700
N	44.75884100	31.41549900	39.94424100
H	42.00430300	24.83421400	40.48703800
H	40.27871100	27.29507600	39.57927100
H	41.29674400	27.39162500	41.02446100
H	43.35410700	26.97216100	39.64654100
H	42.35368000	26.65338400	38.23821800
H	43.05609200	28.70854800	37.71460500
H	41.51764200	29.14010900	38.48326400
H	43.25617500	29.47421100	40.55452500
H	43.39955100	30.48239200	37.18782400
H	44.70322200	31.58774300	37.35056000
H	44.70452000	31.29783000	40.95770400
H	45.24730300	32.25805800	39.58199800
C	46.45305100	30.41007000	48.86599400
C	46.76920400	31.68154700	49.65060500
O	46.80750900	31.74768000	50.86582000
C	44.94226300	30.26422400	48.65535400
O	44.41679900	31.32679800	47.86611700
H	46.91291900	30.50028200	47.87273300
H	44.74301800	29.28725200	48.17100000
H	44.41020900	30.26926900	49.62001300
H	45.10181600	31.52626400	47.18835500
N	46.90271500	32.78115200	48.83154900
C	46.98482400	34.14667200	49.31157900

C	45.90811900	35.05803600	48.68422500
O	46.02834700	36.28184500	48.67985900
C	48.35802500	34.76958700	49.07058300
O	48.62649500	34.94494100	47.69519000
H	46.83293600	32.61967500	47.82847000
H	46.80846500	34.10212000	50.39989100
H	48.38201100	35.77060500	49.52164000
H	49.11980000	34.14045700	49.56037400
H	48.46922500	34.12098400	47.20592000
N	44.85488900	34.40490600	48.14502600
C	43.75796400	35.09204300	47.49700600
C	43.63031800	34.74227800	46.00882000
C	44.96314600	34.98796400	45.28450800
C	44.84069200	35.30056100	43.80806500
N	45.29584800	36.50173800	43.43750800
O	44.33608800	34.51024700	42.99127500
H	44.79033900	33.39113600	48.24550600
H	43.93901100	36.16873300	47.61991400
H	43.31457600	33.69342200	45.88887300
H	42.82543400	35.35777500	45.57359000
H	45.47963800	35.82142300	45.78041400
H	45.62149300	34.11338200	45.39026100
H	45.34451700	36.76223900	42.44493900
H	45.76587400	37.11827200	44.09912400
C	51.82906800	33.65800700	49.29998600
C	51.67976400	33.84110000	47.77978100
O	51.52612200	32.64340800	47.03438600
H	52.79330600	33.20112000	49.57097000
H	50.84122500	34.53010600	47.58631600
H	52.59028800	34.33191100	47.39211300
H	50.60416500	32.33111300	47.06356600
C	49.79104000	39.12700100	48.44103500
C	49.51522700	38.41207800	47.11151900
C	50.55873800	38.69670600	46.05524300
C	50.65533200	39.98794100	45.50744400
C	51.46872900	37.73599800	45.59635500
C	51.61852300	40.31657700	44.56275900
C	52.44293100	38.04554400	44.63746200
C	52.53534100	39.34832300	44.11630800
O	53.45757900	39.73119800	43.21633800
H	50.12510200	40.16275900	48.26512600
H	49.41121200	37.32804100	47.26998700
H	48.54441900	38.75243800	46.72565200
H	49.95003200	40.75721600	45.83782700

H	51.41775900	36.72033400	45.99990000	H	46.52600200	28.87779900	43.64185100
H	51.69196600	41.32177100	44.14632500	H	46.38616400	28.54596300	45.37172400
H	53.15487000	37.28582500	44.30378000	C	46.96511400	28.39858300	39.70156200
H	54.03489700	38.98180000	42.90041300	C	48.44402800	28.18573700	39.36154500
C	54.54498900	40.14801600	38.94597800	C	49.32739200	28.03874100	40.60274500
C	53.34808400	39.78369700	39.82540000	C	49.37789900	29.27600900	41.49965400
O	52.23210700	40.63728200	39.59792800	C	50.15075500	28.99291400	42.77980000
H	55.33025500	39.38297300	39.06352600	N	50.15068400	30.14538400	43.70488500
H	53.06483900	38.73167800	39.60340300	H	46.76249500	29.41585700	40.07124500
H	53.63648900	39.80846700	40.88467700	H	48.54786200	27.27212400	38.75291200
H	51.76255200	40.76634200	40.43741400	H	48.82329900	29.00738100	38.73292800
C	57.82599200	39.04396400	43.41102700	H	48.96202600	27.18435500	41.20104900
C	56.84216300	39.41547100	42.27884800	H	50.35212600	27.76956500	40.29485900
C	56.14524400	38.17125000	41.74339600	H	49.82977200	30.12615800	40.95863200
O	56.82438200	37.42744500	40.98963300	H	48.35943600	29.59145400	41.77689300
O	54.94636600	37.91473300	42.08250300	H	49.68948000	28.15823300	43.32500000
H	58.01225500	37.95792200	43.41315800	H	51.19547700	28.71450500	42.56778000
H	56.09551500	40.14310900	42.62645000	H	50.83784800	30.86004500	43.42169600
H	57.38618700	39.85496600	41.42970700	H	49.17510500	30.60059700	43.74936700
C	54.90491800	31.34004700	42.21901300	H	50.32174300	29.80702700	44.66879200
C	54.24483800	32.67386800	41.89041900	C	45.80663700	28.83403900	36.11091300
C	52.74441300	32.72127300	42.09641800	C	47.26575100	29.28444700	36.07291200
O	52.06569500	31.73222400	42.31346200	C	47.57592900	30.57874200	36.84126500
O	52.15812500	33.90616400	41.96448000	C	47.04838400	31.86753300	36.23180200
H	54.32661400	30.50716800	41.79317600	O	45.79622700	32.00219500	36.05520600
H	54.70743800	33.51424200	42.43296300	O	47.88466200	32.76786500	35.94965000
H	54.39123500	32.90376200	40.82276700	H	45.16675500	29.50551600	35.51987100
C	50.15103900	26.19807000	46.21390700	H	47.90980600	28.49543600	36.49088900
C	50.27373900	27.61860900	46.77316400	H	47.59766000	29.39707000	35.02833900
O	49.62258400	28.54392700	45.90610400	H	47.15375800	30.51632200	37.85897900
H	49.12684200	25.80916200	46.32164600	H	48.66405800	30.68699000	36.93839900
H	49.83273600	27.66527600	47.78479700	H	49.04546700	32.36079300	39.21610000
H	51.34294300	27.88602800	46.87893800	C	52.34805300	24.35399600	37.72098300
H	49.33800100	29.36859400	46.36152300	C	52.87194800	25.63799800	38.39000900
C	45.55198000	26.43195600	42.70694900	C	52.06756500	26.84406400	37.87404600
C	45.41534900	27.03730500	44.11231200	C	52.49775500	28.21723300	38.38308600
C	45.39503900	25.95039800	45.19054000	N	51.79596000	29.26221000	37.64863400
C	46.51433100	28.06837400	44.38796000	C	51.70594600	30.54006000	38.01006400
H	44.71142400	25.76295200	42.46129200	N	52.04165600	30.96328800	39.26009300
H	44.44646500	27.57141300	44.15147200	N	51.30114100	31.45625800	37.13851300
H	46.35027700	25.39755400	45.20357700	H	52.27541300	24.48454700	36.62974100
H	45.25048600	26.38398300	46.19317000	H	52.80113700	25.56002000	39.48888700
H	44.58848900	25.21934900	45.01850700	H	53.93997300	25.77833800	38.14975600
H	47.51362500	27.60366000	44.39926500	H	52.13106300	26.86115400	36.77223700

H	50.99693500	26.70381000	38.10353800
H	52.28359200	28.29971600	39.46323200
H	53.59167900	28.33927800	38.26854900
H	51.45771300	29.08933000	36.66913000
H	52.07911700	30.30908000	40.03066800
H	51.75123100	31.91419700	39.48855200
H	51.14676600	31.27322400	36.11769100
H	51.20813600	32.40723000	37.49786400
C	50.01093700	30.57595900	31.79202800
C	49.92395300	29.58610400	32.95000900
C	50.52798200	30.02999200	34.28308900
O	50.79146200	31.26594300	34.45838700
O	50.71092900	29.14376000	35.14683500
H	49.42551200	31.48755300	31.98799400
H	48.87071000	29.32609000	33.15795500
H	50.40634000	28.63032400	32.69140600
C	51.92999100	33.85205900	31.53908800
C	51.21077500	35.07390300	32.10792100
C	50.98006000	35.02489500	33.58425600
C	51.06221700	36.02875400	34.52195900
N	50.52902200	33.89858400	34.26164500
C	50.33048000	34.20790600	35.54262900
N	50.64372500	35.48978300	35.72664200
H	52.10320000	33.97108200	30.45977100
H	51.76793500	35.99821600	31.89156100
H	50.23280800	35.18712000	31.60696000
H	50.49483700	32.88512800	33.97928600
H	51.37133200	37.06593700	34.40536400
H	49.91114500	33.52587400	36.27112400
H	50.59061000	35.92225700	36.66782900
C	54.69393600	32.85186900	34.18399700
C	54.56607900	32.58910200	35.74401500
C	54.13861800	33.79896400	36.56317900
C	54.44523300	33.55658000	38.03918100
N	53.81544900	34.52083300	38.91918700
C	54.40474500	35.31049100	39.82110600
N	55.70695900	35.38130100	40.00163000
N	53.58089000	36.01350900	40.67494300
H	55.31221200	33.74559000	34.00067300
H	53.86531100	31.75629000	35.91745100
H	55.55628600	32.24734200	36.09377300
H	54.66114900	34.71104400	36.22817700
H	53.06041900	33.99044600	36.44510700
H	54.08782700	32.55105400	38.32370500

H	55.53401800	33.54744200	38.19556500
H	52.78917200	34.45197500	38.89617900
H	56.29585200	34.82974400	39.38973900
H	56.18343700	36.26428100	40.50891000
H	52.72378900	36.35301400	40.23642600
H	54.07890900	36.75544500	41.24451700
C	47.10498400	38.23007800	31.48484800
C	47.66051700	36.83545500	31.78131800
C	47.12739400	36.15478600	33.05045600
C	45.59287400	36.00059700	33.06990400
C	45.10419100	34.71108200	33.72169600
N	45.65239200	34.50205700	35.07967400
H	46.00792800	38.26538000	31.57089800
H	47.43531600	36.17299900	30.92610600
H	48.76097400	36.88898500	31.83994000
H	47.60815700	35.15992500	33.11792500
H	47.45996100	36.70858200	33.94669100
H	45.11800500	36.85689100	33.57071700
H	45.20771200	35.98836900	32.03638100
H	44.00775000	34.70064100	33.80047300
H	45.40791600	33.83691000	33.12380400
H	45.54368000	33.49672500	35.43232900
H	46.67186600	34.62155600	35.09844200
H	45.06738000	37.09547200	37.34555000
C	49.69964900	40.63346900	33.03442400
C	50.51712800	40.13833100	34.22578300
C	49.67038400	39.20964600	35.08920600
O	51.65151300	39.37186400	33.81875400
H	48.68138300	40.88921100	33.37239400
H	50.84618100	40.99460600	34.84671500
H	52.24713900	39.94046200	33.31384200
H	48.78437300	39.73245600	35.47329400
H	49.32303800	38.34960400	34.49536900
H	50.25072700	38.83683600	35.94645900
P	47.75985600	31.79702800	45.42780200
C	48.63976800	33.99739700	43.03377200
C	48.82789500	35.26685400	42.20353400
C	48.70934800	34.33176000	44.58431100
C	49.60241900	36.33676300	42.94868500
C	48.68505100	36.73983400	44.05862700
C	48.25390600	35.74783200	44.85404300
O	47.88888700	33.46662000	45.35575100
O	46.34670100	31.66752100	46.03430900
O	48.92458700	31.26149000	46.25589900

O	47.76671600	31.26940100	43.94811000
O	47.41583500	33.43498400	42.66523300
O	49.54440200	35.11350300	40.94724900
C	48.06857700	38.10315900	43.99299800
O	47.12716700	38.37461800	44.80145200
O	48.42967700	38.88101100	43.06957900
H	47.42614100	32.52530200	43.07151700
H	52.77372700	34.64084900	41.69546500
H	50.55292600	35.92432400	43.32083500
H	49.84558200	37.18361200	42.30195700
H	47.82866100	35.62127600	41.91059300
H	49.48350300	33.31357300	42.82094500
H	49.76544000	34.20944100	44.89701200
H	47.56621600	35.90907500	45.67993700
P	46.43162300	35.04305700	38.32200000
C	49.43189300	34.25178600	40.00533500
C	48.64453200	33.08181000	39.93634900
C	50.40253600	34.66546500	38.90268000
O	46.88574300	36.00341100	39.42118800
O	45.16500200	35.54750900	37.55041600
O	46.25270500	33.56595500	38.82788700
O	47.65529000	34.99024400	37.24430500
O	51.22654200	33.74772900	38.60395500
O	50.38484500	35.82830400	38.48724800
H	47.53306700	33.36206200	39.44436400
H	48.29974000	32.67924900	40.89252900
H	42.73709900	45.00217700	44.23308300
H	42.06260200	46.07429300	45.48594200
H	43.65561200	40.63878500	34.40288600
H	42.44870400	41.41233500	35.46219900
H	47.35785600	38.53059200	30.45548100
H	50.03140900	41.51351900	32.47375400
H	49.59673000	39.80451200	32.31613000
H	47.52287700	39.00071300	32.14100300
H	40.58121000	24.81725500	39.39195600
H	40.38209500	25.17565900	41.14810900
H	38.50189900	35.38756200	44.47318700
H	38.70023500	35.26658700	42.70970900
H	51.35186600	24.07473500	38.09753600
H	53.04422100	23.52998300	37.90887600
H	50.85411600	25.50835700	46.69258800
H	50.39482400	26.20688600	45.13806200
H	55.91253000	31.30921000	41.77787900
H	55.02478900	31.15420200	43.29145300
H	54.97980300	41.11894900	39.20528000
H	54.25047800	40.17822200	37.88406900
H	58.81859400	39.49865900	43.32693400
H	57.41618800	39.30426900	44.39833800
H	51.73129400	34.63181300	49.79108800
H	51.02781900	33.00750200	49.68842100
H	48.89445500	39.18829300	49.06668000
H	50.58612100	38.62681900	49.01607100
H	46.89412600	29.49649600	49.27824400
H	42.84609600	34.83674200	48.04681400
H	55.19979100	31.99649400	33.71014800
H	53.74425100	32.98319000	33.65504900
H	49.63339300	30.10838500	30.87670100
H	51.33833700	32.93873500	31.68259200
H	51.05409400	30.87162400	31.60681100
H	52.89627400	33.69833800	32.03065500
H	48.76811200	44.19230300	38.59730600
H	47.36019100	43.57729800	39.46682100
H	48.09328900	42.96946300	41.09425700
H	47.59319800	43.47524900	42.72420800
H	41.15738100	34.61071900	35.16515300
H	42.31607300	38.23454100	40.67181400
H	45.68718300	27.81199600	35.73587800
H	45.42586100	28.84306200	37.14295700
H	46.48507200	25.84929700	42.61667300
H	45.59406100	27.23433500	41.96303300
H	46.67292200	27.69772100	40.48962700
H	46.31855300	28.22962600	38.83103900
H	45.28912800	35.12759500	35.83961900
H	47.72920800	34.10268100	36.79070900

EP(-1.2)

C	47.75562600	43.77625100	38.46528100
C	47.90506100	42.47493500	37.66249200
C	48.80207200	41.37695000	38.28041800
C	48.10098700	40.30146700	39.13453400
C	48.99320700	39.06739600	39.25291900
N	48.46811600	38.01718700	40.17998000
H	47.07674700	44.48105800	37.96120800
H	46.90435800	42.05096800	37.45850300
H	48.31713100	42.73505700	36.67221200
H	49.31573100	40.85800500	37.45665400
H	49.61519900	41.82362900	38.87572600
H	47.88761700	40.68465500	40.14201600

H	47.13842300	40.01259200	38.67319500	C	40.57990400	36.67518700	35.47152100
H	49.11504200	38.58721800	38.27076700	H	42.48122500	35.63729800	35.69071500
H	49.99599200	39.35452300	39.60206300	H	40.87132100	37.53798300	36.09160500
H	47.45137500	37.82064200	40.07617400	H	40.71329300	36.95206800	34.41495300
H	48.98098100	37.13595900	40.00538900	H	39.51467200	36.48295000	35.66077600
H	48.60475800	38.26975100	41.20012000	N	42.22936100	34.45850100	37.90859100
C	47.94695900	42.62101700	42.12201100	C	42.13198900	34.10218400	39.30154800
C	49.26055000	42.04958600	42.65930900	C	42.20113400	35.27324600	40.28608200
O	49.77474700	41.00387200	41.85826500	O	41.71814000	35.17782400	41.40452900
H	47.15173300	41.85626300	42.11861200	H	43.17289300	34.58292500	37.52699800
H	50.01338300	42.85677500	42.69700700	H	41.18269900	33.58534500	39.49315200
H	49.12266100	41.69992800	43.69805500	H	42.96611200	33.43149700	39.55748900
H	49.53366600	40.15929900	42.29298700	N	42.89106300	36.36142300	39.86890100
C	42.49293500	45.08392200	45.30404600	C	43.11198900	37.48603200	40.74453000
C	43.72500800	44.78706300	46.18802300	C	44.44048200	38.21777500	40.45940300
C	44.03711100	43.27458400	46.16019900	C	44.59266300	39.43483700	41.36483500
C	45.28062000	42.82827500	46.94539100	O	45.59550400	37.41103300	40.66378700
N	45.36347100	41.38138100	47.17701100	H	43.12752900	36.45212700	38.88839900
C	45.94502300	40.46167600	46.37956100	H	43.10022600	37.10752300	41.77635300
N	46.48093700	40.79438900	45.20435300	H	44.42109400	38.55136900	39.40728900
N	45.99367900	39.17387700	46.74749700	H	45.69874300	36.63628800	40.03793300
H	41.69315100	44.35268600	45.50749800	H	43.75422700	40.13633700	41.23729900
H	44.59621600	45.37043300	45.84336700	H	44.63411200	39.12386100	42.42059800
H	43.53195500	45.09613900	47.22957600	H	45.52896300	39.96394000	41.13126200
H	43.16684200	42.73373500	46.57083300	C	38.77099300	34.70900100	43.65697500
H	44.12725400	42.93602500	45.11212500	C	40.18800100	34.06400200	43.77702700
H	46.20927800	43.15839600	46.45164900	C	40.39605900	32.89334500	42.81444500
H	45.28722000	43.30424100	47.93823200	C	41.84321800	32.41862400	42.70626600
H	44.91170400	41.02508000	48.01094900	N	42.31549800	31.77534700	43.94264000
H	46.36748600	41.71888400	44.81483900	C	43.57787400	31.41337900	44.18733900
H	46.93511500	40.01895400	44.66547700	N	44.56147000	31.77905600	43.32744400
H	45.82267400	38.85258300	47.69522100	N	43.89755600	30.64424200	45.23288600
H	46.52104000	38.54413300	46.12672800	H	37.99347300	33.92538300	43.63524900
C	42.94444100	40.46516600	35.22516000	H	40.95386200	34.82342200	43.56842100
C	43.23405800	39.62319900	36.46538200	H	40.34643000	33.72421100	44.81785100
C	43.91277000	38.28668200	36.20721800	H	39.73354700	32.04640600	43.07987900
O	44.32008700	37.92504500	35.10862400	H	40.10604100	33.21608000	41.80433800
O	44.02837400	37.56252500	37.30226500	H	41.94007400	31.69375600	41.87714200
H	42.29443000	39.91347100	34.52860700	H	42.47730100	33.28447300	42.48200700
H	43.88527000	40.16252800	37.17673100	H	41.61825400	31.52457900	44.63493000
H	42.31549000	39.40343600	37.03476200	H	44.51064600	32.80651800	43.08767800
C	41.40497200	35.44396400	35.83107300	H	45.50374300	31.51874900	43.65173200
C	41.14456600	34.97486500	37.26024200	H	43.21981600	30.50185500	45.97568300
O	40.03840200	35.04055400	37.77257300	H	44.86676700	30.81325200	45.57755800

C	41.02904900	25.29701400	40.27306300
C	41.20598200	26.91098400	40.03498000
C	42.34543900	27.23850000	39.06428000
C	42.54834900	28.71780500	38.69860100
N	43.29254500	29.49328300	39.69756200
C	43.97365400	30.62946600	39.42422000
N	44.01871800	31.12597400	38.18602900
N	44.63540100	31.29544900	40.37319100
H	42.00539700	24.83491900	40.48211600
H	40.25351200	27.31759600	39.65815200
H	41.38652100	27.37715600	41.01950900
H	43.30081100	26.83439800	39.44372600
H	42.15495200	26.70978700	38.11546600
H	43.12552000	28.74531700	37.76180100
H	41.57216600	29.19504500	38.49634900
H	43.30198700	29.16464500	40.65607500
H	43.60484000	30.64092600	37.40518400
H	44.60815200	31.98320400	38.06446800
H	44.51549600	31.14129400	41.37746500
H	45.14526800	32.13929900	40.05679000
C	46.45299300	30.41006300	48.86606000
C	46.81015300	31.67290300	49.65536800
O	46.92820400	31.70731300	50.86743200
C	44.93092100	30.26414100	48.75192700
O	44.33973500	31.34306800	48.03771700
H	46.86963600	30.49450500	47.85701400
H	44.70396500	29.29972800	48.25291100
H	44.47065500	30.23202300	49.75248600
H	44.98554800	31.58414600	47.33505500
N	46.87965900	32.79305100	48.85983000
C	46.96035400	34.15334900	49.35860000
C	45.89861500	35.06786800	48.70727300
O	46.02771400	36.28981500	48.68312900
C	48.33866200	34.77686200	49.14582200
O	48.62512000	34.97320600	47.77818900
H	46.73244500	32.65309800	47.86160700
H	46.76531200	34.10424100	50.44372800
H	48.36204800	35.76805100	49.61915800
H	49.08977800	34.13325600	49.63506100
H	48.38762300	34.18294200	47.26349100
N	44.84811000	34.41259400	48.16369200
C	43.75797500	35.09203700	47.49700800
C	43.65911800	34.76044700	46.00353100
C	44.97705100	35.10082200	45.29526800

C	44.88505800	35.28056700	43.79413500
N	45.40398500	36.41674300	43.31442800
O	44.35024200	34.43930600	43.05112800
H	44.76275200	33.40690500	48.31087700
H	43.92902100	36.16982100	47.62941100
H	43.41287500	33.69603600	45.86347000
H	42.82077400	35.33205400	45.57132500
H	45.39121500	36.01627500	45.74313200
H	45.71336200	34.30526000	45.48393600
H	45.46850600	36.57923800	42.30293200
H	45.91433100	37.06271200	43.91335100
C	51.82904800	33.65800800	49.29998400
C	51.64317200	33.85194700	47.78517500
O	51.41092500	32.66552900	47.03682600
H	52.79977100	33.20512500	49.55296300
H	50.82807900	34.57499000	47.62049300
H	52.56139000	34.29985800	47.36681300
H	50.49300900	32.36338400	47.14897100
C	49.79101200	39.12700500	48.44101100
C	49.54386000	38.40317600	47.10900500
C	50.60313100	38.68941000	46.06703600
C	50.66932100	39.96261800	45.47388800
C	51.55596800	37.74634600	45.65953100
C	51.63614100	40.28711100	44.53058200
C	52.53458100	38.05105500	44.70382300
C	52.59137300	39.33427600	44.12919200
O	53.50934800	39.71245300	43.22650100
H	50.12036800	40.16404300	48.26150700
H	49.44268100	37.31953600	47.27315600
H	48.57903600	38.73491000	46.70051600
H	49.93496100	40.72071200	45.76577100
H	51.53221000	36.74477300	46.09971400
H	51.68219200	41.27794200	44.07721100
H	53.27321000	37.30313800	44.40393600
H	54.09243400	38.96258800	42.90430500
C	54.54498500	40.14801700	38.94597000
C	53.36052000	39.76747300	39.83471400
O	52.22964500	40.61296000	39.62108300
H	55.33481800	39.38602700	39.05082200
H	53.08248900	38.71752300	39.61291600
H	53.65902800	39.79088300	40.89117200
H	51.78839200	40.76305300	40.47227900
C	57.82600000	39.04399300	43.41101700
C	56.98358400	39.14289100	42.11474700

C	56.15596600	37.88797600	41.78513800
O	56.73938700	36.95717800	41.18357800
O	54.91865700	37.84998200	42.10085800
H	58.01362700	37.98581700	43.65870900
H	56.31104500	40.01261100	42.16315100
H	57.65423600	39.28036700	41.25338400
C	54.90497200	31.34000200	42.21900100
C	53.63846900	32.14319000	41.95281300
C	52.40238600	31.34085700	42.26744900
O	52.39843100	30.13601300	42.41978500
O	51.23432200	31.98614400	42.33575700
H	54.86914800	30.36953300	41.70565200
H	53.61740200	33.08939500	42.51863800
H	53.55666200	32.43238400	40.88922400
C	50.15102500	26.19805600	46.21391800
C	50.04164200	27.50738500	46.99475000
O	49.23059200	28.42263400	46.26313700
H	49.16909900	25.70671500	46.13135700
H	49.59690000	27.31798700	47.98831500
H	51.04882600	27.93316300	47.16484000
H	49.05013900	29.26727300	46.73241800
C	45.55198000	26.43195600	42.70694900
C	45.22778500	27.00626800	44.09388100
C	45.13909100	25.89879100	45.14760200
C	46.23572100	28.08138400	44.51112400
H	44.80022500	25.69406200	42.38268700
H	44.23451200	27.49151900	44.02979100
H	46.11098200	25.38706400	45.25576800
H	44.86885900	26.30624300	46.13486500
H	44.38893900	25.13787400	44.87808100
H	47.24174400	27.65832400	44.66036900
H	46.31293400	28.89489100	43.77317100
H	45.95546700	28.54434200	45.46938700
C	46.96507100	28.39856900	39.70153700
C	48.45540100	28.22938600	39.38840100
C	49.27741000	27.96279800	40.64880900
C	49.16592700	29.07287500	41.68824900
C	49.75006400	28.66122000	43.02489000
N	49.72704100	29.78059900	43.98528500
H	46.72675200	29.41157500	40.06062600
H	48.59830200	27.39065200	38.68783000
H	48.84041400	29.12448300	38.87067000
H	48.93455900	27.01277300	41.09775500
H	50.33808500	27.80275400	40.38915100
H	49.67010200	29.98219200	41.32830000
H	48.11203700	29.35015200	41.84603400
H	49.15892600	27.84630800	43.46510700
H	50.78875200	28.32051300	42.92139100
H	50.50267500	30.42749500	43.82002800
H	48.82122900	30.35114700	43.93868000
H	49.76234400	29.39829500	44.95646600
C	45.80671300	28.83397500	36.11103800
C	47.27513000	29.26315300	36.10401400
C	47.47894900	30.77268900	36.23867300
C	47.25981900	31.51668500	34.93080500
O	47.07969600	30.98221700	33.86336800
O	47.30203900	32.85714500	35.00270600
H	45.21549500	29.47977200	35.43991300
H	47.82015500	28.75527700	36.90977200
H	47.75382000	28.93654400	35.17077700
H	46.83644400	31.21887700	37.01938300
H	48.51435300	30.98831400	36.54565900
H	48.45943200	32.48415500	39.30493700
C	52.34798000	24.35398100	37.72105000
C	52.87197300	25.63798600	38.39002100
C	52.04568600	26.84325800	37.88312700
C	52.50753200	28.23289000	38.32851000
N	51.75606800	29.28657600	37.64367500
C	51.36428700	30.44478300	38.16102500
N	51.51804800	30.72824000	39.47737500
N	50.83931600	31.39066100	37.37067900
H	52.27474700	24.48454200	36.62997300
H	52.81609500	25.55922200	39.48923200
H	53.93508300	25.78760100	38.13423900
H	52.04906300	26.82986800	36.77950600
H	50.98894800	26.71474100	38.17488700
H	52.39685300	28.33255400	39.42029500
H	53.58491800	28.35651200	38.11467500
H	51.49563100	29.17275900	36.62541500
H	51.73088200	29.98842800	40.13609000
H	50.98411700	31.50624800	39.85744900
H	50.80051800	31.28377800	36.33085200
H	50.46064600	32.24926700	37.77729000
C	50.01099800	30.57590500	31.79200100
C	49.93892400	29.59265300	32.95733100
C	50.48681000	30.06101900	34.30499800
O	50.53847500	31.31330300	34.56457500
O	50.81056900	29.17402800	35.12610400

H	49.39841600	31.46826900	31.99278600
H	48.88173100	29.33919400	33.14543300
H	50.44716300	28.64622200	32.71888400
C	51.93002400	33.85213600	31.53899100
C	51.22913700	35.11587700	32.01668200
C	50.88846300	35.05312900	33.46434300
C	50.87921200	36.02981500	34.43200800
N	50.45888500	33.88622900	34.08069800
C	50.18860200	34.13521700	35.35311500
N	50.43051700	35.42484300	35.59611800
H	52.11674600	33.90225900	30.45582800
H	51.84034400	36.01384900	31.84123900
H	50.29950900	35.25917100	31.43882700
H	50.44431000	32.87392500	33.79570700
H	51.16367600	37.07878800	34.36291400
H	49.83452200	33.41477200	36.06768700
H	50.31917900	35.81347100	36.53506900
C	54.69400000	32.85188100	34.18397500
C	54.56601700	32.58907000	35.74400200
C	54.03331100	33.80456000	36.52013400
C	53.78693500	33.50671000	38.00662900
N	53.31215700	34.63192200	38.80369800
C	54.01625700	35.31550800	39.74599000
N	55.32885300	35.14293200	39.89902100
N	53.36086300	36.14657500	40.55622000
H	55.30491800	33.75136300	34.00274800
H	53.89767200	31.72548900	35.90636200
H	55.55806300	32.30472800	36.13311000
H	54.73597500	34.65106000	36.42794500
H	53.08372800	34.14413100	36.06920500
H	53.04414900	32.69765000	38.08668600
H	54.70395600	33.11767500	38.47547500
H	52.35904000	34.95680700	38.66258300
H	55.83918000	34.71662100	39.13621900
H	55.88749900	35.83129900	40.52035500
H	52.34746900	36.20353200	40.48460500
H	53.90116900	36.78417700	41.19587000
C	47.10497300	38.23021400	31.48466700
C	47.85053900	36.89318000	31.53088700
C	47.34113800	35.94970000	32.62283100
C	45.89565200	35.49832400	32.37348800
C	45.28837100	34.68703200	33.51011000
N	45.48536800	35.39734700	34.79658200
H	46.03882400	38.12941200	31.73388600

H	47.75654500	36.38293600	30.55654400
H	48.92806300	37.07616300	31.67558800
H	47.99135000	35.06297800	32.71114500
H	47.41962900	36.48512700	33.58662800
H	45.25779000	36.37799200	32.19315400
H	45.85304100	34.89225000	31.45409100
H	44.20642700	34.55277600	33.36778800
H	45.76194100	33.70284400	33.60523200
H	45.05340300	34.98943500	35.67364700
H	46.47744000	35.42788000	35.05802900
H	44.47706500	36.63048500	37.21512300
C	49.69963700	40.63346600	33.03440700
C	50.53261100	40.13465800	34.21541400
C	49.66569300	39.31250700	35.16305400
O	51.58820600	39.26724300	33.79631700
H	48.68748700	40.89751000	33.38419300
H	50.95341700	40.98921400	34.77939700
H	52.21350700	39.77063600	33.25890900
H	48.86452200	39.92970600	35.59144200
H	49.19761200	38.47404000	34.62154000
H	50.27276100	38.91097700	35.98856800
P	47.58765600	31.82183300	45.55315000
C	48.73182900	33.83357600	43.27285200
C	49.34877200	34.89881800	42.35388300
C	48.73690500	34.27094800	44.76280500
C	49.97479000	36.06988000	43.08784100
C	48.90156600	36.59935100	44.00656700
C	48.33408000	35.71848200	44.84954800
O	47.83704000	33.48295000	45.53582400
O	46.16846100	31.78529100	46.16380300
O	48.71657700	31.18974900	46.36156000
O	47.52395400	31.30769700	44.07102500
O	47.43921900	33.56257900	42.80275400
O	50.32307500	34.28716200	41.45465700
C	48.34951100	37.97553800	43.78076800
O	47.39421000	38.37674400	44.51333400
O	48.79463100	38.63693100	42.79945800
H	47.25086800	32.66349900	43.16045600
H	51.21615800	32.95371700	42.11367600
H	50.85245300	35.73859700	43.66527700
H	50.31432100	36.84174400	42.38892100
H	48.52287300	35.26308200	41.72612500
H	49.34908900	32.92563800	43.21974600
H	49.76523900	34.12916700	45.15154000

H	47.56060900	35.99957000	45.55943200
P	46.02696300	34.57942700	38.17638700
C	49.74617600	33.81807800	40.29626700
C	48.94353900	32.74432800	40.24547300
C	49.97096300	34.68782500	39.09066800
O	46.55931300	35.55300200	39.21216600
O	44.86374300	35.17209000	37.28235100
O	45.68623200	33.14627400	38.61702900
O	47.25213300	34.43085700	37.02923000
O	49.69932000	34.14929000	37.97241400
O	50.40223900	35.85217700	39.23706400
H	47.29444900	33.25288000	35.91900700
H	48.68326300	32.17675400	41.13589900
H	42.74389400	45.00723200	44.23421800
H	42.06267400	46.07433700	45.48587300
H	43.87458800	40.69351100	34.68288400
H	42.44875400	41.41238300	35.46211100
H	47.14596900	38.65997500	30.47067400
H	50.03141100	41.51352200	32.47376000
H	49.58159700	39.80438500	32.31907300
H	47.52290200	39.00059400	32.14113300
H	40.57728400	24.81913700	39.39273700
H	40.38210500	25.17566100	41.14811600
H	38.50189700	35.38756000	44.47318800
H	38.69767600	35.26602800	42.70940200
H	51.35224600	24.07399000	38.09843900
H	53.04426400	23.53000700	37.90881600
H	50.85412900	25.50836400	46.69257900
H	50.52236200	26.39319000	45.19438300
H	55.78764400	31.89591500	41.87059100
H	55.02476100	31.15422500	43.29146100
H	54.97980700	41.11894700	39.20528600
H	54.24060000	40.18237600	37.88647600
H	58.81860000	39.49864600	43.32694900
H	57.30330500	39.49269000	44.26870600
H	51.73131800	34.63181400	49.79108900
H	51.03572600	33.00623800	49.70249700
H	48.89447000	39.18828600	49.06670100
H	50.58700200	38.63754200	49.02419700
H	46.89417100	29.49649700	49.27819700
H	42.84608800	34.83674600	48.04680300
H	55.20941900	32.00328400	33.70852300
H	53.74423000	32.98325300	33.65510300
H	49.63336300	30.10842700	30.87669200
H	51.31431400	32.96068800	31.71338200
H	51.04764300	30.89146800	31.59629600
H	52.89623700	33.69822900	32.03069700
H	48.73100200	44.27317300	38.59178900
H	47.36016600	43.57721900	39.46679200
H	48.09329900	42.96955500	41.09428800
H	47.61063000	43.47808000	42.73044900
H	41.15737600	34.61074200	35.16512600
H	42.31606700	38.23452300	40.67169600
H	45.68712100	27.81203200	35.73579900
H	45.37090900	28.89864000	37.11873700
H	46.53538600	25.93059400	42.70725000
H	45.59405300	27.23433000	41.96302600
H	46.67296300	27.69773800	40.48965700
H	46.33673200	28.20890000	38.82132500
H	45.11936800	36.38093900	34.78605600
H	48.15021800	34.41518100	37.45851000
